# Toward Pore‐Engineered 3D‐Printed Materials for Sorption Water Harvesting, Interfacial Evaporation, and Radiative Cooling

**DOI:** 10.1002/adma.74850

**Published:** 2026-08-31

**Authors:** Dejan J. Trajkovski, Higgins M. Wilson, Abdallah Y. M. Ali, Jiaxing Xu, Ruzhu Wang, Primož Poredoš

**Affiliations:** ^1^ Laboratory for Sustainable Technologies in Buildings Faculty of Mechanical Engineering University of Ljubljana Ljubljana Slovenia; ^2^ Institute of Refrigeration and Cryogenics Engineering Research Center of Solar Energy (MOE China) Shanghai Jiao Tong University Shanghai China

**Keywords:** additive manufacturing, atmospheric water harvesting, interfacial solar evaporation, materials science, nanotechnology, process engineering, radiative cooling

## Abstract

Water scarcity has intensified interest in passive, sorption‐based atmospheric water harvesting (SAWH), interfacial evaporation (IE), and radiative cooling (RC) technologies that operate at the energy–water–air nexus through coupled heat and mass transfer. Although these approaches are frequently associated with hierarchical porous materials, their performance depends more fundamentally on mesoscale architecture, interfacial design, and system‐level integration than on intrinsic nanoscale porosity alone. Additive manufacturing (AM) offers a versatile platform for engineering such architectures, providing precise control over geometry, connectivity, and functional gradients, while also enabling post‐processing routes to introduce additional porosity where required. Unlike existing reviews that primarily focus on material systems or individual applications, this review establishes a unified manufacturing‐ and pore‐architecture‐centric perspective across SAWH, IE, and RC, emphasizing how AM processing governs transfer behavior and multifunctional performance. This review focuses on the role of AM in structuring porous and semi‐porous materials for SAWH, IE, and RC, with particular emphasis on extrusion‐based methods such as direct ink writing that offer broad material compatibility and process flexibility. We assess the most relevant 3D printing technologies for these applications and discuss their incorporation into operational devices. To address the absence of standardized, application‐specific evaluation methods, we introduce a comprehensive characterization framework linking printing resolution, architectural features, and functional performance, including sorption dynamics, thermal robustness, and multifunctional coupling. Finally, we discuss future opportunities for composite design, system‐level integration, and artificial‐intelligence‐assisted optimization to advance scalable water and thermal management solutions.

## Graphical abstract

Water harvesting, radiative cooling, and interfacial solar evaporation are fundamentally governed by coupled heat, mass, and light transport processes. These processes are mediated by pore architecture, including pore size, connectivity, and hierarchical organization. The engineering of multifunctional porous systems for these real‐world applications is possible by linking bioinspired material design and transport mechanisms with additive manufacturing‐enabled structural control.

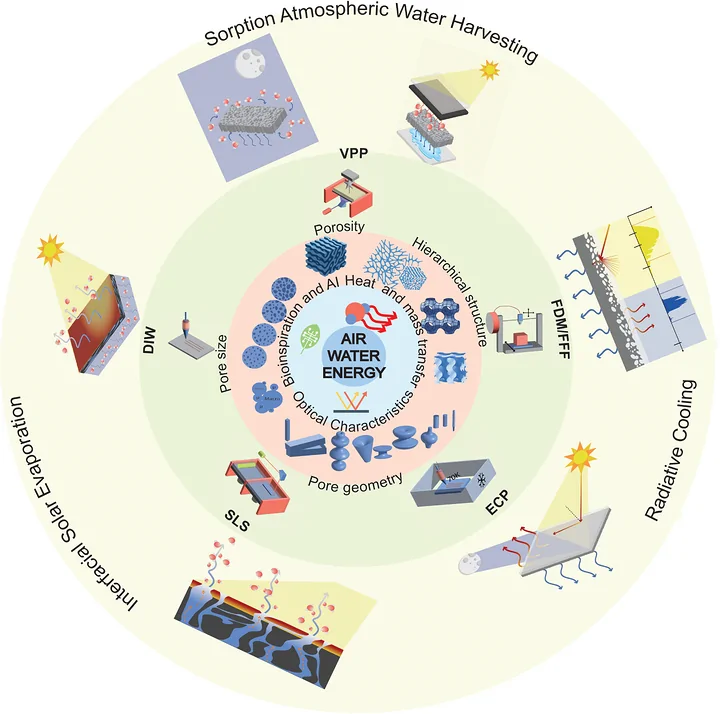

## 1 Introduction

Global freshwater scarcity, combined with the increasing unreliability of conventional sources, has driven strong interest in technologies that generate clean water directly from ubiquitous environmental resources [1, 2, 3, 4]. Among emerging strategies, sorption‐based atmospheric water harvesting (SAWH), radiative cooling‐based AWH (RAWH), and interfacial solar evaporation (ISE) for desalination have attracted particular attention. These approaches enable freshwater production with minimal external energy input and are well suited for decentralized deployment [5]. In parallel, radiative cooling (RC), while not a water‐generating technology per se, plays a critical role at the energy–water nexus by passively reducing cooling loads and associated water and energy consumption in built environments. Notably, RC can also directly contribute to water production in radiative (dew) water harvesting systems through nocturnal condensation, albeit via distinct physical mechanisms. Despite differences in function and operating principle, all these technologies share a common design paradigm: efficiency is dictated not only by material chemistry but, more critically, by the architecture of porous and interfacial networks that mediate coupled water and energy transfer. The size of the pores, their shape, connectivity, tortuosity, directional orientation, and hierarchical arrangement collectively determine vapor diffusion pathways in sorption‐based AWH, water replenishment, and vapor escape dynamics in ISE, and optical–spectral characteristics, such as reflectivity and emissivity in RC materials.

Consequently, pore engineering has emerged as a central design principle with the potential to dramatically improve the efficiency, stability, and scalability of these water‐generating technologies, a capability that is becoming increasingly relevant under accelerating global climate pressures [6, 7]. However, the porous media used today in AWH, ISE, and RC are typically fabricated using conventional methods such as freeze casting, sol–gel chemistry, phase separation, gas blowing, hydrothermal synthesis, or particulate templating. These approaches provide tunability in chemical composition and a degree of control over pore structures, through adjustments in the processing parameters, but inherently produce pore structures that exhibit randomness and/or heterogeneity, limiting their capability for producing specific pore geometries, particularly in synthesis processes that need precise control for transfer efficiency optimization [8, 9]. In contrast, additive manufacturing (AM) offers significantly enhanced capabilities via the utilization of computer‐aided design (CAD) and computer‐aided machining (CAM) processes that directly integrate with the AM process, enabling geometric design of next‐generation pores that achieve optimal trade‐offs between surface area, pressure drop, moisture transfer and heat transfer. AM allows direct and precise positioning of pores, a level of control that is absent from traditional methods of production [10, 11]. Sorbents in atmospheric water harvesting often suffer from slow water uptake because vapor must traverse convoluted, poorly connected pore pathways consistent with challenges identified in continuous AWH research [12]. Interfacial evaporators experience vapor clogging and non‐uniform evaporation fronts when escape channels are insufficiently defined or when water distribution is inconsistent within the evaporative layer. Radiative cooling surfaces rely on carefully tuned scattering behavior in the visible and near‐infrared regions, as well as high emissivity in the mid‐infrared window; both properties are highly sensitive to pore size distribution and the spatial arrangement of voids [13]. When pore morphology is dictated by uncontrolled processing rather than intentional design, performance becomes limited by diffusion bottlenecks, uneven liquid transfer, insufficient light scattering, or excessive thermal conduction. These limitations are not inherent to the materials themselves but result from inadequate control over their internal structure.

AM introduces a powerful means to overcome these limitations by enabling deterministic control over pore architecture across length scales and material classes. Technologies such as direct ink writing, vat photopolymerization, selective laser sintering, fused deposition modeling, and cryogenic extrusion allow the design of pore networks as digital features encoded directly into the printed structure. AM enables precise control over pore size, shape, connectivity, anisotropy, and hierarchical organization, allowing materials to be architected for specific mass transfer or radiative requirements [14]. Unlike traditional synthesis, where pores emerge as artifacts of drying, templating, or phase separation, AM allows pores to be intentionally positioned and shaped to optimize vapor diffusion, water uptake, liquid flow, heat localization, or emissive behavior. This capacity for digital control transforms pore architecture from a by‐product of synthesis into a primary design variable.

From a materials perspective, pore architecture plays a role that extends beyond regulating transfer phenomena, serving instead as a structural intermediary that links manufacturing strategy, effective material properties, and functional performance. The spatial organization of voids defines not only pathways for mass, conductive, convective heat, and radiative heat transfer, but also governs emergent properties, such as permeability, effective diffusivity, thermal conductivity, emissivity, and mechanical compliance, that cannot be attributed only to material chemistry. Treating these quantities as architecture‐derived rather than intrinsic constants enables a design‐driven approach in which material behavior can be deliberately tailored to application‐specific requirements. In this context, AM provides a unique capability to define material properties through geometry by enabling spatially resolved deposition, multi‐material integration, and controlled post‐processing routes that would be inaccessible using stochastic synthesis methods. Accordingly, this review adopts a framework that connects AM design parameters to pore architecture, relates architectural features to effective material properties through targeted characterization, and ultimately maps these properties to performance metrics relevant to AWH, ISE, and RC.

In AWH, AM provides the ability to fabricate sorbents with directional vapor pathways that minimize diffusion resistance and increase the accessibility of sorption domains [15]. Printed hydrogels or composite sorbents can incorporate macroporous channels that facilitate rapid vapor transfer into the interior while retaining microporous regions that provide the requisite surface area for water uptake. By introducing controlled gradients in porosity, AM can tune the balance between fast adsorption at low relative humidity and efficient desorption through enhanced thermal penetration [16]. Such deliberate manipulation of channel size and connectivity allows sorbents to cycle water more rapidly and more efficiently than randomly structured materials. Moreover, AM enables hybrid architectures that integrate sorption materials with photothermal layers or electrical heating pathways, creating monolithic systems capable of passive uptake and actively triggered release with reduced energy input [17].

Interfacial solar evaporation benefits equally from the architectural freedom offered by AM. The performance of ISE devices depends on stable water supply to the evaporating interface, effective heat localization, and unobstructed vapor escape [18]. AM allows the fabrication of evaporators with carefully designed water‐delivery channels that prevent dry spots, avoid flooding, and maintain a continuous liquid film at the interface. The evaporating surface itself can be patterned with hierarchical pores that increase the surface area for evaporation while enhancing light absorption or scattering depending on the material. Vapor‐release channels can be integrated into the structure to reduce backpressure that otherwise suppresses evaporation rates. Because AM does not rely on random foaming or solvent evaporation to form pores, it provides the freedom to design evaporators that sustain high fluxes even when processing saline or contaminated water. Salt accumulation, a persistent challenge for solar evaporators can be mitigated by architecting directional pathways for salt transport or crystallization away from the hot region [12]. Such multifunctional, hierarchically organized structures are extremely difficult to achieve through conventional fabrication.

Radiative cooling also benefits from the precision of additive manufacturing. RC materials rely on a delicate balance between high reflectance in the solar spectrum and high thermal emissivity in the mid‐infrared region, particularly in the atmospheric window (8–13 µm) [13]. Achieving this balance often requires pore sizes tuned to specific scattering regimes or layered architectures that enhance emissive behavior without compromising mechanical integrity [19]. AM allows the creation of periodic or aperiodic pore structures, hollow‐sphere‐inspired geometries, or multi‐layered lattices in ceramics or polymers that exhibit strong broadband scattering and optimized emissivity [20]. The ability to print large‐area, mechanically robust structures with complex curvature or surface texture further enables improved angle‐dependent emissivity and environmental durability [21]. Such design freedom opens possibilities for radiative cooling surfaces that are both high‐performing and manufacturable at relevant scales [22].

Despite these advantages, the application of additive manufacturing to AWH, ISE, and RC remains in an early stage, and the field lacks a cohesive framework that connects AM design parameters, pore architecture, and functional performance [23, 24, 25]. Most reported studies demonstrate promising isolated cases but do not systematically relate printing resolution, ink rheology, curing conditions, or sintering profiles to the resulting pore morphology and transport characteristics. Similarly, the impact of pore orientation, tortuosity, hierarchical composition, or spatial gradients on vapor diffusion, capillary transfer, or optical scattering is rarely quantified in a unified manner [26]. This absence makes it difficult to compare results across studies or extract generalizable design rules.

Recent reviews have highlighted advances in atmospheric water harvesting [27], interfacial evaporation [28], and radiative cooling [29]. However, these studies are typically organized by material systems, photothermal mechanisms, or application‐specific performance metrics. Relatively little attention has been given to AM as a unifying design strategy for controlling pore generation and mesoscale architecture across these three technologies. Current discussions of 3D‐printed structures often focus on isolated demonstrations or fabrication examples without systematically linking AM processing routes to pore morphology, transfer behavior, and multifunctional device performance. This review, in contrast, adopts a manufacturing‐ and architecture‐centric perspective, treating pore architecture as the common design variable connecting SAWH, ISE, and RC. The goal of this work is to establish generalized design principles that extend beyond individual material systems or standalone applications. To do so, it focuses on how AM enables deterministic control over hierarchical porosity, interfacial pathways, and multifunctional integration.

This review offers a thorough overview of the role of AM in advancing pore‐engineered materials for AWH, ISE, and RC. Figure 1 provides a schematic summary of the key transfer processes and pore‐structure requirements across these three technologies. Figure 2 complements this conceptual overview by presenting a technology development timeline that traces major milestones in pore‐engineered materials and highlights the growing influence of additive manufacturing in the field. First, the review examines how pore characteristics influence sorption thermodynamics, water transfer, vapor diffusion, photothermal conversion, and radiative behavior. This defines the architectural requirements for each application. Next, it evaluates the capabilities and limitations of major AM techniques in producing these features. It also discusses emerging AM‐compatible material platforms and highlights demonstrations in which architectural control enabled significant performance improvements. Finally, we discuss key challenges. These challenges are related to characterization, scalability, multifunctional integration, and process optimization. We also outline future research directions. These research directions are where pore engineering, multi‐physics modeling, and advanced manufacturing can converge. This will enable next‐generation water and thermal management systems.

**FIGURE 1:**
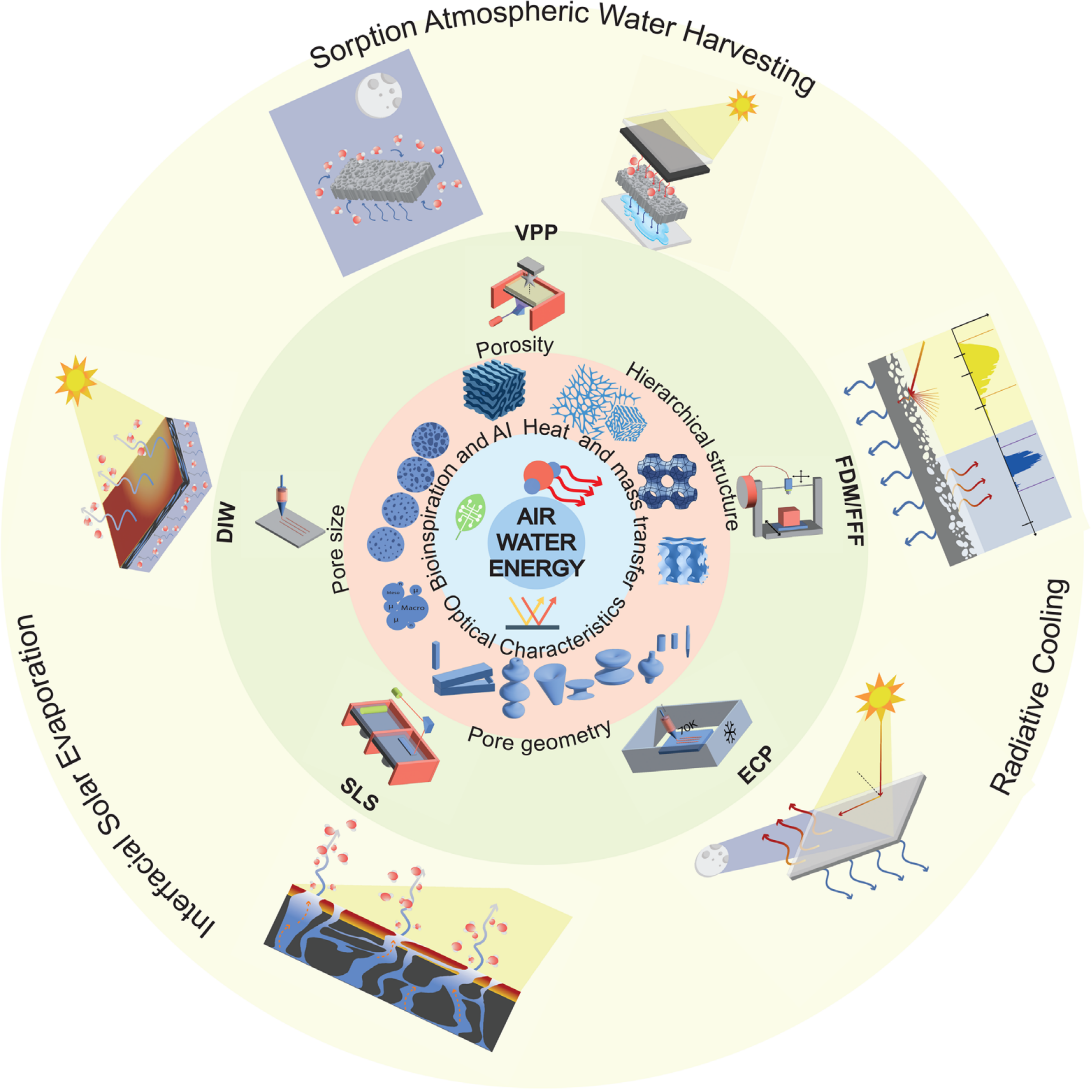
The processes that define the air–water–energy nexus depend on physical mechanisms (heat, mass, and light transfer), material inspirations, and pore architecture (size, connectivity, hierarchy), linking these properties to additive manufacturing strategies enables translation into real‐world systems.

**FIGURE 2:**
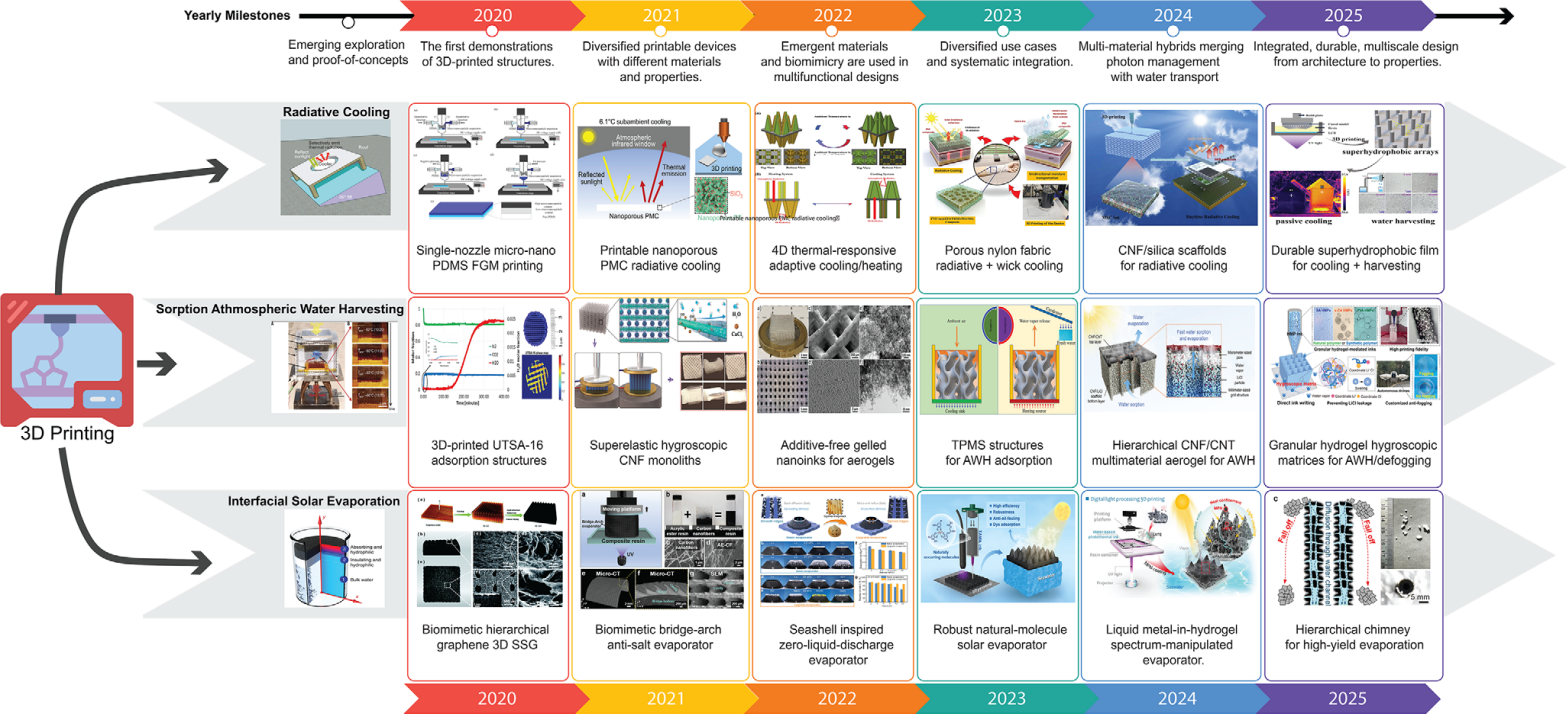
Development timeline of 3D‐printing technologies for radiative cooling, sorption atmospheric water harvesting, and interfacial solar evaporation: Key advances in AM strategies, material systems, and architected porous designs are presented chronologically alongside yearly milestones and representative achievements reported in the literature for each application domain. Reproduced with permission [30]. Copyright 2014, Springer Nature, reproduced with permission [31]. Copyright 2017, Science reproduced with permission [32]. Copyright 2014, Springer Nature reproduced with permission [33]. Copyright 2020, Wiley‐VCH reproduced with permission [34]. Copyright 2020, Elsevier reproduced with permission [35]. Copyright 2020, RSC Publishing reproduced with permission [36]. Copyright 2021, ACS Publications reproduced with permission [37]. Copyright 2021, ACS Publications reproduced with permission [38]. Copyright 2021, Wiley‐VCH reproduced with permission [39]. Copyright 2022, Wiley‐VCH reproduced with permission [40]. Copyright 2022, Wiley‐VCH reproduced with permission [41]. Copyright 2022, Elsevier reproduced with permission [42]. Copyright 2023, Elsevier reproduced with permission [43]. Copyright 2023, Elsevier reproduced with permission [44]. Copyright 2023, Elsevier reproduced with permission [45]. Copyright 2024, Elsevier reproduced with permission [46]. Copyright 2024, Wiley‐VCH reproduced with permission [47]. Copyright 2024, ACS Publications reproduced with permission [48]. Copyright 2025, ACS Publications reproduced with permission [49]. Copyright 2025, Wiley‐VCH reproduced with permission [50]. Copyright 2025, RSC Publishing.

## 2 Overview of 3D Printing Technologies for Water and Energy Applications

Within the scope of this review, AM is considered as a family of fabrication techniques distinguished by their resolution, material compatibility, and their expanding use cases. Its versatility is further enhanced by a continuously expanding range of compatible materials and techniques. Increasingly, 3D printing is being applied in novel ways within specialized fields, including AWH, RC, ISE, membrane distillation and catalysis [14, 51, 52, 53]. Its growing adoption in these domains is largely attributed to its unique ability to fabricate custom pore structures and complex monolithic and biomimetic architectures, features that are difficult to replicate using conventional manufacturing approaches. These capabilities make 3D printing a promising tool for addressing challenges in water and energy applications, where precise control over structural properties is often critical [54]. Figure 3 provides a graphical representation of the most widely utilized 3D printing technologies in the aforementioned fields: vat photopolymerization (VPP), selective laser sintering (SLS), direct ink writing (DIW), and extrusion‐based cryogenic printing (ECP). ECP, which shares similarities with DIW, has recently gained prominence for its capacity to define intricate pore geometries and macroscopic shapes [55, 56]. Additionally, fused deposition modeling (FDM) remains the most prevalent material extrusion 3D printing method, widely recognized for its accessibility and broad applicability [57]. Within the scope of the applications outlined, this review provides a concise overview of these 3D printing methods, detailing their features, advantages, and limitations in prototyping and producing complex geometries [58]. Particular emphasis is placed on how each technology facilitates the use of specific materials and enables precise control over pore architecture for applications in AWH, RC, and ISE.

**FIGURE 3:**
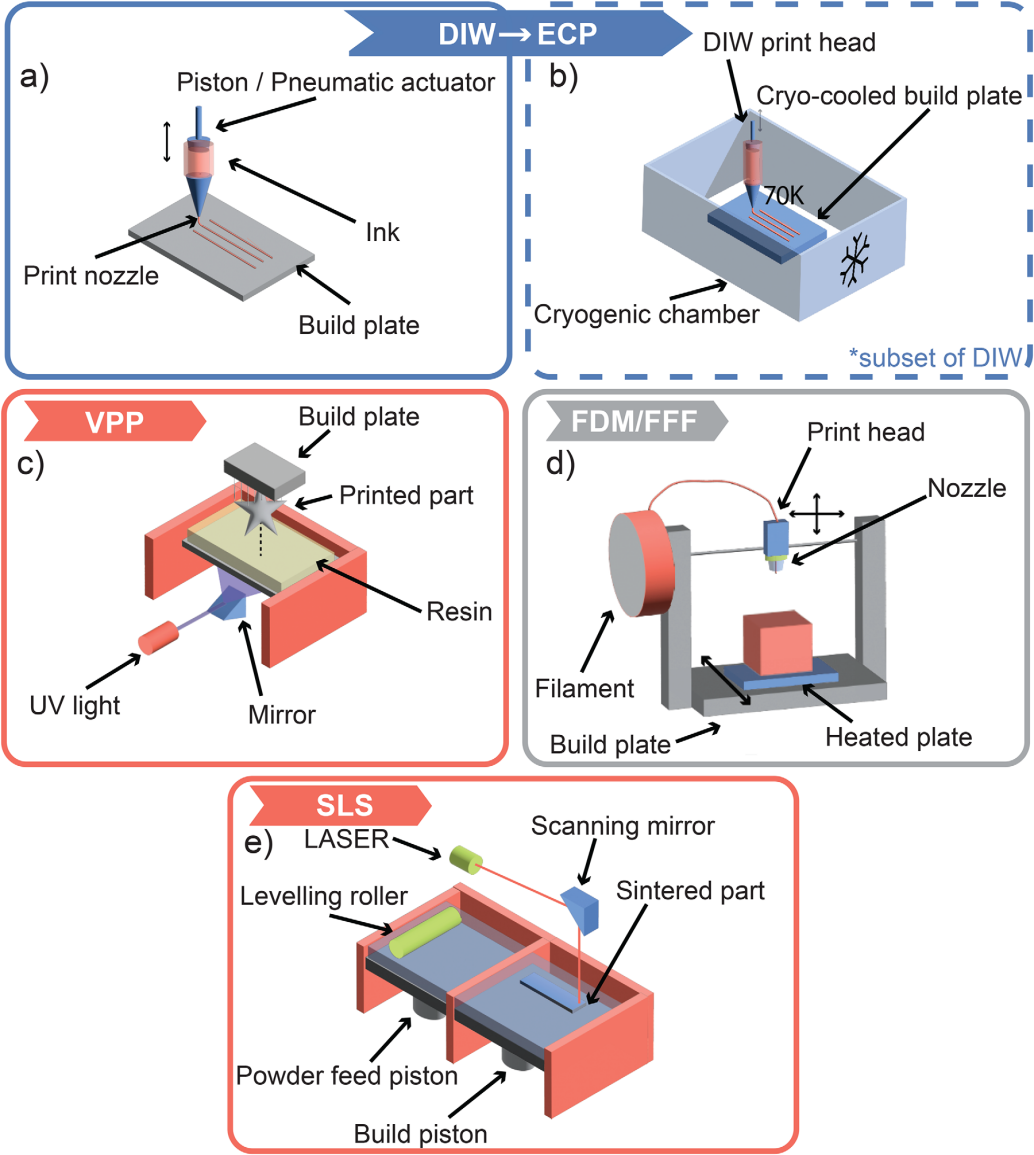
Overview of key 3D‐printing technologies for enabling material‐specific fabrication and precise pore architecture control for AWH, RC, ISE, and applications. (a) DIW, (b) ECP (subset of DIW), (c) VPP, (d) FDM, and (e) SLS.

### 2.1 Conceptual Logic of Pore‐Engineered Materials Enabled by Additive Manufacturing

This review adopts pore architecture as a central organizing principle, using AM‐enabled control of porous structures to rationalize performance trends across air–water–energy technologies without conflating structural effects with bulk material chemistry. AM enables deliberate pore engineering across multiple length scales, as shown in Figure 4a, allowing direct control over key architectural variables (Figure 4c), including pore size and size distribution, shape, orientation, connectivity, tortuosity (Figure 6a), spatial gradients, and hierarchical organization, through a combination of printing physics, toolpath design, and post‐processing. These structural variables directly govern fundamental functional properties: vapor diffusion and sorption kinetics through accessible surface area and pore openness [12] (critical for AWH), capillary pressure, permeability, and liquid transfer continuity via pore alignment and connectivity [59] (central to ISE), effective thermal conductivity and heat transfer, which are governed by porosity and tortuosity, and optical scattering, solar reflectance, and mid‐infrared emissivity through pore size and refractive index contrast [13] (essential for RC). Pore engineering therefore acts as the primary mediator between material class and application performance, defining water uptake capacity, evaporation rate, heat transfer efficiency, and radiative heat exchange.

**FIGURE 4:**
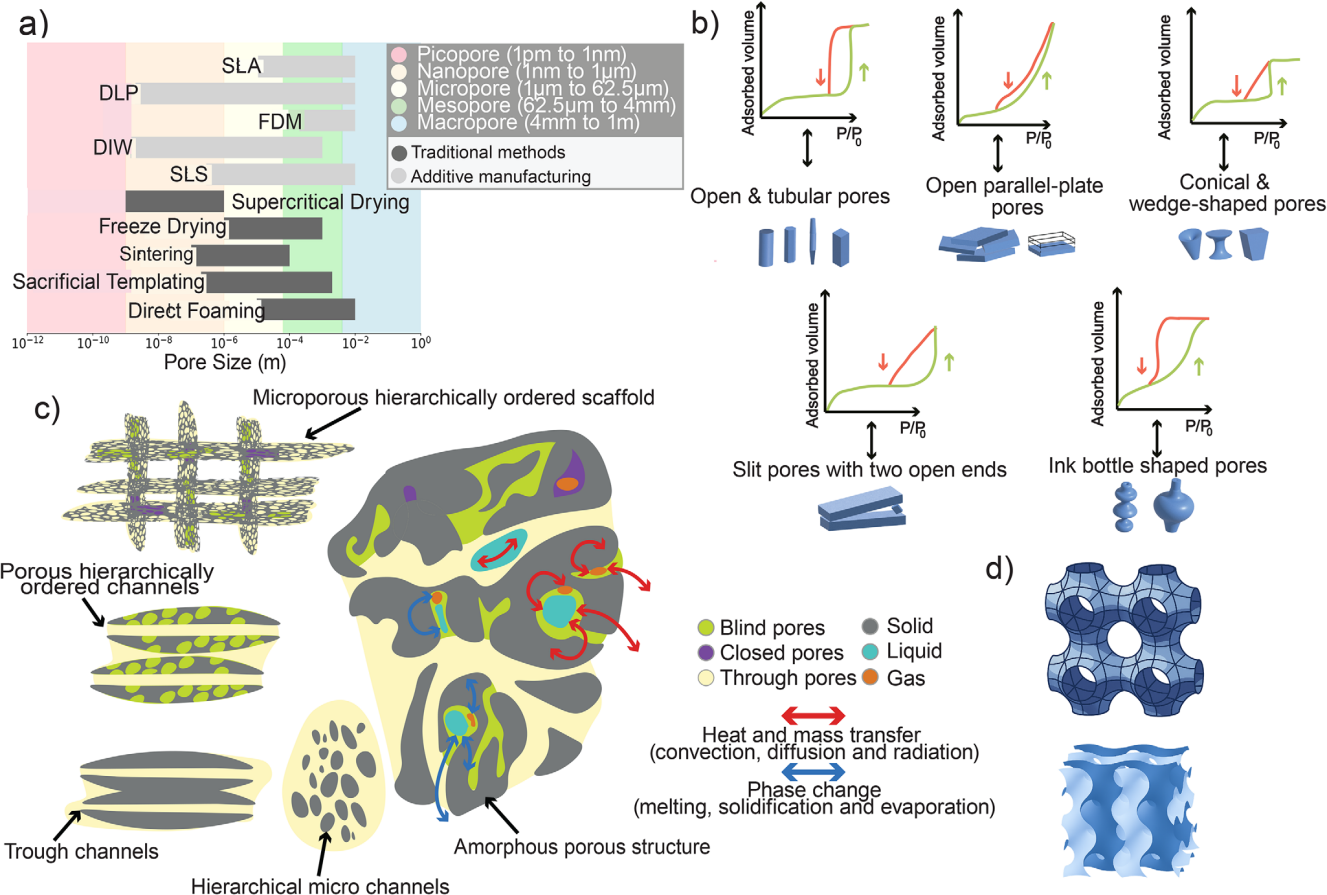
Pore geometry and pore‐level interactions: (a) Range of pore sizes achievable with different additive manufacturing techniques (data from Table 1) compared with traditional fabrication methods. (b) Schematic sorption–desorption isotherms illustrating the influence of pore shape. (c) Representation of pore architectures, pore types, and coupled heat and mass transfer interactions among liquid, gas, and solid phases. (d) Example of a triply periodic minimal surface (TPMS) hierarchical porous structure.

### 2.2 Principles of Direct Ink Writing (DIW)

Direct ink writing extrudes viscous inks such as polymers, ceramic foams, Pickering emulsions, hydrogels [60], aerogels, composites, or functional inks through a nozzle using pneumatic, screw, or piston actuators to build layered 3D structures, typically in ambient air or a gel suspension. This process provides the necessary resolution to accurately print intermediate products or precursors of materials such as hydrogels and aerogels, as noted above. DIW enables precise control over pore size, distribution, and hierarchical architecture [61]. It also supports multi‐material printing, enabling tailored properties like thermal conductivity, light absorption, and emissivity, which are vital for optimizing performance in water and energy systems. However, layering speeds remain relatively slow, limiting production of large or complex parts, and resolution is ultimately constrained by nozzle size. Post‐processing is often required to stabilize the printed structure and tune final pore morphology.

### 2.3 Principles of Extrusion‐Based Cryogenic Printing (ECP)

Extrusion‐based cryogenic printing (ECP) is a specialized variant of DIW that exploits ultra‐low‐temperature extrusion to generate hierarchical pore architectures. It is discussed here as a distinct process because of its unique pore‐forming mechanism, while acknowledging that it falls under the broader DIW family. Specifically, ECP utilizes cryogenically controlled extrusion whether through a cryogenically cooled nozzle, a sub‐zero build plate, or a fully cryogenic chamber to generate hierarchical pore architectures in a single step through in situ freezing and subsequent sublimation. By precisely tuning the freezing front and deposition parameters, ECP delivers tunable macro‐ and micro‐porosity, yielding composites with exceptionally low shrinkage, high mechanical strength, and finely controlled multiscale pore interconnectivity, features that underpin enhanced thermal conduction, mass transfer, and optical properties [62]. Despite its transformative potential, ECP remains constrained by a narrow palette of suitable inks (typically aqueous suspensions or gel‑based composites) and the need for specialized refrigeration hardware; nevertheless, post‑printing stabilization (e.g., freeze‑drying or gentle cross‑linking) is often minimal compared to traditional sintering or curing routes. Notably, this method yields low shrinkage rates and high mechanical robustness, making it particularly valuable for applications in bone tissue engineering and energy storage, where structural and functional integrity are critical [63].

Current research is increasingly focused on the development of hierarchically porous ceramics with enhanced damage tolerance, aiming to match or exceed the energy absorption characteristics of porous composites and metallic foams [64]. A recent advancement in multilateral cryogenic printing of soft hydrogels [62] has pioneered the composite printing of hydrogels for self‐sensing systems and soft robotics, with promising implications for SAWH.

### 2.4 Principles of Vat Photopolymerization (VPP)

Vat photopolymerization employs a laser or UV light projector as a light source to cure liquid polymer resin into solid layers. This technique offers high resolution, smooth surface finishes, and excels in producing intricate designs, making it ideal for prototyping high precision parts. It enables the creation of detailed pore structures with minimal additives and post‐processing, achieving the desired pore size and geometry, which is particularly advantageous for water related applications. However, a key limitation is the restricted range of materials, primarily polymers, or ceramic particles suspended in polymers compatible with photoinitiators, which may not suit all applications. Parts are often brittle in the green state and may require post‐processing to optimize mechanical stability. Based on the different curing methods, VPP is usually classified into stereolithography (SLA), digital light processing (DLP), two‐photon polymerization (TPP), and volumetric printing [65], where SLA and DLP are most widely used for precise pore engineering.

### 2.5 Principles of Fused Deposition Modeling (FDM)

Fused deposition modeling is an extrusion‐based additive manufacturing technique that constructs parts layer by layer by depositing thermoplastic filaments through a heated nozzle. As one of the most widely accessible and cost‐effective 3D printing methods, FDM supports a broad range of thermoplastic materials, making it an attractive option for both educational and industrial settings [66]. However, its relatively low resolution, visible layer lines and limited intrinsic microporosity restrict its use in precision porous systems, where controlled hierarchical pores are essential. Consequently, while valuable for initial design iterations, FDM is generally not the preferred method for manufacturing precision‐critical or hierarchically porous functional materials.

Nevertheless, ongoing research continues to explore FDM's utility in other functional areas, particularly by incorporating novel materials to create solar‐absorbing components. For instance, additives like graphene have been integrated to enhance solar absorption within thermoplastics. Hybrid approaches, such as combining granule‐based FDM with preceramic polymers, have also enabled the fabrication of robust silicon carbide (SiC) structures for high‐temperature applications like concentrated solar receivers [67]. Additionally, FDM has been used to produce optimized geometry solar concentrators that improve photocurrent generation [68]. These developments highlight an emerging role for FDM in the scalable manufacturing of certain solar‐enabled systems, even as its use in precision‐critical porous structures remains limited.

### 2.6 Principles of Selective Laser Sintering (SLS)

Selective laser sintering employs a laser to selectively sinter powder materials typically polymers [69], metals [70], or ceramics [71] into solid layers on a powder bed, eliminating the need for support structures. This technique excels in producing complex internal geometries, such as the porous architectures critical for applications discussed in this review. SLS enables the fabrication of functional prototypes and end‐use parts with robust structural integrity and tailored pore architectures, leveraging its broad range of available materials. However, its drawbacks include higher operational and unit costs, rough surface finishes requiring post‐processing, and lower resolution compared to VPP for fine details.

## 3 Engineering Pore Architecture and Hierarchy via Additive Manufacturing

### 3.1 The Critical Role of Pore Engineering in the Air–Water–Energy Nexus

Section 2 described the hardware, materials, and basic working principles of each 3D‐printing technology. The present section refocuses exclusively on how these techniques generate pores. That is, it discusses the physical mechanisms of pore formation. It also discusses the resulting pore geometries. And it discusses the architectural variables that can be engineered. These variables include size, connectivity, tortuosity, and hierarchy. To avoid duplication, we do not reiterate general descriptions of printer hardware or standard operational procedures. Instead, we analyze each AM method through the lens of pore‐scale physics, drawing on quantitative data from reported studies.

The performance of functional materials in AWH, RC, and ISE is fundamentally governed by the complex interplay of heat, mass, and light transfer within their porous architectures, where water transfer occurs through interconnected pore networks driven by capillary forces, pressure gradients, and fluid–solid interactions [72, p. 3]. As illustrated in Figure 4a, AM enables access to a substantially broader and more precisely tunable pore size range compared to conventional manufacturing techniques, allowing deliberate design of transfer‐relevant porosity across multiple length scales. These transfer processes, including vapor sorption/desorption, passive heat rejection, photothermal conversion, capillary‐driven flow, and radiative heat exchange, are predominantly dictated by pore network geometry rather than bulk material composition Key pore characteristics such as size, shape, connectivity, orientation, and hierarchical organization control transfer behavior across AWH, RC, and ISE systems. Pore size governs the dominant sorption mechanism, such as physisorption in microporous zeolites, while pore shape influences sorption–desorption hysteresis behavior (Figure 4b). Pore alignment, openness, and tortuosity regulate vapor permeability and effective thermal conductivity (Figure 4c), thereby influencing both mass transfer efficiency and heat transfer resistance. In RC applications, pore size and morphology further play a critical role in controlling optical scattering, solar reflectance, and mid‐infrared emissivity, where sub‐wavelength and hierarchical pore architectures enhance broadband solar reflection while promoting strong thermal emission within the atmospheric transparency window [13]. Hierarchical porous structures enable efficient multi‐scale heat and mass transfer while maintaining superior mechanical performance and surface accessibility [73, 74]. This interplay between pore geometry and hierarchical structure is pivotal in governing the fundamental forces that dictate water movement and thermal dynamics within porous media, including advanced triply periodic minimal surface (TPMS) geometries that combine high porosity with mechanical robustness and well‐defined transfer pathways [75]. Figure 4d presents a schematic illustration of a TPMS hierarchical porous structure, exemplifying the geometric complexity accessible through AM.

Conventional fabrication methods provide limited, indirect control over pore size, connectivity, and hierarchy, constraining the ability to tailor porous architectures to application‐specific transfer requirements. In contrast, additive manufacturing enables direct encoding of pore geometry into monolithic structures, allowing pore morphology to be treated as a controllable design variable rather than a stochastic outcome of processing. Recent AM‐based strategies for producing porous materials including emulsions [76, 77, 78, 79], sacrificial templates [80], microspheres [81, 82, 83], and foaming methods [51, 55, 84], have expanded the accessible design space and improved control over pore dimensions relative to earlier methods [52].

This section examines the pore architectures enabled by representative AM technologies and compares their achievable pore size ranges, spatial organization, and hierarchical features (as illustrated Figure 5 and systematically compared in Table 1), with emphasis on the implications of these architectural constraints for performance optimization in AWH, RC, and ISE systems.

**FIGURE 5:**
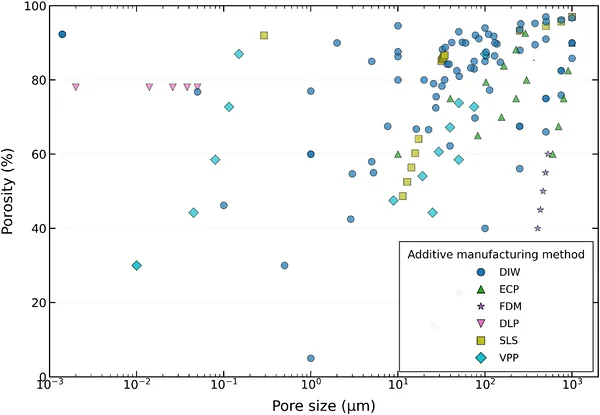
Data graph of the porosities and pore sizes of the 3D‐printed methods referenced in Table 1.

**TABLE 1:** Overview of 3D printing technologies with respect to their achievable porosity characteristics, pore size ranges, compatible material types, and required post‐processing methods.

3D‐Printing method	Porosity (%)	Pore size range	Key material(s)	Post processing	Key functional property	Refs.
SLS	92%–97%	Micropores: 0.29–38 µm Mesopores: 0.1–1 mm	SiC, SiO_2_ (AHS)	Not specified	Flexural strength of 40.93 MPa, good thermal stability	[81]
DIW	46.2%–85.8% (open)	Nanopores: ∼100‐200 nm Micropores: 5–50 µm Mesopores: 0.1–1 mm	SiOC, PMMA	Pyrolysis at 1000°C–1200°C	Compressive strength: 1.85–12.36	[80]
DLP	78% (nanopore vol.) 18% (macropore vol.)	Nanopores: 2–50 nm	Silica (OMS monolith)	Sol‐gel aging, calcination at 700°C	Thermally stable, low density (0.26 g/mL)	[101]
DIW	92.3%	Nanopores: 1.4 nm;	COF nanoparticles	Solvent exchange (2‐butanol to ethanol), supercritical CO_2_ drying	Not explicitly mentioned	[114]
DIW	86.3%–94%	Micropores and mesopores: 10–100 µm	Silica sols, hydrophobicity‐tuned particle surfaces	Sintering at 850°C or 950°C	Compressive strength: 1.32–3.38 MPa Thermal conductivity: 0.0635 W m^−^^1^ K^−^^1^	[119]
DIW	85% (open) 6% (closed, chiral structure walls)	Nanopores (from nanoemulsions, not quantified); Micropores and mesopores: 5–500 µm	Pickering nanoemulsions, micron‐sized polymer particles, zwitterionic surfactant	Drying, sintering at 850°C, chemical consolidation (optional)	Not explicitly mentioned	[120]
DIW	77%–85%	Mesopores: ∼100 µm Micropores: <10 µm	Ceramic hollow spheres, bentonite nanoparticles	Biocementation in a calcification bath (urea, calcium ions) for 2 days at ambient temperature	Compressive strength comparable to mineral foams sintered at 1000°C (specific values not provided)	[127]
DIW	87.6%–92.3% HA: 87.6%–87.7% HS: 88.9%–92.3%	Micropores and mesopores: 10–100 µm HA: 30–110 µm HS: 15–70 µm	Hollow microspheres (HA, HS)	Drying, sintering	Compressive strength: HA: 1.82–4.1 MPa HS: 2.4–2.65 MPa	[83]
FDM	Not explicitly quantified; interconnected porous structure implied by infill density (40%–60%)	Mesopores: 465 ± 63 µm	Polycaprolactone (PCL), hydroxyapatite (HA)	Not explicitly mentioned	Compressive modulus: 46.6–71.6 MPa	[128]
DIW	76.8%–83.1% (foam) 89.7% (lattice structure)	Macropores (not quantified) Mesopores: 41.5 – 137.4 µm Nanopores: 49.3–74.0 nm (micelle clusters)	α‐Alumina (α‐Al_2_O_3_), fumed silica (SiO_2_	Drying, sintering (650°C and 1500°C)	Compressive strength: 3.5–8.7 MPa Thermal insulation Lower thermal conductivity with higher porosity	[121]
DIW	90%–97%	Micropores and mesopores: 10–500 µm Micropores: <2 µm	Clay‐based wet foams	Drying, sintering (temperature‐tunable)	Compressive strength: 0.07–0.17 MPa Thermal conductivity: 37–44 mW/K m	[122]
SLS	48.7%–64.1% (open)	Micropores: 11.33–17.25 µm	Mullite; MnO_2_ (from Mn(Ac)2·4H_2_O and KMnO_4_ co‐precipitation); phenolic resin	Not explicitly detailed	Compressive strength: 0.8–22.1 MPa	[90]
SLS	85.1%– 86.7% (open)	Micropores: 31.5–34.5 µm	Mullite; fly ash hollow spheres (FAHSs); polyamide 12 (PA12)	Not explicitly detailed	Compressive strength: 1.4–2.08 MPa Thermal conductivity: 60 mW/K m	[91]
DIW	54.69%–70.02% (monoliths) 22.59%–34.65% (open) 35.03%–44.49% (closed) 68.28%–84.79% (lattices)	Mesopores: 97–150 µm; Micropores: 3–10 µm	Mullite, fly ash hollow microspheres (FAHMs)	Gelation, drying, sintering (600°C–1550°C)	Compressive strength: 3.02–42.9 Thermal conductivity: 295–1217 mW/K m	[104]
DIW	94.6%–96.7%	Macropores: mm‐scale Micropores: 10–50 µm	PCL, m‐HAp, m‐SiO_2_ nanoparticles	Solvent evaporation (24 h, RT)	Young's modulus: 42–516 kPa Compressive stress at 85% strain: 96–748 kPa	[124]
ECP	Exact porosity percentages not specified in the provided text, controlled by filament offsets and freeze‐drying process	Mesopores: 600–1000 µm Micropores: Not quantified	PCL	Lyophilization (freeze‐drying, 36 h)	Not explicitly mentioned	[110]
ECP	75%–92.6%	Mesopores: ∼290 µm Micropores: ∼40 µm Nanopores: tens of nm	Al_2_O_3_, chitosan	Freeze‐drying, sintering (1400°C)	Compressive strength: 8.854 MPa Thermal conductivity tested (TPS2500 equipment), no specific quantitative values provided	[64]
DIW	Surface porosity: Non‐porous (<5) Semi‐porous (5–40) Porous (>40)	Cellular pores (not quantified)	Graphene oxide (GO)	Freeze‐drying	Surface porosity tunable via shear stress and crosslinking	[117]
DIW	Mesh‐like macropores: BET surface area ∼794 m^2^/g but no porosity % given	Nanopores: ∼0.5	COF gels (SNW‐1, ATPG‐COF)	Refrigeration, drying, degassing	High surface area for adsorption	[115]
VPP	Not quantified (mesoporous)	Nanopores: 10–150 nm	Mesoporous silica, graphene, PEGDA	Sintering (1400°C, forming gas), spark plasma sintering (SPS).	Compressive strength: 29.5–57.9 MPa Electrical conductivity: up to 680 S/m. Thermal conductivity: 62–88 mW/K m Ink absorbs light across the visible and near‐UV spectrum due to graphene	[94]
DIW	Not quantified BET: 215–350 m^2^/g	Mesopores and macropores (mix)	Kevlar nanofibers (PPTA), DMSO	Supercritical CO_2_ drying or freeze‐drying; functionalization (hydrophobic, luminescent)	Tunable Poisson's ratio: −0.8 to 0.4; Energy absorption: 80.1 J/g Visible and UV luminescence achieved with dyes	[118]
DIW	Up to 90%	Hierarchical: 1–1000 µm	TCP, ZrO_2_‐based composites	Critical point drying, gold sputter‐coating	Compressive strength: 3.1 MPa Elastic modulus: 1.0 GPa	[125]
VPP	Hierarchically porous 30%–87%	Nanopores: 10–100 nm Micropores: 10–100 µm	ZrH_2_‐xerogel, photosensitive resin	Carbonization (Ar), boro/carbothermal reduction (vacuum)	Compressive strength: 0.23–16.8 MPa Thermal conductivity: 1510 mW/K m Sorbitol‐derived xerogel absorbance: 0.16 at 405 nm	[95]
DIW	66.6%–90.1% (open)	Micropores: 22.5–41.5 µm	TiO_2_ (coarse and fine powders)	Drying, sintering (1100°C)	Compressive strength: 1.1–18.0 MPa TiO_2_ noted for photocatalytic potential.	[116]
DIW	80%–90% (interconnected open)	Micropores and mesopores: 20–130 µm	Surface‐hydrophobized clay	Drying, sintering at 1000°C–1150°C, biocementation with *S. pasteurii*, UV sterilization.	Compressive strength: 0.9 MPa (dry) Biocementation, increased stiffness by 55%	[123]
VPP	Hierarchical porosity: 47.5%–73.8%	Micropores: 8.90–50 µm	SiC‐O_x_, HDDA resin, Co/Ni nitrates.	Debinding (60°C–400°C), partial sintering (1000°C)	Compressive strength: 4.10 MPa (73.8% porosity)	[78]
DIW	Hierarchical porosity: 58–93 vol%	Micropores: 5–50 µm Nanopores: unspecified	Alumina, hollow microspheres (HMs)	Binder burnout (∼540°C), sintering (1200°C)	Compressive strength: 1.02–5.0 MPa	[82]
ECP	60%–80% (HA) ∼72%–79.4% (PEEK/HA)	Mesopores: 260–300 µm Micropores: ∼10 µm Nanopores: ∼500 nm	HA, PEEK, chitosan	Freeze‐drying, sintering (200°C–1300°C)	Modulus: 117.7 MPa (HA), withstands 80% strain (PEEK/HA)	[109]

*Note*: The table further highlights the key functional properties of each technology in terms of mechanical, heat and mass transfer performance as well as optical characteristics.

### 3.2 Demonstration of AM Techniques for Pore Engineering

Across AWH, ISE, and RC, functional performance is governed less by bulk material chemistry than by the compatibility between material class, pore architecture, and manufacturing pathway, since each application imposes distinct transfer and optical requirements that arise from pore geometry and multi‐physics coupling rather than simply composition. Additive manufacturing enables purposeful control of porosity and pore network design across scales, which is critical for optimizing vapor diffusion, capillary transfer, and radiative exchange beyond the limits of conventional fabrication routes [85]. Although all three rely on porous media, the required pore functions differ fundamentally: sorption‐based AWH favors materials with accessible surface pores for vapor diffusion and internal pore volume for water retention (e.g., hydrogels, MOFs/COFs, polymeric or carbon networks) [86], whereas ISE prioritizes through‐thickness, ordered channels that sustain capillary‐driven water delivery to the evaporation interface, typically realized in ceramics, polymers, or fibrous networks coupled to photothermal absorbers [59]. RC places minimal emphasis on fluid transfer and instead depends on optically engineered porosity, often achieved through polymer matrices incorporating hollow silica spheres or dielectric nanoparticles to promote solar scattering and mid‐infrared emission [19].

These material requirements impose distinct compatibility constraints on AM techniques. Powder‐bed laser processes such as SLS [87] naturally accommodate ceramics, oxides, metals, and porogen‐rich composites that can tolerate high thermal loads, whereas polymers and hydrogels are fundamentally incompatible with such thermal energy fluxes. VPP is well suited for polymers and hydrogels but requires photocurable chemistry, relegating ceramics to resin‐templated routes [88]. FDM is constrained to thermoplastics within narrow viscosity windows and relies on additives or leaching strategies to introduce secondary porosity. In contrast, DIW and its freeze‐assisted variant, ECP, exhibit the broadest material compatibility, accommodating polymers, ceramics, carbons, composites, and hybrid sorbents, albeit at the cost of extensive post‐processing to achieve final microstructure and properties including hierarchical pore networks and tailored carbon architectures [56].

From a structural standpoint, pores produced by AM can be classified into three distinct origins: inherent pores that arise directly from the printing physics, designed pores encoded through toolpath or geometry, and post‐processed pores generated independently of printer resolution. Inherent porosity includes interparticle voids in SLS, interlayer gaps in FDM, ink‐derived micro voids in DIW, and freeze‐cast lamellae in ECP. Designed porosity encompasses lattices, channels, gradients, and TPMS architectures explicitly defined in CAD and accessible across most extrusion‐ and light‐based techniques [71]. Post‐processed porosity such as that produced by porogen burnout, phase separation, foaming, or freeze‐drying, dominates when hierarchical or nanoscale features are required. Post‐processing thus acts as a primary design lever rather than an auxiliary step, enabling pore generation, preservation of fragile architectures, mechanical consolidation (e.g., sintering, SPS, calcination), and chemical activation. Importantly, AM techniques differ in their reliance on these steps: SLS and VPP depend on post‐processing to define functional pore networks, DIW and ECP primarily use it to stabilize multiscale architectures, and FDM employs it selectively for secondary porosity. Together, AM and post‐processing define a coupled design space that ultimately governs material performance across the air–water–energy nexus [89].

#### 3.2.1 Tailoring Pores With SLS

SLS enables the fabrication of porous structures with a broad and tunable porosity range, spanning approximately 49%–97% depending on powder system design and post‐processing strategy [81, 90, 91]. Achievable pore sizes extend from the microscale (0.29–38 µm) to the mesoscale (0.1–1 mm), allowing both fine and coarse porous networks to be realized within mechanically coherent monoliths. Pore‐scale features are predominantly governed by powder formulation and sintering behavior.

Highly porous SLS architectures are most commonly achieved through the incorporation of sacrificial pore‐forming agents, with porosity generated during post‐processing as the porogens are removed and the remaining framework consolidated. Hollow spherical porogens such as SiO_2_ microspheres (AHS) [81] or fly ash (FAHSs) [91] have been widely used to produce porosities exceeding 85%, with porosity generated after porogen removal during post‐processing.

In [81], SiC‐based systems incorporating AHS achieve porosities of 92%–97% with hierarchical pore distributions from micro‐ to mesopores, while maintaining high flexural strength (40.93 MPa) due to effective stress redistribution within the sintered network. Similarly, FAHS‐based mullite systems exhibit porosities of 85.1%–86.7% with larger micropores (31.5–34.5 µm), yielding moderate compressive strengths of 1.40–2.08 MPa [91]. Such structures are well suited for applications prioritizing thermal insulation or high surface area, including sorbent scaffolds for AWH [46] and insulating components for RC systems [92].

SLS can also be configured to favor mechanical robustness through the use of dense composite powder systems. Double‐shell mullite powders with MnO_2_/phenolic resin coatings [90] demonstrated lower porosity (48.7%–64.1%) and narrower micropore distributions (11.33–17.25 µm), but provide substantially enhanced mechanical performance, with compressive strengths ranging from 0.8 MPa to 22.1 MPa and improved fracture toughness. These attributes make SLS‐fabricated components based on dense or composite powder systems well suited for load‐bearing roles within functional devices, where structural integrity must be maintained alongside geometric complexity. Overall, SLS offers a unique combination of geometric freedom and mechanical continuity, while pore hierarchy and transfer‐relevant features are predominantly dictated by pore‐forming strategies [91] and sintering conditions. Common limitations include rough surface finish, higher processing cost, and limited quantitative reporting of pore interconnectivity, which is often inferred rather than directly measured [93]. As detailed in Table 1, SLS is a powerful tool for fabricating complex, self‐supported porous components where a combination of good mechanical properties and tailored porosity is required.

#### 3.2.2 Tailoring Pores With VPP

VPP, including SLA, DLP, and two‐photon polymerization (TPP), offers exceptional control over pore architecture across multiple length scales, spanning nanopores (0.01–150 nm) to micropores (8.9–100 µm), with porosity ranging from approximately 30% to 87% [78, 94, 95]. This multiscale control makes VPP particularly relevant for applications in the air–water–energy nexus, where simultaneous regulation of vapor sorption, capillary transfer, and thermal behavior is essential, as vapor sorption in hygroscopic porous materials is directly coupled with latent heat release and heat transfer, while vapor permeability influences both mass and heat transfer processes [96]. A defining feature of VPP is the decoupling of bulk geometry from pore morphology: nanoscale pores can be generated via TPP, while microscale pores are accessible through SLA and DLP, enabling hierarchical architectures that combine tailored internal porosity with complex external form [97].

Although high‐resolution printing can limit throughput [98, 99], templating strategies such as emulsion‐based printing, phase separation, and sacrificial fillers enable the formation of interconnected micro‐ and mesoporous networks while maintaining practical print speeds and mechanical integrity [78, 100]. Material functionality in VPP can be further expanded through the incorporation of conductive or photothermal fillers, including graphene, SiC, and metal oxides, enabling coupled mass and energy transfer relevant for solar‐driven evaporation and electromagnetic wave absorption [78, 94, 95]. Notably, photocurable templating approaches have enabled the fabrication of ordered mesoporous silica (OMS) monoliths with high nanopore volume (78%) and tunable pore diameters between 2 nm and 50 nm, providing well‐defined vapor diffusion pathways [101]. Additional use of sacrificial templates such as PMMA yields open porosities of approximately 50% while preserving sufficient mechanical robustness for operation under cyclic wetting and thermal loading [100].

Across the printing methods in VPP, post‐processing is a critical factor that determines the final morphology of the pores. Techniques such as partial sintering [78], spark plasma sintering [94], carbothermal reduction [102], and controlled calcination [101] determine the balance between porosity, interconnectivity, and mechanical stability, directly influencing permeability, thermal conductivity, and durability. While excessive densification can suppress pore connectivity [100], optimized post‐processing preserves hierarchical heat and mass transfer pathways essential for AWH, RC, and ISE [103]. Consequently, the primary advantage of VPP lies not in maximum mechanical strength, which can be comparable to DIW [104], but in its unparalleled ability to engineer multiscale, highly connected pore architectures with application‐specific transfer properties.

#### 3.2.3 Tailoring Pores With FDM

FDM enables the fabrication of porous scaffolds with moderate and largely geometry‐driven porosities, typically inferred from infill densities of ∼40%–60% and mesopore sizes around 465 ± 63 µm. Low porosities can be achieved by filament additives such as salt‐in filaments that get their porosity from later salt leaching [105], dissolvable porogen filament composites such as [106] where they employed polycaprolactone (PCL) as the primary polymer ceramic filler, and poly(ethylene glycol) (PEG) as a porogen, where the porogen was removed by leaching achieving porosities up to 75% (pore sizes: 20–700 µm) and polymer blend composites like the one demonstrated in Ref. [107] where a poly (vinyl alcohol) (PVA) and poly (lactic acid) (PLA) blend was subjected to gas foaming to create micropores with pore sizes smaller than 10 µm. PCL/HA systems can generate interconnected mesopores primarily governed by toolpath design and nozzle diameter [84]. Adjusting parameters such as filling rates further allows systematic tuning of pore size from tens to nearly 1000 µm in n‐HA/PA66 scaffolds [108], demonstrating the sensitivity of pore architecture to printing parameters.

Techniques such as freeze‐drying [84], particulate leaching [106], and gas foaming [107] are mostly used in FDM porous structures for the creation of secondary pore networks that enhance connectivity but increase structural complexity. Overall, FDM provides reliable control over meso‐ to macroscale porosity through geometric design but lacks intrinsic hierarchical pore control, limiting its suitability compared to DIW or VPP for applications requiring tightly coupled heat and mass transfer.

#### 3.2.4 Tailoring Pores With ECP

ECP produces bioinspired porous structures with controlled porosity ranges of 60%–92.6% and pore sizes spanning nanopores (tens of nm to 500 nm) to mesopores (260–1000 µm), making it attractive for damage‐tolerant porous scaffolds [62, 109] and bone tissue engineering [110]. The resulting pore networks exhibit low tortuosity and high interconnectivity, facilitating capillary‐driven liquid transfer and vapor diffusion [109]. Materials processed via ECP typically require freeze‐drying to preserve these delicate architectures. For example, Al_2_O_3_ structures fabricated by ECP and freeze‐dried at −60°C exhibit highly open pore networks with porosity values as high as 92.6% [62], while HA/PEEK systems achieve porosities in the range of 60%–80% with interconnected channels suitable for fluid transfer [109]. Similarly, PCL‐based scaffolds produced using ECP display hierarchical pore architectures spanning macro‐ and microscale features, although quantitative porosity values are not consistently reported [110]. Mesopores (260–1000 µm) provide continuous and low‐resistance transport pathways that facilitate vapor diffusion and liquid flow, while smaller micro‐ and nanopores (10–40 µm and below) increase specific surface area and solid–fluid interfacial contact, thereby enhancing vapor sorption capacity, capillary pressure, and interfacial heat exchange through increased adsorption enthalpy and reduced characteristic transfer lengths [111].

Compared to DIW, ECP offers a narrower but more controlled porosity window (60%–92.6% vs. 23%–97%) with reduced pore shrinkage, which is advantageous for applications requiring stable, highly porous sorption media. However, the lack of quantitative porosity and pore size data for PCL scaffolds limits direct comparison across studies and underscores the need for standardized porosity characterization. ECP enables the fabrication of well‐defined, multiscale, bioinspired porous architectures via freeze‐casting, yielding hierarchical pore networks with high interconnectivity [112]. Such architectures promote efficient mass transfer by providing continuous, low‐tortuosity pathways for vapor diffusion and capillary‐driven fluid flow, while simultaneously supporting efficient heat transfer through enhanced solid–fluid interfacial area and reduced heat transfer length scales [113]. The resulting coupling of heat and mass transfer is particularly relevant for evaporation and sorption‐driven processes, and can be further tuned through integration with DIW‐derived pore modifiers to adjust pore size distribution and connectivity.

#### 3.2.5 Tailoring Pores With DIW

DIW stands as the most versatile additive manufacturing technique for porous materials, achieving an exceptional porosity range of 23%–97% with pore architectures spanning from molecular‐scale nanopores (0.5 nm) to millimeter‐scale macropores, enabling precisely tailored structures for applications across the air–water–energy nexus. DIW enables the fabrication of hierarchically porous structures ideally suited for AWH [49] and gas capture [32]. The technique produces nanopores (0.5–1.4 nm) in COF structures with high BET surface areas (∼794 m^2^/g), optimized for strong vapor adsorption [114, 115], while simultaneously creating meso‐ and macropores (5–1000 µm) that facilitate rapid vapor transfer to adsorption sites. This architecture enables simultaneous high uptake capacity and rapid vapor transfer in AWH systems. Graphene‐based aerogels with tunable surface porosity (<5% to >40%) further demonstrate DIW's capability to create optimized sorbent materials These architectures combine large water‐transfer channels (0.5–1 mm macropores) with extensive evaporative surface area (10–500 µm micropores), enabling efficient capillary water delivery and vapor escape. Compatibility with photothermal materials such as TiO_2_ [116] and graphene [117] enables light‐absorbing evaporative structures with tailored thermal and wetting properties.

While less explored for RC, DIW's capability to produce materials with controlled porosity across multiple length scales presents opportunities for designing spectrally selective surfaces [36]. The technique can create structures with specific pore size distributions that enhance solar reflectance while maintaining high thermal emissivity in the atmospheric window. The auxetic behavior achievable through DIW of Kevlar aerogels (Poisson's ratio −0.8 to 0.4) further suggests potential for developing mechanically adaptive cooling materials [118].

Post‐processing choices critically influence final porosity and connectivity: supercritical CO_2_ drying and freeze‐drying preserve delicate nanoporous networks for adsorption applications, while sintering can reduce pore accessibility. DIW's exceptional material versatility encompasses ceramics (SiOC [80], silica [119, 120], alumina [82, 121], TiO_2_ [116], clay [122, 123], mullite [104]), polymers (PCL [124]), composites (TCP/ZrO_2_ [125], mullite/FAHMs [104]), COFs [110, 114], graphene [117], Kevlar aerogels [118], and biominerals [126]. This material selection enables the design of application‐specific architectures, such as bioactive surfaces for sorption‐based processes and surfaces with tailored hydrophobic or hydrophilic properties for specific types of atmospheric water harvesting, including sorption‐based AWH, heat‐pump‐assisted AWH, fog harvesting, and dew collection.

### 3.3 Discussion: Translating Pore Design to Advanced Functional Materials

Based on the data presented in Figure 5, clear trade‐offs emerge in selecting AM strategies for water‐ and energy‐related applications, with pore architecture functionality rather than printing resolution serving as the primary differentiator. A central challenge across all systems is balancing high porosity, which enhances vapor uptake, mass transfer, and interfacial heat and energy exchange, with sufficient mechanical integrity for long‐term operation [17, 19, 59, 86]. Techniques such as DIW and ECP printing are particularly effective at producing hierarchical, highly interconnected pore networks spanning micro‐ to macroscales, making them well suited for SAWH where rapid vapor diffusion and efficient liquid transfer are essential. In contrast, RC places stricter demands on optical and thermal properties than on mass transfer, favoring approaches such as VPP and DIW that enable precise control over nanoscale to microscale features and closed or semi‐closed pore morphologies required for spectral selectivity and thermal insulation [92]. ISE occupies an intermediate design space, benefiting from AM techniques capable of generating open, continuous macroporous networks that sustain capillary water delivery and unobstructed vapor escape, most effectively achieved through DIW‐, ECP‐, or geometry‐driven FDM‐based architectures. Collectively, these trends underscore that the optimal AM technique is dictated not by the process itself, but by the pore architecture and transfer functionality required by the target application.

## 4 Heat and Mass Transfer and Optical Properties of 3D‐Printed Porous Media

### 4.1 Heat and Mass Transfer Mechanisms and Optical Characterization in Engineered Pores

In the design of systems for AWH, RC, and ISE, performance is governed not only by material chemistry but by how pore architecture regulates coupled heat, mass, and radiative transfer. Figure 6a performance is governed not only by material chemistry but by how pore architecture regulates coupled heat, mass, and radiative transfer. In porous media, pore size, connectivity, tortuosity, and hierarchy define effective thermal conductivity, permeability, sorption kinetics, and optical response [129, 130] and are main functions that should be derived by the AM process. Hierarchical pore architectures provide advantages over stochastic porosity by enabling spatial separation of functions [131]. Macropores reduce pressure drop and facilitate vapor penetration, micropores regulate capillary transfer and liquid distribution, while nanopores govern surface wetting, sorption energetics, and light scattering [132]. Such ordered gradients enable controlled heat and mass fluxes rather than uncontrolled coupling, improving predictability and system‐level efficiency [133]. In ISE systems, for example, two‐phase liquid transfer can be stabilized through vertically aligned or graded channels that minimize hydraulic resistance while maintaining continuous water replenishment at the evaporation interface [72, 134].

**FIGURE 6:**
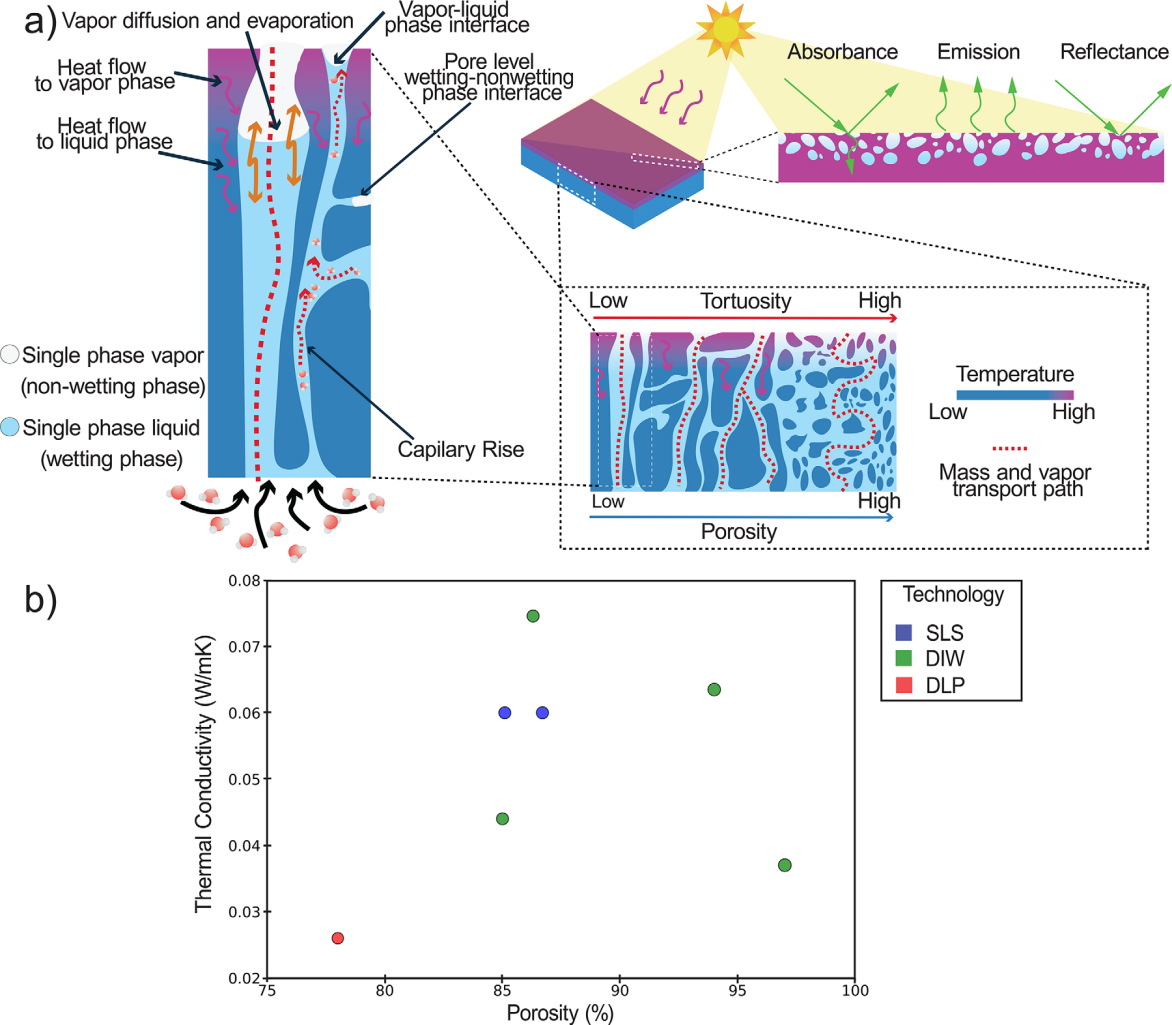
(a) Schematic representation of the fundamental mechanisms governing pore‐level heat and mass transfer, as well as the associated optical interactions within porous 3D‐printed structures. (b) Thermal conductivity as a function of porosity for different 3D‐printing technologies, as summarized in Table 1.

Quantifying the coupled interactions between porous media, fluid dynamics, and heat transfer remains challenging, particularly because porosity simultaneously alters permeability, effective thermal conductivity, radiative exchange (in case of RC), and local phase‐change dynamics [135]. This complexity has motivated the development of advanced modeling approaches that attempt to resolve multi‐physics transfer across pore scales. Kumar et al. [136] introduced a modified zonal method to analyze combined conduction and radiation in macroporous media demonstrating that at high porosities radiative heat transfer between internal surfaces can become comparable to solid conduction. Such effects are especially relevant in AWH and ISE systems, where temperature gradients drive sorption–desorption or evaporation and where excessive internal radiation may either enhance regeneration or induce parasitic heat losses depending on architecture. Similarly, Díaz‐Marín et al. [137] developed a detailed model for sorption–desorption kinetics and liquid–vapor distributions in hygroscopic hydrogels, an emerging material platform for AWH, showing that pore size distribution and connectivity govern vapor penetration depth, sorption front propagation, and regeneration uniformity. These transfer frameworks are directly translatable to AM architectures, where deterministic control over channel alignment, graded porosity, and tortuosity enables transfer pathways to be designed a priori or inferred post‐fabrication by post‐processing. Translating such insights into material design has driven interest in porous architectures with tailored thermal conductivity, such as metal foams that leverage continuous solid networks to enhance heat spreading in thermally limited regimes [138]. However, porosity plays fundamentally different roles across applications. In AWH, high interconnected porosity reduces diffusion resistance and accelerates sorption kinetics, yet excessive macroporosity lowers volumetric sorbent density and overall water uptake capacity [139]. In ISE, hierarchical porosity sustains capillary replenishment and vapor escape, but overly large or poorly distributed pores can destabilize thin liquid films and increase conductive losses to bulk water [132]. In RC systems, increasing porosity suppresses solid conduction and lowers effective thermal conductivity, but random or optically mismatched pore distributions may enhance solar penetration and internal absorption, offsetting radiative cooling gains [132, 139]. Thus, optimal porosity is not simply “maximized,” but application‐specific and architecture‐dependent, requiring coupled control of permeability, heat transfer, and optical response.

Despite increasing modeling attention to heat and mass transfer, optical transfer remains comparatively underexplored [140]. This gap is particularly pronounced for light absorbance, reflectance, emission, and scattering in porous media, despite their central role in photothermal conversion and spectral selectivity. Recent studies have investigated MOF‐based materials, organic polymers [141] and graphene oxide composites, for applications such as desalination [142], anti‐icing/deicing [143], and ISE [144], yet most lack rigorous simulations or mechanistic analysis of light dispersion and photonic behavior, especially in 3D‐printed hierarchical structures [145]. In the following sections, we therefore examine the optical characteristics of additively manufactured porous materials, building on the fabrication strategies summarized in Table 1 to address this critical gap.

### 4.2 Optical, Heat, and Mass Transfer Properties Performance by AM Technology

The selection of an AM technique dictates achievable architectural resolution, porosity range, material compatibility, and post‐processing pathways, thereby indirectly defining transfer and optical properties. Performance, however, is not intrinsic to the AM method itself, but to the pore hierarchy and morphology that each technique enables. This architecture‐driven paradigm is clearly demonstrated in high‐performance passive daytime radiative cooling systems, such as hierarchically porous polymer coatings [20], hierarchical‐morphology metafabrics [146], and hierarchically structured cooling ceramics [147]. Although not fabricated via AM, these systems share a unifying structural principle: multiscale pores spanning nano‐ to microscale dimensions maximize broadband Mie scattering across the solar spectrum (UV–vis–NIR), achieving solar reflectivity exceeding 95%, while preserving high mid‐infrared emissivity (>90%) within the 8–13 µm atmospheric window. The governing factor is hierarchical pore engineering rather than material chemistry alone. Accordingly, hierarchical porosity should be treated as a unifying design parameter governing optical, heat, and mass transfer behavior across AM‐fabricated systems.

#### 4.2.1 Heat and Mass Transfer Performance is Governed by Porosity and Hierarchy

Porosity remains the primary parameter governing effective thermal conductivity and permeability, with AM enabling a wide range (∼23%–97%) across SLS, VPP, DIW, ECP, and FDM [64, 81, 91, 114, 119]. As shown in Figure 6b, effective thermal conductivity does not scale monotonically with porosity alone, but is strongly governed by the architecture of the solid framework, particularly the connectivity of the network and the thickness of the struts or pore walls. As a result, structures with similar porosities may exhibit markedly different thermal conductivities depending on whether heat is transferred through continuous, dense pathways or through thin, tortuous, and weakly connected networks. Consequently, accurate control over heat transfer requires consideration not only of porosity, but also of architectural characteristics such as nodal connectivity, strut geometry, and hierarchical organization. For instance, ultra‑low thermal conductivity (<0.1 W m^−1^ K^−1^) in DIW‑printed Pickering foams [119] and VPP‑fabricated mesoporous composites [78] arises not merely from high porosity but from thin, phonon‑scattering strut geometries and limited solid‑phase connectivity. Conversely, sintered DIW mullite structures [104] achieve higher thermal conductivity (295–1217 mW m^−1^ K^−1^) due to dense, interconnected strut networks, despite similar porosity levels. These contrasts underscore that strut thickness and connectivity dominate thermal behavior. For heat transfer, increasing porosity generally reduces solid‐phase conduction while enhancing radiative exchange and, in open‐cell structures, convective contributions at high void fractions. Ultra‐low thermal conductivity values (<0.1 W m^−1^ K^−1^) reported in SLS and VPP [76, 78], Pickering or wet foams produced via DIW [119], and mesoporous composites fabricated with VPP arise from a combination of thin strut geometry, high void fraction, and multiscale pore distribution. Phonon scattering at nanoscale strut boundaries, limited conduction pathways, and trapped air in hierarchical pores suppress solid heat transfer, while macropores and open‐cell channels introduce convective contributions that remain minor under ambient conditions. Conversely, enhanced thermal conductivity, necessary in heat‐deficient ISE systems, is achieved through high‐conductivity materials (e.g., ZrB_2_ in VPP [95]) or by creating dense networks via high‐temperature sintering in DIW [104]. These conductive architectures distribute photothermal energy efficiently to the interface but introduce trade‐offs: increased density or closed porosity improves heat transfer but can hinder vapor diffusion and capillary‐driven replenishment.

For mass transfer, hierarchical and interconnected pore networks are critical. Macro‐ and mesopores provide low‐resistance pathways for vapor and liquid transfer, while micropores and nanostructures facilitate capillary action and fluid retention. Such architectures, engineered via bioinspired freeze‐casting in ECP [109], photopolymerization‐induced phase separation (PIPS) and partial sintering and phase separation in VPP [78, 94, 148], emulsion and HIPE templating in DIW [120] and porogen leaching in FDM [84, 149], minimize tortuosity and promote uniform distribution. The result is optimized sorption–desorption in AWH and sustained evaporation in ISE, where continuous replenishment and controlled vapor escape prevent localized dry‐out or salt accumulation. These examples highlight that hierarchical porosity and connectivity, rather than bulk porosity alone, are the governing design variables for achieving high‐performance coupled heat and mass transfer in AM‐fabricated porous media.

#### 4.2.2 A Critical Gap Exists in the Quantification of Optical Properties

While the reviewed AM techniques clearly enable control over porosity‐driven heat and mass transfer, a significant and consistent deficit persists in the characterization and simulation of optical properties across SLS, VPP, DLP, FDM, ECP, and DIW platforms [140]. The high surface area and hierarchical porosity intrinsic to these structures suggest strong potential for light management via scattering, absorption, or tailored emissivity for photothermal conversion, RC, and solar‐driven ISE [141, 142, 144]. However, quantitative data on full‐spectrum reflectance, transmittance, absorptivity, and mid‐infrared emissivity remain exceedingly rare. Functional outputs such as evaporation rate or cooling power are typically reported without correlating them to measured spectral optical properties [134].

In ISE systems, pore geometry governs solar absorption through multiple scattering and light trapping; deeper or more tortuous pores increase optical path length but may distribute heat away from the air–water interface, reducing interfacial heating efficiency. Crucially, optical optimization must be coupled with appropriate fluidic architecture: hierarchical macrochannels and capillary‐active microstructures are required to continuously supply liquid to the photothermal surface and prevent dry‐out [141, 142]. Thus, efficient ISE performance depends on the coordinated design of optical confinement and liquid transfer rather than absorption alone. Similarly, in SAWH systems, high porosity must be complemented by hierarchically organized transport pathways that enable rapid vapor diffusion during adsorption and efficient desorption during regeneration. While sorption capacity is frequently quantified, the role of macro‐ and mesopore alignment in accelerating desorption kinetics and reducing vapor transfer resistance is rarely analyzed within AM‐defined architectures. Optical potential is often inferred from morphology—for example in SLS mesoporous scaffolds or ECP‐fabricated Al_2_O_3_ structures [64, 109], but not experimentally quantified. Even where DIW produces very high surface area structures [92] or VPP/DLP offer nanoscale feature control, these are seldom linked to measured photonic performance. Thus, the primary deficit is not the absence of heat and mass transfer studies, but the lack of integrated multi‐physics characterization that couples radiative, thermal, and fluid transfer within the same AM architecture. Without concurrent optical quantification and transfer modeling, rational decoupling of heat, mass, and light management remains unattainable [11]. Establishing predictive structure–property–spectrum correlations therefore represents a critical next step for AM‐enabled porous media in AWH, RC, and ISE systems. This gap is not limited to optical characterization. Research on AM‐enabled AWH, ISE, and RC systems rarely provides quantifiable data on how printing parameters, such as nozzle size, layer height, curing conditions, freeze‐drying protocols, and sintering profiles, translate into measurable pore‐architecture variables. These variables include pore size distribution, connectivity, tortuosity, and anisotropy. As a result, the connection between AM processing, the resulting microstructure, and the functional transfer properties, such as effective diffusivity, permeability, sorption/desorption kinetics, evaporation flux, and net cooling power, is frequently presumed rather than explicitly demonstrated. In the following application‐specific sections (5.1–5.3), we extract and compare these structure–process–performance relationships where quantitative data are available, while Tables S4–S6 summarize the correlations between AM processing parameters and resulting functional material properties.

### 4.3 Conclusion and Future Perspective

AM provides unparalleled design freedom to decouple and tailor the thermal, mass transfer, and optical properties of porous media by engineering their architecture across multiple scales. The scientific consensus indicates that hierarchical porosity is key to enhancing mass transfer and achieving extreme thermal conductivities, while material selection and post‐processing fine‐tune these properties [113, 132]. To fully realize multifunctional systems for the air–water–energy nexus, future work must move beyond demonstrating architectural feasibility. Research should prioritize the integrated quantitative characterization of transfer and optical properties within the same structures and develop advanced multi‐physics modeling that can simulate the interplay of heat, mass, and light within these complex 3D‐printed geometries [150]. Bridging this gap will enable the rational design of next‐generation porous media for high‐efficiency AWH, RC, and ISE applications.

## 5 Technology‐Specific Reviews

### 5.1 3D Printing of Hygroscopic Sorbents for Atmospheric Water Harvesting

#### 5.1.1 Introduction to SAWH

SAWH operates through five cyclical phases: sorption, desorption, sensible cooling, condensation, and water collection, fundamentally governed by the sorbent material's water uptake capacity and kinetics, which are intrinsically linked to its engineered porous architecture (Figure 7a) [12, 151, 152]. Water capture occurs via physisorption (van der Waals forces) and chemisorption (stronger hydrogen or covalent bonds), with system productivity highly dependent on the operational mode: monocyclic, batch, or continuous (Figure 7b,c) [16, 129, 153].

**FIGURE 7:**
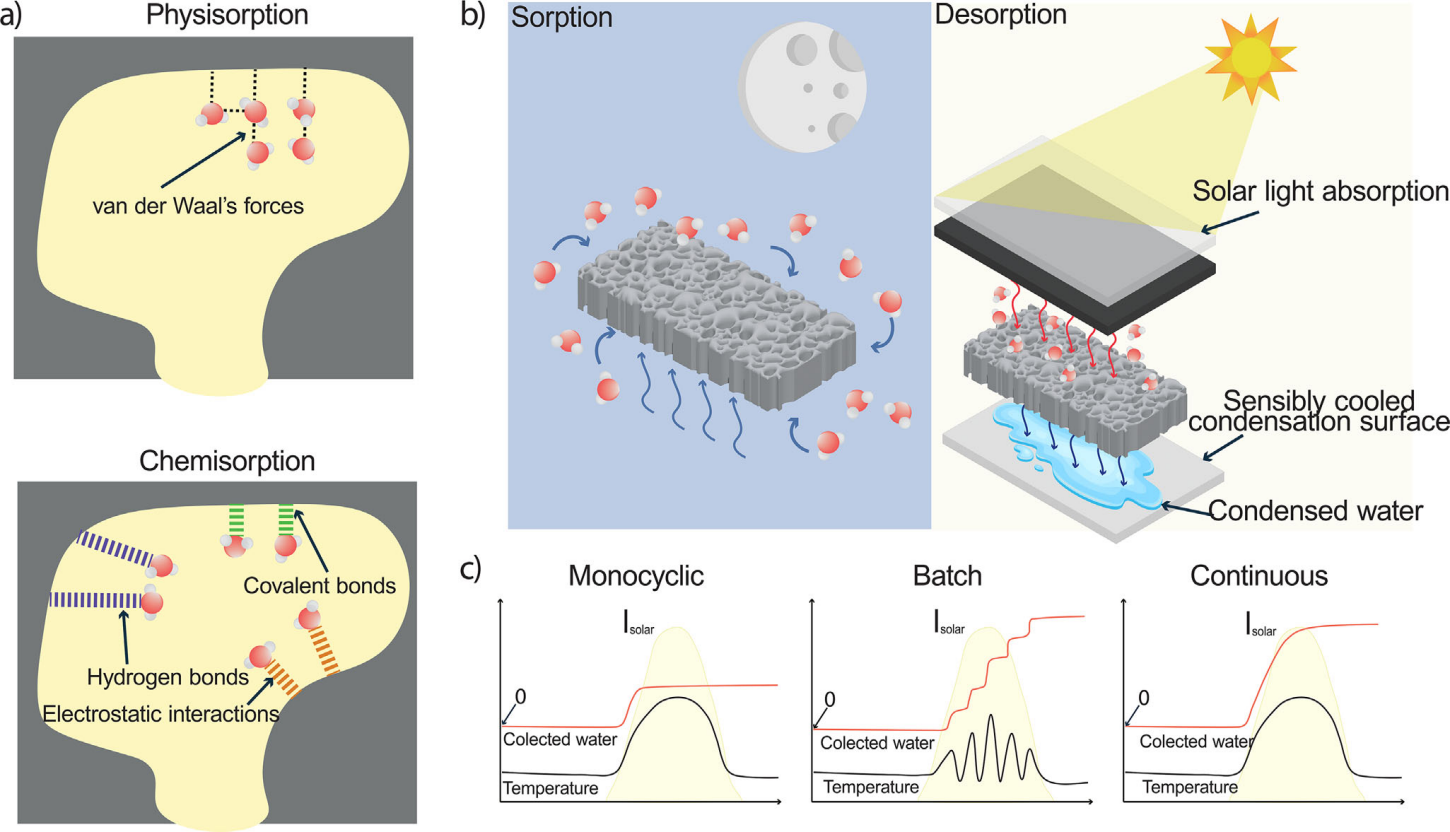
Graphical representation of the working principles of sorption‐based atmospheric water harvesting (SAWH). (a) Physisorption and chemisorption mechanisms and the dominant forces governing pore‐level water adsorption. (b) Sorption–desorption processes illustrating the water uptake and release cycles. (c) Typical operating modes of SAWH devices.

#### 5.1.2 Material Selection and Trade‐Offs

Material selection for SAWH requires navigating critical trade‐offs between water uptake capacity, sorption–desorption kinetics, and energy demand [17]. Hydrogels offer exceptional capacity, with polyacrylamide‐based composite hydrogels and salt–hydrogel systems typically achieving 1–3 g g^−1^ and up to ∼4.2 g g^−1^ at 60%–90% RH, albeit with diffusion‐limited kinetics often on the scale of hours [154, 155]. In contrast, conventional zeolites and silica gels exhibit fast kinetics but more modest capacities (∼0.2–0.3 g g^−1^ at 30%–60% RH) and require elevated regeneration temperatures (≥150–200°C), increasing energy demand [151, 152]. Metal–organic frameworks (MOFs) and covalent organic frameworks (COFs) provide a tunable middle ground, with typical uptakes of ∼0.3–1.5 g g^−1^ depending on structure and humidity [17, 151, 156] for example, LiCl@MIL‐101(Cr) reaches 0.77 g g^−1^ at ∼30% RH [157], while CaCl_2_@MOF‐808‐11.8 achieves 0.56 g g^−1^ at 30% RH, about seven times higher than pristine MOF‐808 (∼0.08 g g^−1^) [158]. Scalable composites such as hygroscopic salt‐embedded composites (HSCMs) demonstrate 3.75, 2.83, and 1.47 g g^−1^ at 90%, 70%, and 30% RH, respectively, in PTFE‐encapsulated systems with high salt loading, though deliquescence and leakage must be carefully managed Hygroscopic porous polymers (HPPs) typically deliver ∼0.5–2 g g^−1^ with faster cycling and good photothermal compatibility. At the system level, these material metrics translate to reported productivities ranging from ∼0.45 kg kg^−1^ day^−1^ to 0.70 kg kg^−1^ day^−1^ for LiCl@MIL‐101(Cr) prototypes [157], 1.8 kg kg^−1^ day^−1^ for CaCl_2_@MOF‐808‐based solar devices [158], and ∼560 mL m^−2^ day^−1^ for high‐salt HSCM panels under natural sunlight [159]. More integrated architectures, such as the solar–wind‐coupled hygroscopic gel system reported in 2024, achieved 14.9 L m^−2^ day^−1^ indoors and 3.5–8.9 L m^−2^ day^−1^ outdoors with solar concentration [133], while multilayer‐cyclic strategies have demonstrated ∼26% solar‐to‐water efficiency with per‐cycle yields on the order of 200+ mL [160]. Performance is ultimately dictated by hierarchically porous structures that facilitate vapor transfer (macropores) and high‐surface‐area interaction (meso/micropores), combined with optimized thermal management. Integrating photothermal agents (e.g., CNTs) enhances solar‐driven desorption, while RC materials aid condensation [161, 162]. Structural innovations via 3D printing, aligned microchannels, and nature‐inspired designs are therefore crucial to accelerate mass transfer and minimize thermal losses, enabling rapid multi‐cycle operation and higher daily yields [130, 163, 164]. Future scalable, multifunctional systems will depend on combining these advanced sorbents with efficient cyclic thermal design to sustain high productivity across diverse climates [165, 166, 167].

Collectively, these trade‐offs reveal that the performance limits of SAWH systems are increasingly imposed not by sorbent chemistry alone, but by the inability to independently control water uptake capacity, vapor transfer resistance, and thermal response within a single material architecture [153, 168, 169]. Conventional synthesis routes typically generate stochastic pore networks in which high internal surface area is intrinsically coupled to tortuous diffusion pathways and poor effective thermal conductivity. As a result, adsorption is slowed by vapor transfer resistance, while desorption is limited by low thermal conductivity and thermal diffusivity, leading to pronounced temperature gradients across the sorbent thickness. In practical devices, this manifests as significant temperature drops from the heated surface toward the interior of the bed, incomplete regeneration, and extended cycle times [169, 170]. AM and pore engineering directly address these limitations by enabling deterministic control over pore size distribution, orientation, and hierarchy [46]. Designed architectures can incorporate aligned macroporous channels for rapid vapor transfer, coupled with meso‐ and microporous domains for high‐capacity sorption, while simultaneously improving effective thermal pathways through controlled solid‐phase connectivity [171, 172]. By increasing effective thermal conductivity and diffusivity, and minimizing axial temperature gradients within the sorbent layer, such structures enable more uniform heating, faster desorption, and reduced specific regeneration energy [173, 174]. Moreover, architected monolithic forms facilitate the integration of photothermal absorbers and RC interfaces directly within the structural framework [162], enabling spatially optimized heat input during desorption and enhanced passive heat rejection during condensation [175]. As discussed in the following sections, 3D printing therefore enables a shift from material‐centric optimization toward architecture‐driven SAWH design, opening opportunities for continuous operation, scalable manufacturing, and emerging concepts such multifunctional sorbent thermal management systems.

#### 5.1.3 Review of 3D‐Printed SAWH Materials

While AM is gaining importance, the literature remains sparse and fragmented. Current progress can be categorized into three principal domains: (1) the direct 3D printing of hygroscopic sorbents, (2) the fabrication of sorbent matrices or integrated heat exchangers via AM, and (3) the holistic AM‐driven design of complete SAWH systems. This review focuses specifically on the first domain: the direct printing of sorbent materials themselves. Notably, within this space, DIW has been the only AM technique to consistently demonstrate scientifically significant and functionally relevant results for SAWH sorbent fabrication, owing to its ability to process highly loaded, rheologically tuned inks while preserving porosity and hygroscopic functionality.

In a seminal contribution to the first category, Chen et al. [37] reported the fabrication of a superelastic, 3D‐printed monolithic sorbent derived from cellulose nanofibrils (CNFs) (Figure 8a). The synthesis strategy employed DIW in conjunction with CaCl_2_ cross‐linking and freeze‐drying, yielding a hierarchically porous monolith with both macro and microporosity. The incorporation of CaCl_2_ not only introduced hygroscopic functionality enabling tunable humidity‐driven water sorption but also facilitated ionic cross‐linking with CNF carboxylate groups. This process disrupted the native hydrogen bonding network within the CNF matrix, imparting enhanced elasticity through molecular plasticization. Macroporosity, governed by the 3D printing resolution, was controlled at the millimeter scale, while microporosity arose from freeze‐drying and ice‐templated crystallization. Notably, in the absence of CaCl_2_, the resulting structure exhibited loosely packed CNF struts with high porosity. By contrast, inclusion of CaCl_2_ yielded denser architectures with well‐defined grid‐like morphologies and rectangular pores, characterized by strut widths of approximately 300 µm. The sorption performance demonstrated a strong dependence on both salt concentration and RH. At 43% RH, water uptake kinetics ranged from 1.58%/min (0.1 M CaCl_2_) to 0.25%/min (2 M CaCl_2_), with water uptake capacities reaching up to 56 wt%. The decrease in sorption rate at higher CaCl_2_ concentrations is attributed to the densification and reduced porosity of the structure. Under elevated humidity conditions (95% RH), the material achieved a maximum water uptake of 260 wt%, with uptake rates reaching 2.08%/min and equilibrium attained after approximately 25 h. Importantly, the monoliths demonstrated excellent cyclic stability, retaining approximately 69% of their initial water uptake capacity after 10 absorption–desorption cycles. In addition to their primary function in water sorption, the structures exhibited promising multifunctionality, notably in strain sensing applications. This arises from the material's superelasticity and the ionic conductivity enabled by Ca^+2^ integration, which together confer high mechanical robustness and conductive responsiveness.

**FIGURE 8:**
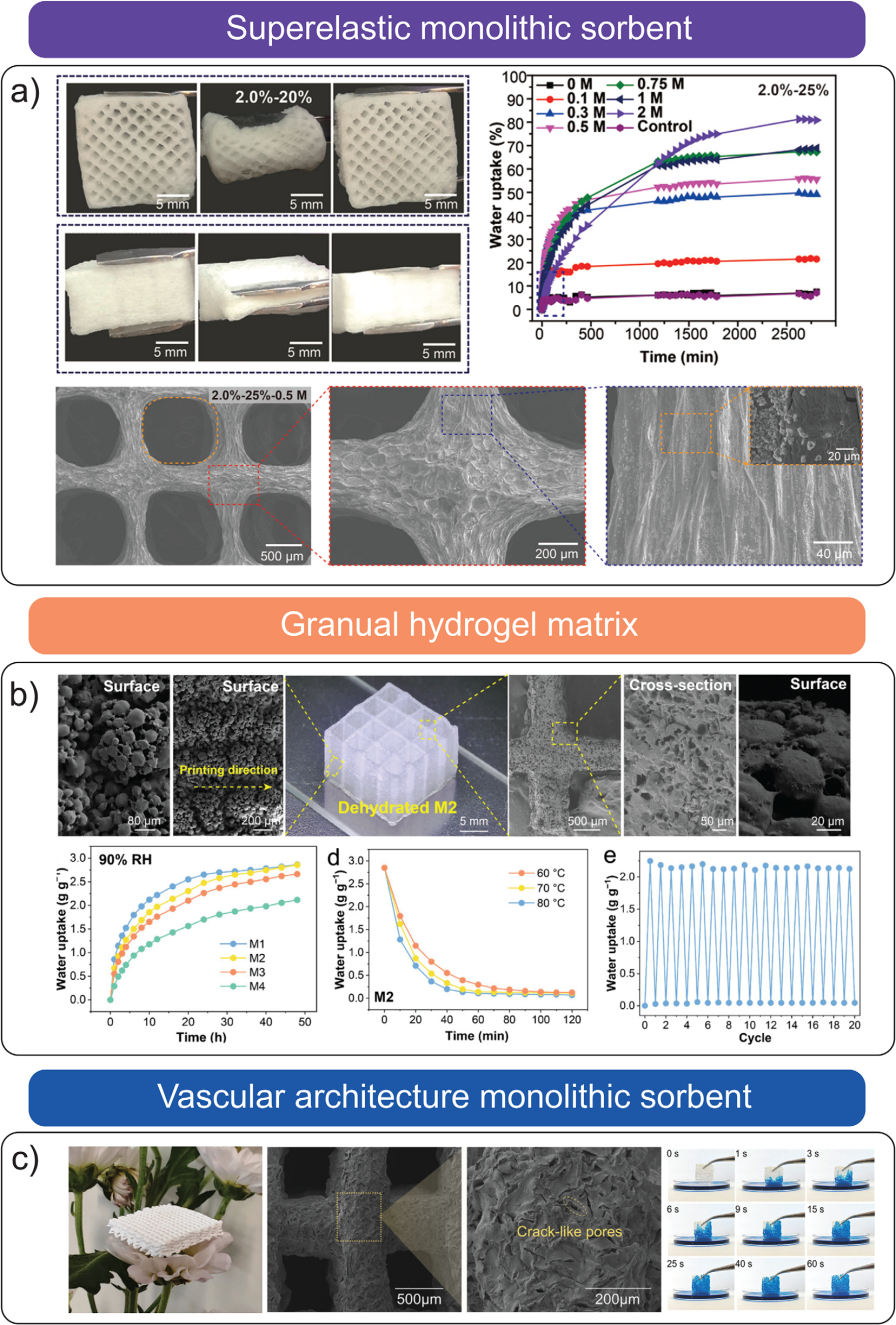
Representative 3D‐printed sorbent materials for SAWH, structural, morphological, and sorption properties. (a) Shape recovery behavior of 3D‐printed CNF monoliths (2–20 wt%) under in‐plane and out‐of‐plane compression, cyclic stress–strain performance (20 cycles), SEM images showing the salt‐crosslinked porous structure, and water uptake kinetics at 43% RH for different salt contents. Reproduced with permission [37]. Copyright 2021, Wiley‐VCH. (b) Morphology of freeze‐dried sorbents and their water vapor sorption and desorption characteristics under high humidity and temperature variations. Reproduced with permission [49]. Copyright 2025, Wiley‐VCH. (c) SEM and photographic images of CASN monolith featuring uniform macropores (∼600 µm) with microcracked surfaces, along with capillary force measurements demonstrating enhanced water transfer properties. Reproduced with permission [176]. Copyright 2025, Wiley‐VCH.

Wu et al. [49] developed innovative 3D‐printed hygroscopic matrices leveraging granular hydrogel inks via DIW, incorporating jammed sodium alginate microparticles (SA‐HMPs) cross‐linked with zwitterionic copolymer networks (P(PDMAPS‐co‐HEAA)) and LiCl for synergistic moisture capture and structural reinforcement (Figure 8b). The matrices feature hierarchical porosity across millimeter‐scale lattice channels, micrometer‐scale wrinkled surfaces, and nanometer‐scale granular assemblies, optimizing surface area and mass transfer for superior sorption/desorption kinetics. Post‐printing UV curing and ionic cross‐linking ensure mechanical robustness, with 20 wt% HEAA enhancing compressive strength by 5.7. Optimized at 0.1 g mL^−1^ LiCl, the formulation exhibits negligible salt leakage via the “salt‐in” effect and large‐volume swelling, supporting reversible cycles across 10%–100% RH using low‐grade heat sources. The design achieves 80% porosity and a specific surface area 5.8‐fold higher than aerogels, reducing raw material needs by 53%. Water uptake was evaluated at 90% RH and 25°C, yielding 2.85 g g^−1^ over 48 h, 1.4‐fold greater than conventional hygroscopic aerogels, while filaments reached 3.10 g g^−1^. Kinetic assessments over multi‐hour cycles confirmed rapid vapor diffusion and structural durability, with the hierarchical architecture acting as both a hygroscopic scaffold and transfer enhancer. Tailoring matrix geometries from cubic to cylindrical forms further boosted application‐specific performance, such as on‐demand defogging. The system enables desorption using low‐grade heat (e.g., 80°C hot air).

Chen et al. [176] reported the development of a monolithic sorbent structure inspired by the vascular architecture of black spruce, leveraging biomimetic design principles to enhance both mass and heat transfer (Figure 8c). The monolith was fabricated using DIW, followed by ionic crosslinking and freeze‐assisted solidification, resulting in a hierarchically porous structure comprising vertically aligned mesopores (∼600 µm) and microscale crack‐like surface pores. This multiscale porosity facilitates efficient vapor transfer and capillary‐driven retention of the hygroscopic phase. The composite formulation consists of sodium alginate (SA) as the biopolymer matrix, hydrophilic fumed silica HF‐SiO_2_ to enhance structural integrity and sorption kinetics, boron nitride BN to boost thermal conductivity achieving a 3.3‐fold improvement and LiCl as the active hygroscopic component. Sorption performance was evaluated at 25°C under varying RH conditions. The material achieved water uptakes of 0.42, 0.76, and 1.18  g g^−1^ at 30%, 60%, and 90% RH, respectively, corresponding to 88.4%–89.4% of equilibrium capacity within 2 h. Initial sorption rates increased with humidity, from 0.70  g g^−1^ h^−1^ at 30% RH to 1.50  g g^−1^ h^−1^ at 90% RH. Desorption behavior was assessed under forced convection at an air velocity of 2.0 m/s and ∼78 °C, with 81.8% of the adsorbed water released within 20 min. The desorption ratio within 15 min improved from 52.9% to 70.5% as airflow velocity increased from 1.0 to 2.0 m s^−1^. The regeneration process demonstrated exceptional energy efficiency, requiring only 0.013 kWh per cycle. Notably, no salt leakage was observed during full hydration, and the material retained its performance over 25 consecutive sorption–desorption cycles conducted at 60% RH and 30 °C (sorption) and 80 °C (desorption). Compared to conventional block sorbents, the CASN‐Li monolith exhibited a 2.1‐fold increase in sorption rate, underscoring its superior kinetics and practical viability. In a chamber 6750 times its own volume, the material effectively reduced RH from 90% to 60% in under 25 min, demonstrating its applicability for energy‐efficient dehumidification and SAWH applications.

Zhu et al. [46] developed a 3D‐printed bilayer scaffold comprising a functional architecture tailored for integrated sorption and photothermal‐driven desorption (Figure 9a). The bilayer design consists of a bottom sorbent layer composed of CNF and LiCl with hierarchical porosity, and a top photothermal layer composed of CNF and CNT. This structural configuration enables efficient water capture and solar‐driven regeneration within a single, monolithic device. The porous CNF matrix provides effective confinement of LiCl, mitigating salt agglomeration while preserving high porosity even under significant salt loading. SEM imaging confirmed uniform LiCl distribution throughout the scaffold's porous network. The hierarchical pore structure significantly enhances sorption kinetics by increasing the active surface area and shortening vapor diffusion pathways, enabling multidirectional water transfer and evaporation from both the scaffold surface and internal grid walls. The CNF/LiCl‐1.5 scaffold exhibited a water uptake of 1.59  g g^−1^ within 12 h at 95% RH, with a sorption rate of 0.13  g g^−1^ h^−1^ outperforming traditional freeze‐dried CNF aerogels (0.08  g g^−1^ h^−1^). The effective RH response range was broad, spanning from 11% to 95% RH. At low humidity (11% RH), the scaffold achieved a total uptake of 0.39  g g^−1^ with a rate of 0.05 g g^−1^ h^−1^, while under high humidity (95% RH), it reached 1.65  g g^−1^ with a rate of 0.2 g g^−1^ h^−1^, characterized by an initial rapid uptake phase followed by gradual saturation. Desorption was driven by solar irradiation at 1 kW m^−2^, where the bilayer scaffold rapidly reached a surface temperature of ∼55°C within 2 min.

**FIGURE 9:**
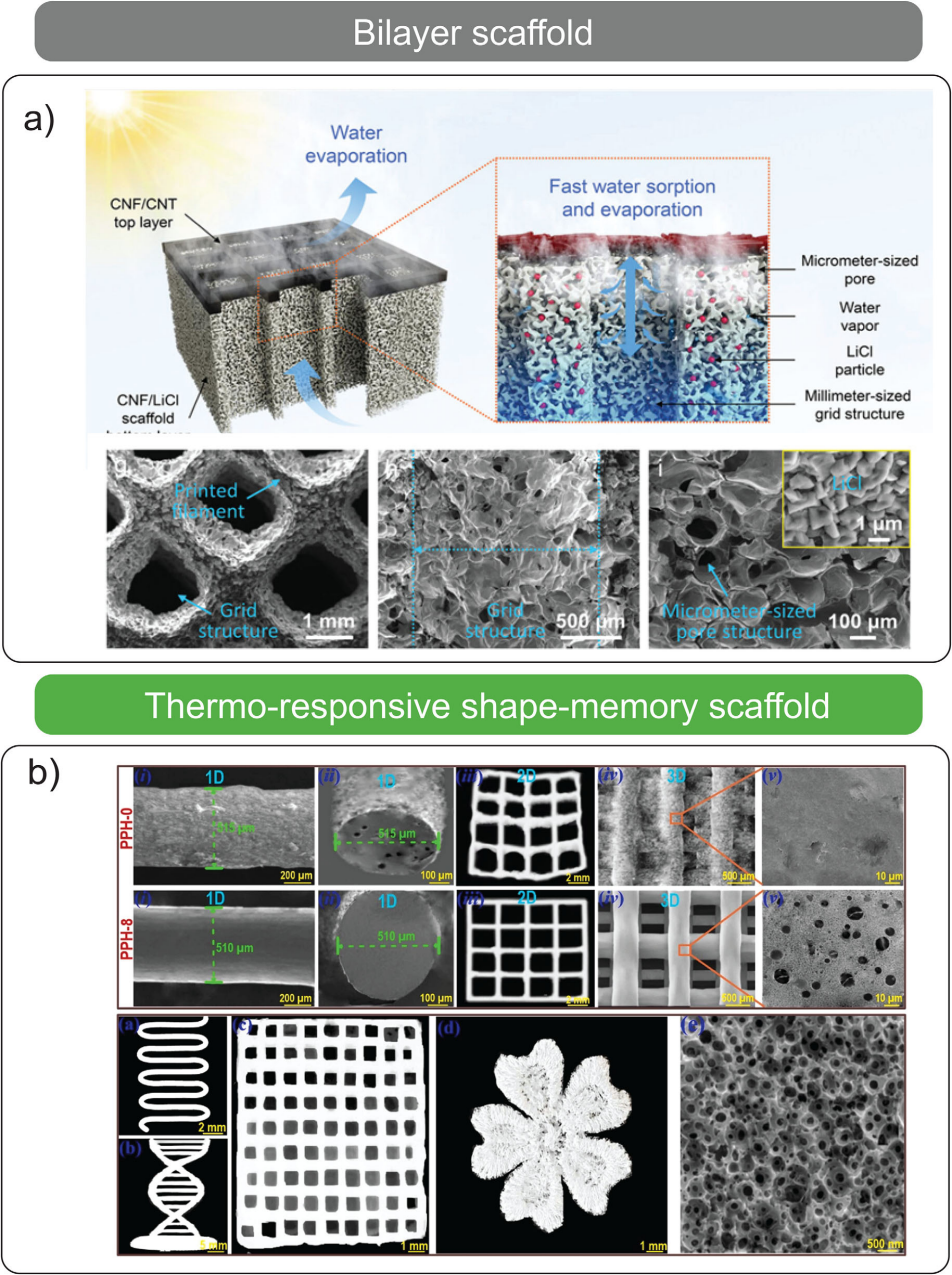
Structural design and printability of advanced 3D‐printed SAWH sorbents. (a) Schematic illustration and SEM micrographs of bilayer CNF/LiCl‐2.0 scaffolds showing the top‐view, vertical‐section morphology, and micrometer‐scale pore structure with LiCl particle loading. Reproduced with permission [46]. Copyright 2024, Wiley‐VCH. (b) Printability and structural versatility of PPH inks (PPH‐0 and PPH‐8), from 1D filaments to 3D architectures, including SEM images of microstructures and complex printed geometries (zigzag, helix, lattice, and flower‐like structures). Reproduced with permission [79]. Copyright 2024, Elsevier.

Under these conditions, it achieved a peak evaporation rate of 1.43 kg m^−2^ h^−1^ approximately 2.4× that of CNF/LiCl‐1.5 without the photothermal layer and a solar‐to‐vapor conversion efficiency of 80.3%. The scaffold demonstrated robust cyclic performance, maintaining 80%–90% of its original evaporation efficiency over repeated cycles, with water uptake retention of 85% attributed to volumetric shrinkage rather than LiCl loss. Field evaluations further validated the material's performance, with the bilayer scaffold achieving 1.28  g g^−1^ water uptake overnight under ambient conditions (50%–70% RH), and a daily water production rate of 0.63  g g^−1^ over five cycles. Compared to conventional aerogels, the scaffold exhibited a 1.6‐fold improvement in sorption rate and a 2.4‐fold enhancement in evaporation efficiency. The integrated bilayer design demonstrated exceptional recyclability, mechanical durability, and solar responsiveness, underscoring its potential as a scalable, sustainable, and high‐performance sorbent system for AWH applications.

In a related advancement, Shahbazi et al. [79] demonstrated UV‐assisted DIW 4D printing of thermo‐responsive, shape‐memory polyHIPE monoliths stabilized with hydrophobic SiO_2_ nanoparticles (Figure 9b). The printed structures exhibited high porosity (∼85%) with interconnected open cells (10–30 µm), enabling solvent uptake up to 15.5 g g^−1^ and stable performance over more than 10 sorption–desorption cycles without pore collapse. Their programmable deformation and temperature‐triggered recovery were enabled by the tailored porous architecture and viscoelastic matrix, highlighting how architected porosity can simultaneously govern mass transfer and dynamic structural functionality. This work further illustrates the potential of hybrid 3D/4D printing strategies for adaptive sorption systems beyond static material design.

A conclusion can be drawn that hierarchical porosity is central to achieving multiscale functionality in AM‐enabled SAWH systems. Various AM resolutions imposed by different printing techniques generate designed macropores (100 µm–mm) that govern bulk vapor and liquid transfer, reduce effective diffusion length, and improve heat transfer, while micro‐ and mesopores introduced via post‐processing (freeze‐drying, templating or granules) provide high surface area and sorption sites, together, this hierarchy enhances vapor sorption/desorption kinetics (up to 2.1‐fold in Ref. [176]), mitigates pore clogging and salt leakage, and stabilizes hygroscopic phases. Interconnected, aligned, or biomimetic architectures (vertical channels in Ref. [176], wrinkled surfaces [49] enable improved capillary transfer of condensed liquid water and reduced resistance to vapor diffusion, supporting multidirectional vapor/liquid transfer and more uniform thermal fields. AM precision further allows lattice or grid geometries that optimize coupled heat and mass transfer, while matrices such as CNF or sodium alginate confine hygroscopic salts (LiCl, CaCl_2_) responsible for uptake. The AM process allows for the incorporation of additives in the inks such as (SiO_2_, BN, CNT) for strength, conductivity, or photothermal properties, indirectly influencing pore stability (up to a 5.7‐fold compressive strength in Ref. [49]). Reported cyclic stability and minimal salt leakage indicate that AM‐fabricated sorbents can match or exceed the structural integrity of conventionally prepared sorbents produced via casting, freeze‐drying, or granulation [177, 178], while offering superior architectural control and scalability. A more comprehensive comparison between pore architecture and performance metrics can be found in Table S1.

The diverse, mostly hydrogel sorbents presented in this chapter, all fabricated via DIW, showcase how AM enables a move beyond simple material substitution toward the rational design of integrated, application‐specific architectures. Their distinctions arise from targeted structural engineering: the superelastic monolith [37] is defined by ionic cross‐linking that provides exceptional mechanical resilience for cyclic use, the granular hydrogel matrix [49] leverages jammed microparticle inks to create a unique hierarchical porosity that maximizes surface area and sorption kinetics, the vascular architecture scaffold [176] employs biomimetic vertical channels to optimize rapid mass and heat transfer and the bilayer scaffold integrates distinct functional zones for combined sorption and solar‐driven regeneration within a single monolith. The overarching comparison reveals that AM's primary value lies in its simultaneous and precise control over geometry, composition, and functionality. This allows researchers to tailor not just the pore hierarchy for enhanced transfer and kinetics, but also to embed secondary properties such as elasticity [49], thermal conductivity or photothermal response directly into the sorbent's structure [46, 79]. Consequently, these works collectively demonstrate that AM is not merely an alternative fabrication route, but a transformative tool for synthesizing tailor‐made, high‐performance sorbents with built‐in multifunctionality, establishing a new foundation for next‐generation SAWH systems.

### 5.2 3D Printing for Radiative Cooling and Radiative Atmospheric Water Harvesting

#### 5.2.1 Introduction to RC and RAWH

RC enables passive heat dissipation by simultaneously reflecting incoming solar radiation (high reflectance in the 0.3–2.5 µm range) and emitting thermal radiation through the atmospheric transparency window (high emissivity in the 8–13 µm band) [163], allowing surfaces to reach temperatures 5°C–10°C below ambient under favorable conditions (Figure 10a) [179, 180]. Radiative atmospheric water harvesting (RAWH) exploits this sub‐ambient cooling to induce condensation of atmospheric water vapor when the surface temperature falls below the local dew point, thereby extending RC from a thermal management strategy to an energy‐passive water production mechanism [181]. While RC and RAWH share a common thermodynamic foundation, RAWH introduces additional requirements related to condensation dynamics, droplet transfer, and water collection efficiency.

**FIGURE 10:**
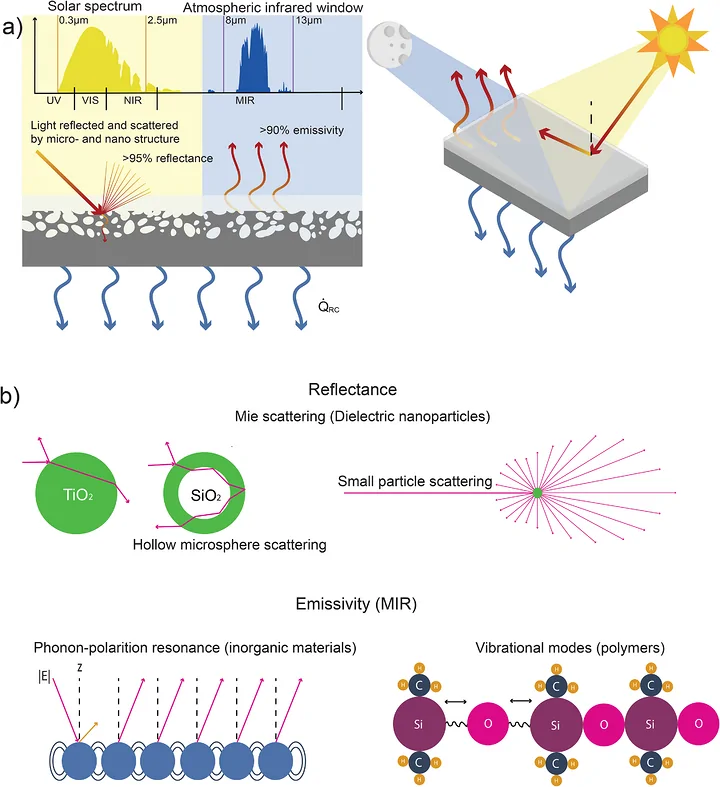
Schematic illustration of the fundamental working principles of radiative cooling. (a) Interaction of light with the surface, showing strong solar reflectance in the 0.3–2.5 µm wavelength range and selective thermal emission in the atmospheric transparency window (8–13 µm), enabling passive heat dissipation to outer space. (b) Underlying optical mechanisms governing reflectance and emissivity: solar reflectance achieved through Mie scattering by dielectric nanoparticles, and mid‐infrared (MIR) emissivity originating from phonon–polariton resonances in inorganic materials and molecular vibrational modes in polymers.

#### 5.2.2 Material Selection and Device Optimization

The performance of RC devices is primarily governed by optical selectivity, which is achieved through carefully chosen materials and microstructural designs. High solar reflectance (>95%) and mid‐infrared (MIR) emissivity (>90%) are commonly realized using dielectric nanoparticles such as SiO_2_ and TiO_2_ to induce Mie scattering, metallic reflectors (e.g., Ag), and polymers including PVDF‐HFP and PDMS whose molecular vibrational modes (C–F, Si–O) and phonon–polariton resonances support selective thermal emission (Figure 10b) [182, 183, 184, 185]. Structural hierarchy further enhances RC performance: bioinspired surface architectures modeled after beetle shells [186] or butterfly wings [187, 188] improve light scattering and, when relevant, facilitate droplet nucleation and shedding, while porous networks with intermediate porosity (∼50%–70%) increase effective scattering area without excessive thermal conduction [184].

When RC architectures are adapted for RAWH, additional structural and functional considerations become critical. Surface micro‐ and macrostructures must support efficient condensation, directional liquid transfer that will allow for faster water removal from the condensation surface, and water collection, while maintaining the optical properties required for sub‐ambient cooling. AM plays a key role in addressing these coupled requirements by enabling the fabrication of functional macro‐ and microstructures, such as V‐shaped collectors that enhance interior natural convection and reduce heat and mass transfer resistance [189] or reconfigurable devices capable of switching between solar heating and RC modes [190]. Beyond geometric control, emerging optimization strategies increasingly combine AI‐driven design of pore and feature sizes with scalable manufacturing approaches [191], including roll‐to‐roll processing [192], to produce durable and climate‐adaptable systems. Future developments are expected to further integrate RC surfaces with sorption‐based materials and scalable fabrication routes, enabling multifunctional platforms for sustainable cooling and decentralized water harvesting [193].

However, evaluating the cooling performance of these architected RC systems remains challenging. Conventional full‐spectrum characterization, including reflectance, transmittance, absorptance, and mid‐infrared emittance, does not always directly predict functional cooling behavior in AM‐enabled structures. The hierarchical porosity, complex interfaces, and multiscale geometries introduced by AM can cause the effective optical response to deviate from bulk or thin‐film models. Moreover, measured cooling temperatures are not necessarily proportional to spectral properties alone, as environmental conditions, parasitic heat exchange, and measurement methodology strongly influence the reported values. These limitations should therefore be explicitly acknowledged when comparing RC performance across studies. Rather than treating optical spectra as sufficient predictors, the following discussion emphasizes how specific structural parameters govern spectral response, heat transfer, and ultimately net cooling power.

#### 5.2.3 Review of 3D‐Printed RC and RAWH Materials and Devices

Using DIW, Shi et al. [45] fabricated a wood‐inspired methyltrimethoxysilane/silica/cellulose nanofiber (MSC) aerogel that combines axially aligned millimeter‐scale channels with mesopores (3–50 nm) generated during freeze‐drying (Figure 11a). This multiscale architecture promotes strong Mie scattering from SiO_2_ nanoparticles while suppressing conductive heat transfer, yielding high solar reflectance (94.18%), high mid‐infrared emissivity (96.09%), reduced thermal conductivity (59.4 mW m^−1^ K^−1^), and sub‐ambient cooling of 6.16°C under direct sunlight, demonstrating the suitability of DIW‐engineered pore networks for RC in micro‐ and nanoelectronic thermal management.

**FIGURE 11:**
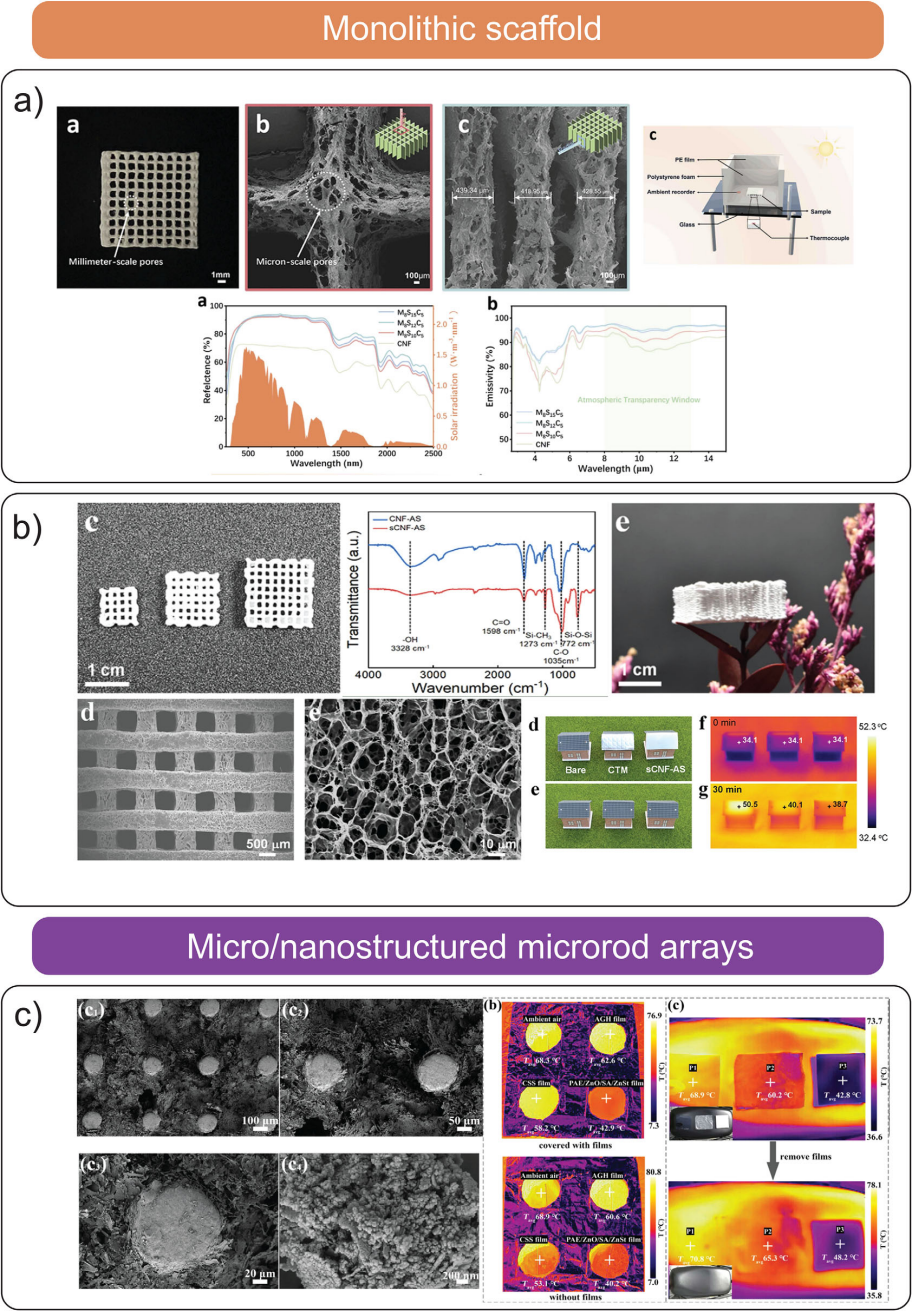
Representative 3D‐printed and engineered materials for passive radiative cooling. (a) Morphological and structural characterization of 3D‐printed MSC coolers showing digital and SEM images of the filament architecture and cross‐sectional aerogel structure. Optical analysis demonstrating high solar reflectance and MIR emissivity, alongside a schematic of the custom setup used for daytime radiative cooling performance testing. With permission from Reproduced with permission [45]. Copyright 2024, Elsevier. (b) Structural and thermal performance of sCNF‐AS coolers, including morphology (size and thickness), lattice and pore structures, and lightweight characteristics. Infrared thermographs of roof models with and without sCNF‐AS coverings show significant temperature reduction after 30 min of direct sunlight exposure, confirming effective solar‐thermal regulation. Reproduced with permission [194]. Copyright 2025, Elsevier. (c) SEM images of hierarchical PAE/ZnO/SA/ZnSt films displaying nanoscale spheres and nanobelts enabling a superhydrophobic Cassie state. IR thermographs illustrate enhanced surface cooling performance on chamber surfaces under sunlight exposure. Reproduced with permission [48]. Copyright 2025, ACS.

Zhu et al. [194] advanced DIW‐enabled porous radiative cooling materials by developing a silanized cellulose nanofiber aerogel scaffold (sCNF‐AS) that overcomes the intrinsic hydrophilicity and flammability of conventional CNF aerogels while enhancing optical and mechanical robustness (Figure 11b). By incorporating polymerizable methyltrimethoxysilane (MTMS) directly into the ink, continuous post‐deposition crosslinking forms polymethylsilsesquioxane (PMSQ) networks that improve interlayer adhesion and impart hydrophobicity (contact angle 142.3°) and flame retardancy. The resulting aerogel exhibits a hierarchical nanoporous architecture with narrowly distributed pore sizes (1–6 µm), deliberately matched to solar wavelengths to maximize Mie scattering, yielding high solar reflectance (94.2%) and mid‐infrared emissivity (95.6%). This pore‐size refinement enables strong daytime and nighttime radiative cooling, delivering a theoretical cooling power of 72.2 W m^−2^ under standard solar conditions and 130.2 W m^−2^ at night, with outdoor tests confirming a sub‐ambient temperature reduction of 5.8°C. Compared to traditional CNF aerogels with broader pore distributions (10–50 µm), the sCNF‐AS demonstrates improved optical efficiency while maintaining high porosity (97.3%) and mechanical integrity, positioning it as a scalable and durable platform for passive daytime radiative cooling in harsh outdoor environments.

Extending this pore‐engineering paradigm toward RAWH, Xu et al. [48] combined VPP with post‐functionalization to produce superhydrophobic microrod arrays that simultaneously enhance radiative cooling and water condensation (Figure 11c). The printed hierarchical surface provides efficient light scattering, air trapping, and Cassie‐state wetting, enabling durable operation under harsh environmental conditions while achieving substantial cooling and large water harvesting rates (2018.5 ± 84.9 mg cm^−2^ h^−1^). Together, these studies illustrate how AM‐enabled control over pore geometry and surface architecture underpins both RC‐dominated thermal regulation and RAWH‐oriented condensation and collection.

Beyond static RC architecture, AM has enabled dynamically adaptive and multifunctional thermal management systems. Liu et al. [39] demonstrated a 4D‐printed, thermoresponsive environmentally adaptive membrane (EAM) based on PNIPAM that integrates switchable RC and heating within a single structure. By combining TPP, DIW, and PNIPAM's volume‐phase transition, the biomimetic flower‐like architecture reversibly transitions between open (heating) and closed (cooling) states at ∼32°C within 1 min, producing a ∼35% emissivity difference in the atmospheric window (8–13 µm). This dynamic pore exposure enables net cooling or heating powers up to 185 W m^−2^, illustrating how architected porosity and stimulus‐responsive materials can actively modulate radiative heat transfer. Complementarily, Hazarika et al. [42] introduced a hierarchically porous, 3D‐printed textile composite that synergistically combines radiative cooling with directional wick‐evaporative cooling. Gradient pore sizes across hydrophobic PDMS and hydrophilic PES/SiO_2_ layers enable unidirectional moisture transfer, while Ag‐decorated ZTO nanorods and SiO_2_ nanoparticles provide high solar reflectance (89.5%) and MIR emissivity (91.8%).

Biomimetic and scalable AM strategies further highlight how pore architecture governs optical and thermal performance. Inspired by cicada microspikes, Liu et al. [195] developed a porous composite (Bio‐PC) integrating heart‐shaped cross‐sections and surface microhumps via DIW and phase separation, achieving strong Mie scattering, high solar reflectance (97.6%), and MIR emissivity (95.5%), with stable sub‐ambient cooling across diverse climates (Figure 12a). In parallel, Zhou et al. [36] reported a 3D‐printable nonporous polymer matrix composite (PMC) that achieves high solar reflectance (96.2%) and strong MIR emissivity (>90%), enabling a sub‐ambient temperature reduction of 6.1°C and a cooling power of 85 W m^−2^ under direct sunlight (∼747 W m^−2^ irradiance) (Figure 12b). The composite incorporates air pores (∼200 nm–2 µm) within the nonporous PE matrix to enhance solar reflectance via Mie scattering, while an optimized SiO_2_ particle distribution boosts emissivity in the atmospheric window (8–13 µm). Importantly, this scalable production strategy eliminates the need for costly silver reflective layers and photonic multilayers, offering a cost‐effective polymer‐based alternative to conventional vacuum‐deposited coatings. Extending this concept, Park et al. [149] employed FDM to print hollow silica nanoparticles (400–700 nm) using a sol–gel emulsion approach, achieving ultrahigh solar reflectance (98%) and MIR emissivity (93%) with stable outdoor performance. The resulting structures achieved a sub‐ambient cooling of 2.6°C under 928 W m^−2^ solar irradiance, with stable performance maintained over 13 days of outdoor testing, highlighting their promise for solar panel and electronic cooling.

**FIGURE 12:**
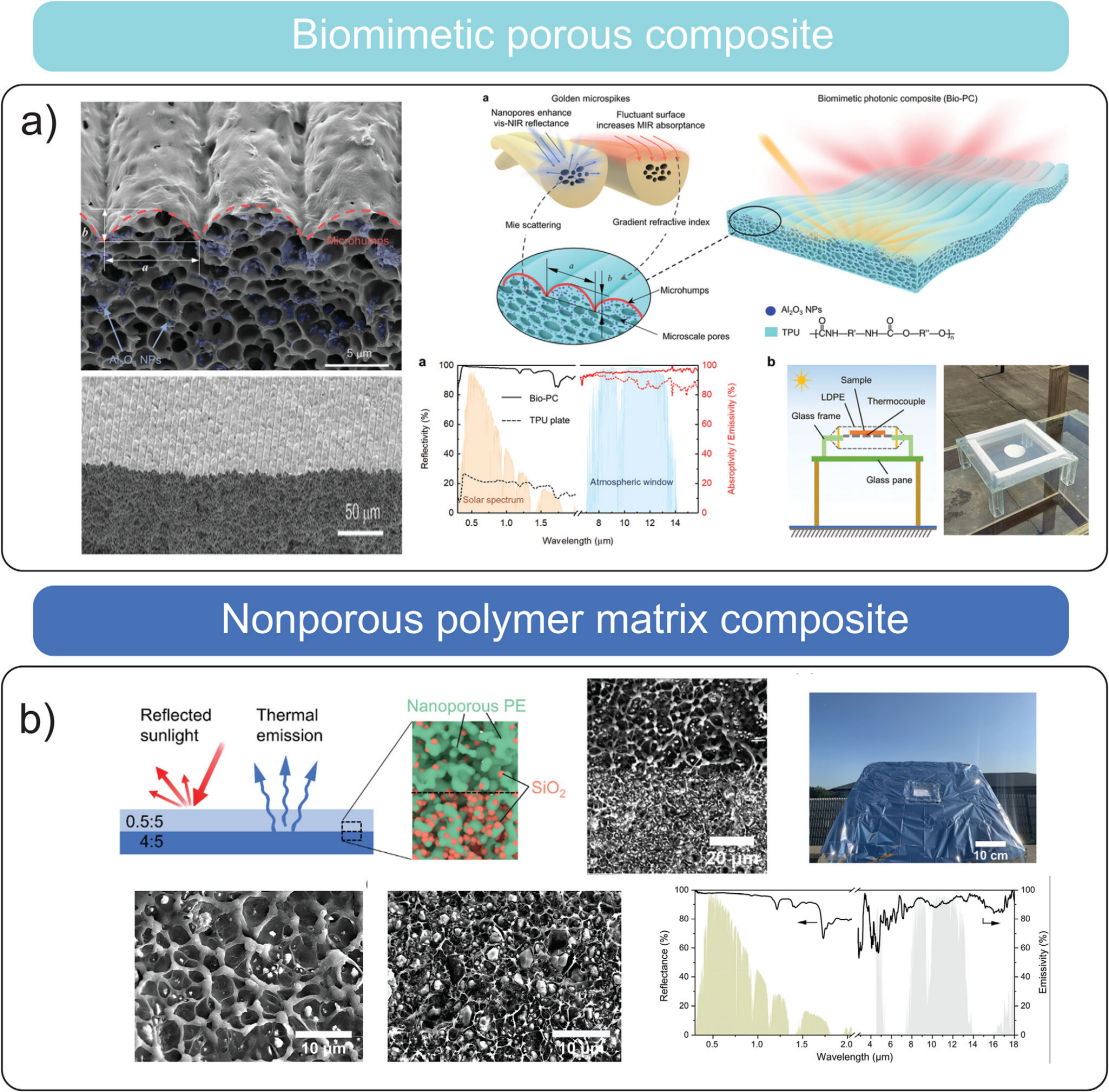
Advanced radiative cooling materials enabled by biomimetic design and nanoporous structures. (a) SEM images and schematic illustration of the Bio‐PC system inspired by natural microspike structures, showing the transition from biomimetic design to functional radiative cooling composites. Optical characterization demonstrates high solar reflectance and MIR emissivity of Bio‐PC (≈350 µm thickness) compared to a planar TPU reference. A schematic of the outdoor setup used for daytime radiative cooling performance measurements is also shown. Reproduced with permission [195]. Copyright 2021, Wiley‐VCH. (b) Schematic illustration of the nanoporous PMC film with a SiO_2_ particle concentration gradient engineered to achieve broadband solar reflectivity and strong infrared emissivity. Cross‐sectional SEM images of the upper and lower layers (SiO_2_:PE weight ratios 0.5:5 and 4:5, thicknesses 280 and 560 µm, respectively) reveal the graded pore morphology. Spectral measurements highlight enhanced reflectance and emissivity, and the experimental setup for outdoor cooling tests confirms the material's efficient passive radiative cooling performance. Reproduced with permission from Refs. [36]. Copyright 2021, ACS.

Across the reported additively manufactured RC materials, pore architecture emerges as the primary design parameter linking fabrication strategy to optical selectivity, heat transfer, and ultimately net cooling power. Table S2 summarize the quantitative relationships between pore size, porosity, refractive index, hierarchical organization, and cooling performance across the reviewed systems. Pores spanning the visible and near‐infrared wavelength range (including 1–6 µm in sCNF‐AS aerogels [194] and 200 nm–2 µm in nanoporous PMC structures [36]) maximize Mie scattering, enabling solar reflectance values above 94%. Similarly, 40‐nm mesopores combined with millimeter‐scale aligned channels in MSC aerogels enhance broadband backscattering while suppressing conductive heat transfer [45]. In contrast, VPP‐fabricated microrod arrays with ZnO nanoparticles exhibit lower solar reflectance (∼70%). This is despite hierarchical structuring. This demonstrates that geometry alone is insufficient. It is insufficient without wavelength‐scale scattering features. Nevertheless, these same structures achieve the highest reported net cooling power (152–159 W m^−2^) due to extremely high mid‐infrared emissivity (97%), indicating that RC performance depends on balancing solar rejection and infrared emission rather than reflectance alone. Porosity modulates this balance by increasing scattering density, emissive surface area, and thermal insulation. On the other hand, the 97.3% nanoporous network in sCNF‐AS [194] exhibits extremely high porosity, which suppresses thermal conduction while maintaining high reflectance and emissivity. However, structural reinforcement becomes necessary to preserve mechanical stability. Likewise, high‐refractive‐index inclusions, such as ZnO, Ag@ZTO, or Al_2_O_3_ nanoparticles, enhance scattering and emissive coupling more efficiently than lower‐contrast polymer/air systems.

The hierarchical organization of materials across multiple length scales further amplifies these effects by coupling optical management with thermal regulation. Deterministic control of refractive‐index gradients, multiscale scattering pathways, trapped air volumes, and moisture transfer without significant degradation of optical selectivity is enabled by AM, as demonstrated collectively by the following: DIW‐fabricated wood‐like channel structures [45], biomimetic microhumps with internal pores and nanoparticles [195], microrod–nanobelt assemblies [48], and bilayer wettability gradients in breathable fabrics [42]. Adaptive 4D poly(*N*‐isopropylacrylamide) (PNIPAM) systems further extend this concept by dynamically modulating pore exposure and emissivity in response to environmental conditions. In many cases, conventional full‐spectrum optical characterization alone is not enough to predict how well AM‐enabled RC systems will perform when it comes to functional cooling. This is because reflectance and emissivity measurements do not fully capture the effects of hierarchical porosity, anisotropic transfer, parasitic heat exchange, and environmental boundary conditions. Consequently, temperature reduction should be interpreted as a consequence of net radiative cooling power and environmental coupling rather than as an isolated material metric. Overall, a clear structure–property–performance relationship in AM‐enabled RC materials has been established by the reviewed studies: spectral scattering is controlled by pore size, thermal insulation and emissive surface area are governed by porosity, scattering strength is determined by refractive index, and these effects are coupled across scales by hierarchical organization to regulate net cooling power. Thus, effective RC design depends on balancing radiative, conductive, and convective heat‐transfer pathways through deterministic architectural control enabled by AM processes such as DIW, VPP, freeze‐drying, hydrothermal growth, and in situ sol–gel assembly.

### 5.3 3D Printing of Materials for Interfacial Solar Evaporation

#### 5.3.1 Introduction to ISE

ISE utilizes photothermal materials to localize absorbed solar energy at the water–air interface, driving vapor generation while minimizing conductive losses to the bulk water (Figure 13) [196]. In floating interfacial architectures, water is continuously supplied to a spectrally absorbing surface, enabling efficient steam production that can reach ∼100°C under 1 sun illumination, as demonstrated in Ref. [197]. In this case, solar irradiation is converted into localized heat, and the latent heat of vaporization is supplied by photothermal energy input.

**FIGURE 13:**
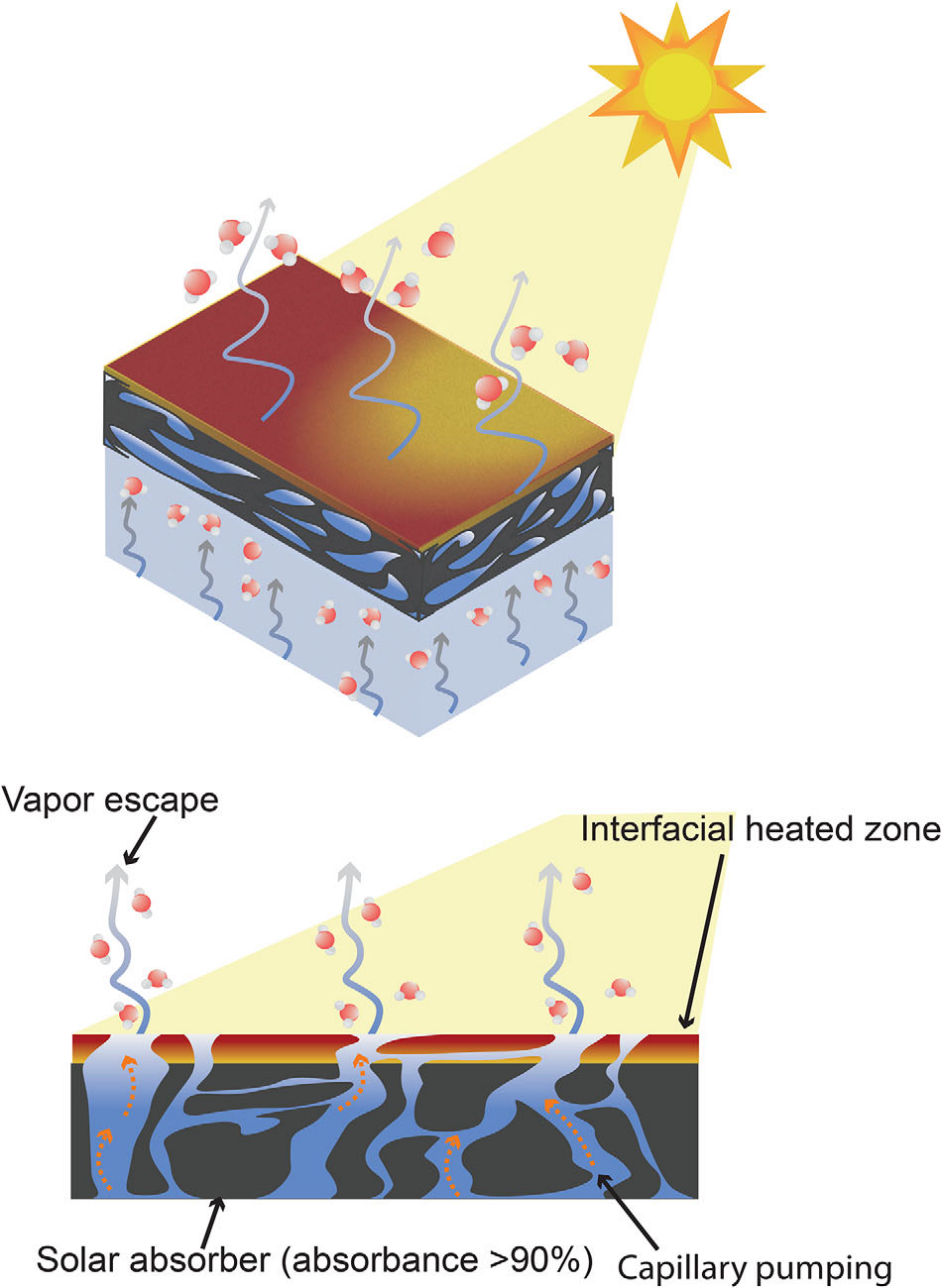
Schematic illustration of interfacial solar evaporation (ISE) systems and their underlying mechanisms, basic working principle and heat–mass transfer mechanisms, highlighting solar energy absorption, interfacial evaporation, and water–vapor transfer.

#### 5.3.2 Material Selection and Device Optimization

The efficiency of ISE systems is engineered through advanced photothermal materials and tailored porous architectures. High light‐to‐heat conversion (efficiencies up to 90%) is achieved using broadband absorbers such as carbon‐based materials (graphene, CNTs) [198] and materials leveraging localized surface plasmon resonance (e.g., Au, Ag nanoparticles) or electron‐hole pair generation (e.g., Ti_2_O_3_, MoS_2_) [199]. In ISE, photothermal conversion is essential, as solar irradiation must be localized at the air–water interface to confine heat generation and suppress bulk water heating, thereby maximizing evaporation enthalpy efficiency [196]. Polymeric, cellulose‐based, and biomimetic materials provide scalable, hydrophilic frameworks that facilitate capillary‐driven water transfer [199, 200], while hierarchical porosity (50%–80%, with 0.1–10 µm pores) optimizes water delivery, vapor escape, and heat localization, reducing thermal conductivity (<0.5 W m^−1^ K^−1^) to minimize parasitic losses [197, 198, 199]. Here, performance is governed primarily by water uptake capacity, sorption enthalpy, vapor diffusion pathways, and thermal management of the substrate rather than optical absorption, with porous hydrophilic matrices ensuring efficient water transfer and sustained evaporative flux [201].

Surface engineering, including hydrophilic/hydrophobic patterning and multifunctional coatings, regulates water distribution and mitigates salt fouling in ISE systems [197, 198, 200, 202]. 3D printing enables precise fabrication of these complex architectures (e.g., conical shapes, distributed evaporation slots), achieving high evaporation rates (up to 8.17 kg m^−2^ h^−1^) and robust performance by optimizing the interplay of light absorption, thermal confinement, and mass transfer. The integration of insulating layers and spectrally selective absorbers (*α* ≈ 0.93, *ε* ≈ 0.07) [203] further enhances energy efficiency, positioning these material and design strategies as critical for developing durable, high‐yield ISE systems.

#### 5.3.3 Review of 3D‐Printed ISE Materials and Devices

AM has been applied to ISE since at least 2018 and has rapidly emerged as a key enabler for next‐generation evaporators by offering deterministic control over geometry, porosity, and functional integration. Across desalination and wastewater treatment ISE‐based systems, AM allows pore architectures and material compositions to be tailored in ways that directly enhance evaporation efficiency, durability, and scalability, positioning it as a viable route toward low‐cost and sustainable clean water production [204]. Rather than relying on stochastic foaming or templating, AM enables intentional design of heat and mass transfer pathways, which underpins the high performance reported across recent studies.

A representative example is the DIW‐fabricated hierarchical cellulose acetate/graphene oxide (CA/GO) evaporator reported by Tang et al. [50] which integrates millimeter‐scale chimney channels with micro‐ and nanoscale porosity through post‐printing alkali treatment, freeze‐drying, and hydration (Figure 14a). This multiscale architecture promotes chimney‐assisted airflow, rapid capillary water transfer, and efficient vapor escape, yielding evaporation rates exceeding 10 kg m^−2^ h^−1^ under 1 sun and up to 18 kg m^−2^ h^−1^ at inclined illumination (60°), these rates are reported based on the projected area of the evaporator, not the actual evaporative surface area. The apparent energy efficiency is ∼317%, which exceeds 100% due to significant environmental heat input and natural convection, not solely from absorbed solar energy. The contribution of dark evaporation (e.g., baseline evaporation without illumination) was not explicitly subtracted in these reported values; therefore, the net solar‐driven evaporation may be lower. Airflow was not externally forced but arose spontaneously from the chimney effect and natural convection. The system also exhibits enhanced solar absorption (96.6%), alongside exceptional salt rejection and long‐term stability maintained over 48 h in 3.5–10 wt% NaCl with 98.5% salt back‐diffusion, while freshwater production reached up to 95.1 kg m^−2^ day^−1^ under natural sunlight. Similar architectural principles are evident in bioinspired 3D‐printed sponges with aligned cellular microfluidic channels (Figure 14c) [205], where high porosity (∼90%) and low tortuosity enhance liquid uptake and continuous water replenishment. These systems achieve stable evaporation rates of ∼2.08 kg m^−2^ h^−1^ under 1 sun in real seawater without external energy input, supported by optimized capillary transfer and interconnected porous networks.

**FIGURE 14:**
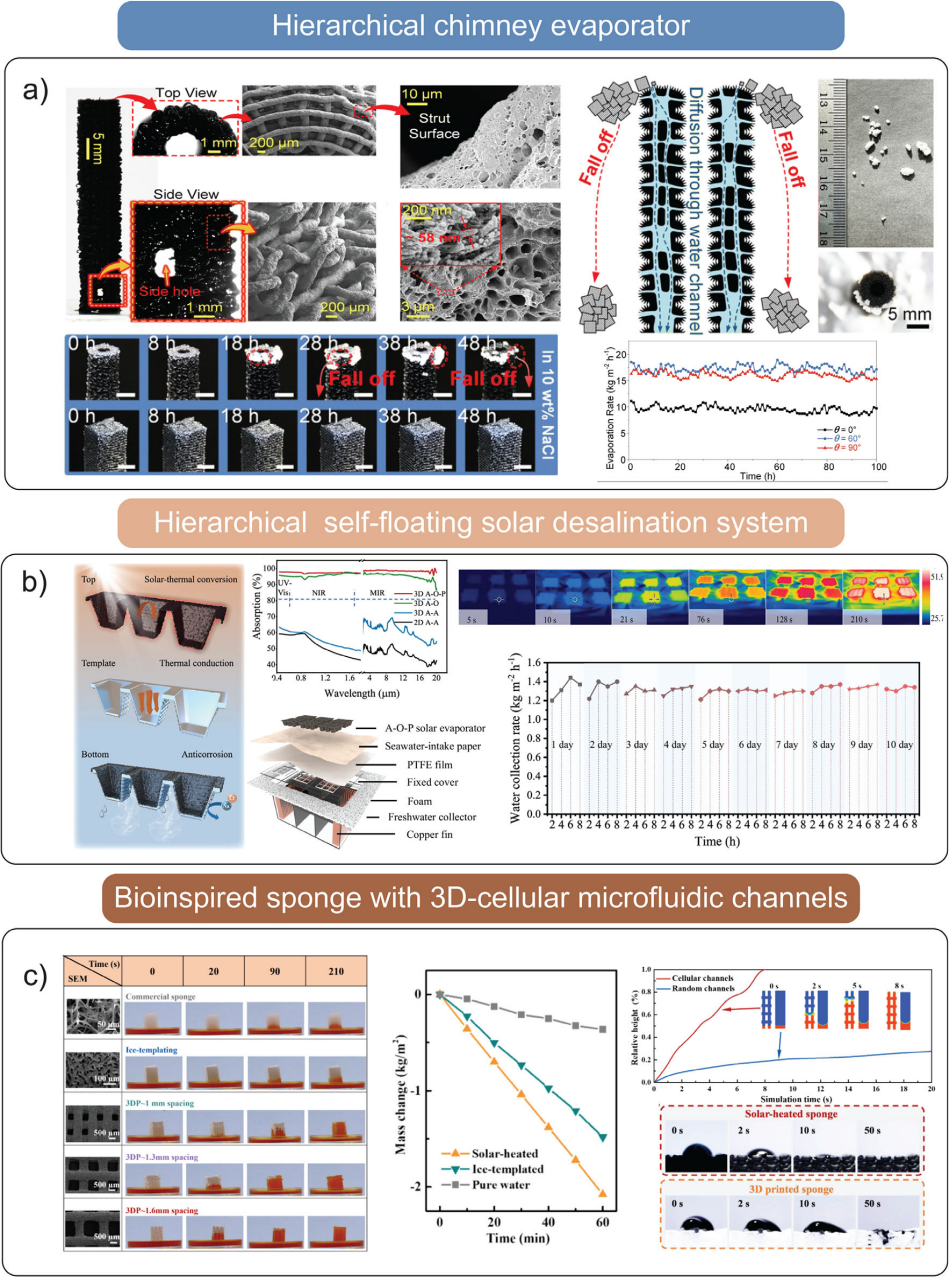
Representative 3D‐printed materials for interfacial solar evaporation (ISE) applications. (a) Photographs and SEM images of the 3D printed chimney‐type evaporator with a bottom‐to‐top channel architecture and open‐cell matrix structure filled with water. The images highlight top and side views of the porous CA–GO struts. Long‐term solar evaporation tests (1 Sun) demonstrate stable evaporation performance, effective salt rejection, and natural salt exfoliation at the chimney edges after 48 h of operation. Reproduced with permission [50]. Copyright 2025, RSC (b) Schematic illustration of the multilayer structure of a concave‐shaped solar evaporator and its scale‐up downward desalination setup. Spectral analysis shows broadband solar absorption in the UV–Vis–NIR and MIR regions. Time‐lapse thermal mapping and 10‐day freshwater collection measurements demonstrate robust long‐term desalination performance. Reproduced with permission [206]. Copyright 2025, Springer Nature (c) SEM images and absorption behavior of 3D printed sponges with different pore architectures compared to commercial sponges. Liquid absorption simulations reveal superior capillary transfer in cellular channels. Solar evaporation tests under 1 Sun show enhanced water uptake and evaporation performance of the 3D printed sponge relative to conventional materials. Reproduced with permission [205]. Copyright 2025, Elsevier.

Beyond polymeric systems, AM has also enabled hierarchical metal–polymer hybrid evaporators. Pu et al. [206] demonstrated a self‐floating desalination platform based on a 3D‐printed concave AlSi10Mg evaporator decorated with photothermal polypyrrole nanoparticles, achieving 2.23 kg m^−^^2^ h^−^^1^ under one sun with nearly 100% photothermal conversion efficiency, outperforming planar counterparts (1.86 kg m^−^^2^ h^−^^1^) (Figure 14b). The macro‐scale concave geometry increases interfacial area, while micro‐ and nano‐scale surface features enhance solar trapping and thermal reabsorption, enabling durable, corrosion‐resistant, and buoyant operation with integrated condensation collectors.

Complementary advances using light‐based AM further highlight the role of precision microstructuring. Yang et al. [47] employed DLP to fabricate hydrogel evaporators with vertically aligned micron channels (0.2–3 µm) and embedded liquid‐metal photothermal nanodroplets, achieving broadband absorption (>93%, 300–2500 nm), 2.96 kg m^−2^ h^−1^ under 1 sun (96.9% energy efficiency), and reduced vaporization enthalpy (1473 J g^−1^) (Figure 15a). The engineered semi‐IPN hydrogel matrix ensures mechanical robustness and sustained desalination performance with controlled salt crystallization.

**FIGURE 15:**
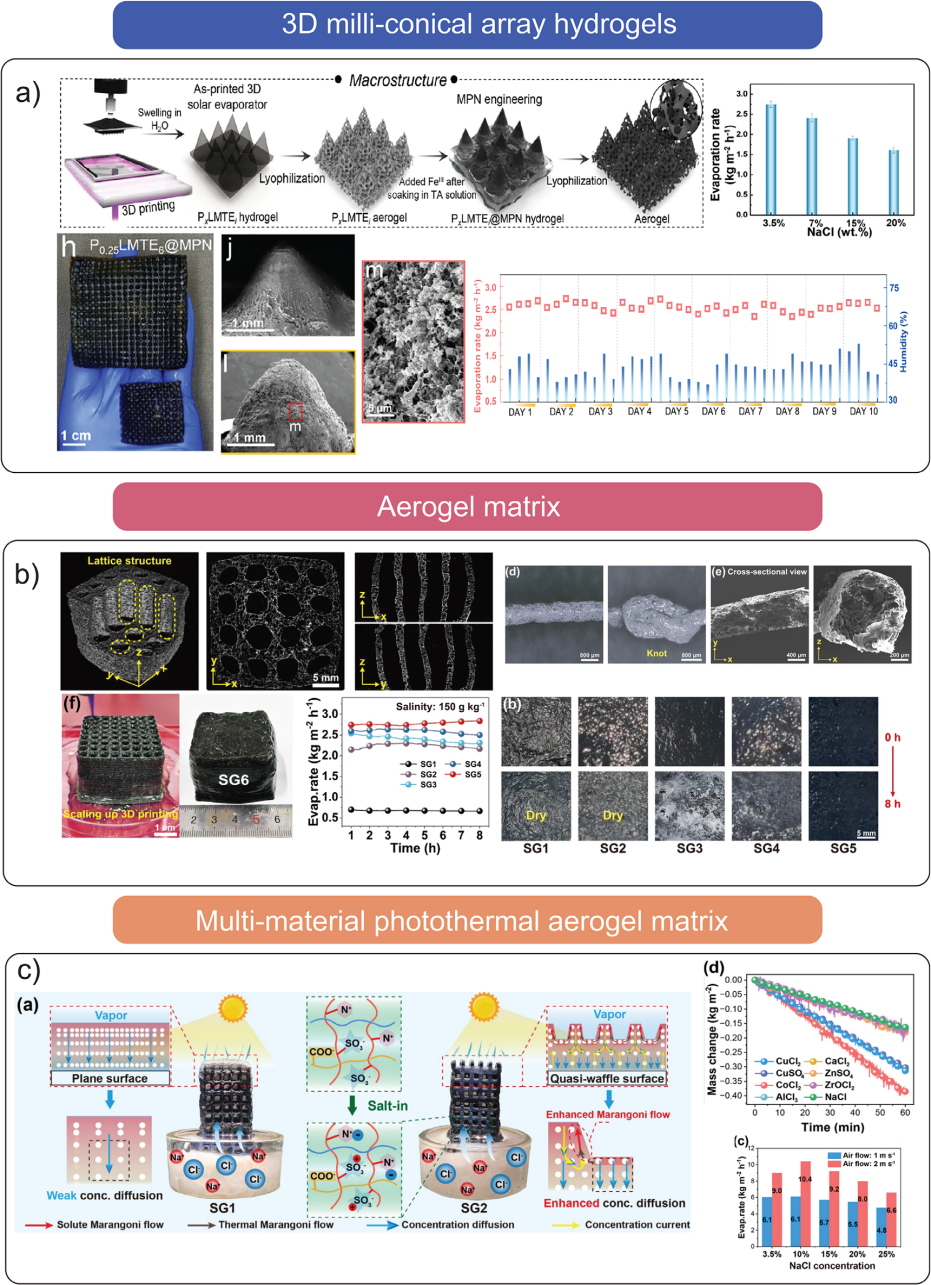
3D‐printed materials for ISE applications. (a) Schematic illustration of the microstructural evolution of MPN‐engineered 3D milli‐conical array hydrogels and corresponding optical and SEM images of single‐conical architectures. The hybrid P0.25LMTE6@MPN hydrogel evaporator exhibits stable solar‐driven evaporation and continuous desalination across varying salinities. Reproduced with permission [47]. Copyright 2024 ACS. (b) Photographs and SEM/micro‐CT images of 3D‐printed aerogel matrices (SG1–SG6) with tunable filament architectures. Solar desalination tests under 1 sun demonstrate high evaporation rates and good salt resistance, supported by visual observations of surface wettability and minimal salt sedimentation after 8 h of operation. Reproduced with permission [51]. Copyright 2024 RSC (c) The schematic in panel shows that SG2's waffle pattern enhances mass/heat flow and salt rejection, and reports record evaporation rates for SG2 (e.g., 10.4 kg m^−2^ h^−1^ in 10% NaCl at 2 m s^−1^ airflow, retaining 6.6 kg m^−2^ h^−1^ in 25% brine), and identifies Cu^2+^/Co^2+^ cations as key for crosslinking to boost evaporation, collectively demonstrating the multi‐material printed SG's superior performance under convective and high salinity conditions. Reproduced with permission [211]. Copyright 2026 Wiley‐VCH.

Meng et al. [207] demonstrated a bioinspired autonomously passive unidirectional transfer membrane (APUTM) fabricated via projection microstereolithography, replicating the hierarchical radial ridges and arch‐shaped microcavities (0.5–1.5 mm width, 70–212 µm height, 2°–8° inclination) of the Nepenthes Miranda peristome. The precisely printed millimeter‐ and microscale cavity geometry enables gravity‐free, high‐speed unidirectional water transfer and selective vapor purification in interfacial evaporation systems, achieving 36.6% separation efficiency of the oily and volatile contaminants (UV–vis–NIR, 269 nm) while maintaining deionized water‐like purity. Post‐printing surface functionalization converts the initially oleophilic–hydrophobic surface to hydrophilic–oleophobic without disrupting the microgroove architecture, enabling efficient condensation and collection 45°C–95°C while preserving 20%–50% optical transmittance (400–2100 nm) for solar‐driven evaporation. This modular AM‐enabled design overcomes the low efficiency of purely passive systems, and the energy demands of active approaches, offering a scalable platform for high‐purity desalination and wastewater treatment.

Complementary advances in DIW further highlight the role of architected porosity in ISE. Gao et al. [51] introduced foaming photothermal inks to fabricate monolithic, ultralight aerogel matrices (density ∼0.02 g cm^−3^) with interconnected multiscale pores (4.82–7.23 µm cell walls, low tortuosity, achieving evaporation rates of 2.94 kg m^−2^ h^−1^ under 1 sun and 8.35–8.62 kg m^−2^ h^−1^ outdoors (765 W m^−2^), alongside exceptional cost‐effectiveness (1755.4 g h^−1^ $^−1^) (Figure 15b). The hierarchical porous network enhances capillary transfer, reduces effective vaporization enthalpy, and suppresses salt fouling even in highly saline brine (150 g kg^−1^), while the purified water meets WHO standards.

(a) Photographs and SEM images of the 3D‐printed chimney‐type evaporator with a bottom‐to‐top channel architecture and open‐cell matrix structure filled with water. The images highlight top and side views of the porous CA–GO struts. Long‐term solar evaporation tests (1 sun) demonstrate stable evaporation performance, effective salt rejection, and natural salt exfoliation at the chimney edges after 48 h of operation. Reproduced with permission [50]. Copyright 2025 RSC. (b) Schematic illustration of the multilayer structure of a concave‐shaped solar evaporator and its scale‐up downward desalination setup. Spectral analysis shows broadband solar absorption in the UV–vis–NIR and MIR regions. Time‐lapse thermal mapping and 10‐day freshwater collection measurements demonstrate robust long‐term desalination performance. Reproduced with permission [206]. Copyright 2025 Springer Nature. (c) SEM images and absorption behavior of 3D‐printed sponges with different pore architectures compared to commercial sponges. Liquid absorption simulations reveal superior capillary transfer in cellular channels. Solar evaporation tests under 1 sun show enhanced water uptake and evaporation performance of the 3D‐printed sponge relative to conventional materials. Reproduced with permission [205]. Copyright 2025 Elsevier.

Significantly, this performance mirrors previous studies on freeze‐dried and freeze–thaw engineered porous systems, where it was suggested that confined water and interfacial photomolecular effects could diminish the effective evaporation energy barrier and concentrate photothermal conversion at liquid–solid interfaces [208]. These investigations indicated that nanoconfinement, interfacial bonding, and structured hydrogen‐bond networks within hierarchical pores can modify water thermodynamics, allowing for apparent evaporation rates that exceed classical bulk limits [209]. However, it was not until after 2023 that this phenomenon received direct experimental and mechanistic validation through calorimetry, interfacial spectroscopy, and transfer modeling [80, 210], which elucidated the roles of confined water states, thermal localization, and reduced parasitic heat losses in porous ISE systems. This suggests that AM should not only replicate the geometric porosity achieved through freeze–drying or freeze–thaw methods but must also facilitate precise control over multiscale confinement, connectivity, tortuosity, and surface chemistry to recreate the interfacial architectures that are responsible for these energy‐localization effects. This marks a shift from empirically optimized porous absorbers to architecturally and mechanistically informed AM‐designed ISE materials [209].

Li et al. [211] made a seminal contribution to multi‐material 3D printing (Figure 15c). They used a DIW process to produce a photothermal aerogel matrix that integrates gradient pore structures (AM‐2/5/8), zwitterionic polymer (PDMAPS)‐mediated anti‐polyelectrolyte effects, and post‐printing cation cross‐linking (Ca^2+^, Cu^2+^, Co^2+^). The architecture is non‐homogeneous, which allows for enhanced water transfer through capillarity and swelling. This results in increased intermediate water content and reduced evaporation enthalpy, leading to record‐breaking evaporation rates. Under 1 sun with 2 m s^−1^ airflow, SG4 (cross‐linked with Co^2+^) achieves a water evaporation rate of 17.9 kg m^−2^ h^−1^ in seawater, which is 10.5% than in freshwater and over six times that under calm air. It maintains 6.6 kg m^−2^ h^−1^ even in 25% brine under the same convective condition. Under calm conditions, SG2 attains 2.5 kg m^−2^ h^−1^ in 3.5% NaCl and retains 2.3 kg m^−2^ h^−1^ in 25% brine. The system's apparent energy efficiency under 1 sun with 2 m s^−1^ airflow is ∼450%, thanks to environmental energy harvesting. Extensive outdoor testing has demonstrated that the system functions reliably over a span of 30 days, exhibiting an average evaporation rate of 52.1 kg m^−2^ day^−1^ on days with abundant sunlight. This process has yielded freshwater that meets the WHO standards for drinking water quality. Additionally, rearranging the same matrix units into solar crystallizers (SCs) with AM‐0 (without PDMAPS) as the middle unit enables targeted salt harvesting. This configuration achieves a salt collection rate of 55.6 g m^−2^ h^−1^ in 15% NaCl for SC2‐3. This work is the first demonstration of multi‐material printing for the flexible fabrication of SGs and SCs with the unique capability of achieving higher evaporation rates in seawater than in freshwater across all tested devices.

Across the reviewed ISE systems, pore architecture provides a consistent basis for comparison, whereas reported evaporation performance does not due to variations in testing boundaries. Table S3 summarize the datasets for direct comparison. From a pore‐scale perspective, these systems exhibit distinct architectural characteristics. Examples include macrochannel‐dominant chimney structures (millimeter scale) [50] cellular channels with millimeter spacing and high porosity (∼90%) [205], vertically aligned micron‐scale hydrogel channels (0.2–3 µm) [47], micron‐scale aerogel networks (∼4.8–7.2 µm) [51], and gradient or hybrid macro–micro/nano porous structures that enable through‐thickness transfer control [206]. These architectures consistently correspond to transfer roles, macroscale channels (>100 µm) reduce vapor‐flow resistance and enable chimney‐driven or low‐tortuosity fluid transfer [50, 205], microscale pores (≈0.5–10 µm) regulate capillary water supply and storage [47, 51], and nanoscale features (<100 nm) enhance wettability, photothermal absorption, and interfacial energy localization [206, 208].

In contrast, evaporation flux values exhibit significant dispersion, even among comparable pore regimes. Under nominal one‐sun, low‐airflow conditions, most systems cluster within ∼2–3 kg m^−2^ h^−1^ [47, 51, 205, 206], suggesting a common transfer‐limited regime. However, substantially higher values are reported for macrochannel architectures under enhanced illumination. The same is true for gradient porous systems under forced airflow and varying salinity. This highlights strong sensitivity to external mass‐transfer conditions. These differences are not solely architectural, but also reflect inconsistencies in measurement methodology. Specifically:

- Airflow is rarely controlled or reported despite its strong influence on evaporation.
- Dark evaporation is inconsistently accounted for; only selected studies report net flux [206].
- Salinity varies widely (3.5–25 wt%), which directly affects performance.
- Collection‐side metrics are not uniformly reported. Some studies report surface flux [47, 51, 205], while others report collected yield [50, 206]. These values are not directly comparable.

Evaporation flux values alone cannot be directly compared across studies without a full specification of operating conditions. The apparent performance spread reflects differences in airflow, salinity, and reporting methodology as much as it reflects differences in pore architecture. AM enables deterministic control over pore size, hierarchy, and orientation, but meaningful benchmarking of ISE performance requires standardized reporting of test conditions and a consistent distinction between gross evaporation, net evaporation, and collection‐based metrics.

## 6 Design Guidelines and Comparative Framework

The escalating demands of the global water–energy nexus call for materials in which pore architecture is not incidental but deliberately engineered to govern mass and heat transfer in AWH, RC, and ISE systems [166]. AM as a versatile platform for pore engineering, offers unprecedented control over pore size, connectivity, tortuosity, grading, and hierarchical, enabling application‐specific architectures that extend beyond the constraints of stochastic porosity typical of conventional foaming, templating, or phase‐separation methods [52]. This section synthesizes the insights from this review into a comparative and partially quantitative framework that links AM processing, pore architecture, transfer physics, and functional performance metrics across AWH, RC, and ISE. This framework aims to empower researchers and engineers to align AM selection with application‐specific requirements, effectively navigating the critical trade‐offs between sorption kinetics, thermal management, optical properties, and scalability [179].

### 6.1 A Decision Framework for AM in the Air–Water–Energy Nexus

Across the air–water–energy nexus, this review has demonstrated a rapidly expanding body of AM‐enabled proof‐of‐concept materials and devices for RC, AWH, and ISE. However, these studies largely remain disconnected demonstrations rather than manifestations of a shared quantitative design logic. In this review, we propose a unified transfer‐architecture framework that links AM processing routes, pore hierarchy, coupled heat/mass transfer, and device‐level performance across AWH, RC, and ISE. The missing link is a unifying framework that connects AM choices to pore architecture, transfer mechanisms, and ultimately device‐level performance [209, 212]. Here, we argue that next‐generation systems should abandon reliance on stochastic porosity and instead adopt application‐specific, directional, and hierarchically organized pore architectures, where pore function, not fabrication resolution, defines the design strategy. The novelty of this framework lies in treating pore architecture as the primary engineering variable across all three technologies, allowing AM methods to be compared according to transfer function rather than material class or application alone. In the ideal scenario, unconstrained by fabrication limits, AM‐enabled devices would feature function‐separated pore networks, spatially localized and hierarchically distributed across length scales. Analogous to biological systems, where roots, trunks, branches, and leaves each serve distinct transfer and energy roles, ideal architectures would decouple liquid transfer, vapor diffusion, heat localization, and optical response within a single integrated structure. Pore‐scale transfer analyses further show that graded macropore distributions (e.g., front‐loose/back‐tight architectures) can independently enhance mass transfer and reaction efficiency [213], reinforcing the principle that transfer pathways should be designed according to target permeability, diffusion length, and residence time before material selection [214]. Crucially, such architectures would be designed prior to material selection, allowing geometry and transfer pathways to dictate material choice rather than the inverse. Design frameworks for AM porous media increasingly adopt topology optimization, biomimetic tree‐like channels, and TPMS gradients to independently tune heat and mass transfer while preserving mechanical integrity [212, 215]. This decoupling would enable independent optimization capillary supply, vapor escape, thermal confinement, and mechanical stability, overcoming efficiency losses inherent to coupled, random pore systems, and provides a rational pathway toward high‐efficiency RC, AWH, and ISE platforms grounded in transfer‐governed architectural design rather than empirical porosity.

Achieving this ideal requires independent control over pore size–function relationships (Figure 16). Macropores define directional vapor and airflow pathways and govern pressure drop, directly influencing sorption depth and vapor transfer efficiency in SAWH systems [15, 16]. Micropores regulate capillary transfer and water storage and act as microfluidic channels in ISE architectures, enabling sustained water supply and wicking‐driven replenishment [47, 133]. Nanopores dominate surface wetting, sorption kinetics, and optical interactions; in RC systems, they can be tuned to specific scattering regimes to maximize solar backscattering while preserving strong mid‐infrared emissivity within the atmospheric window. Random porosity fundamentally fails in this context because it intrinsically couples these mechanisms, leading to parasitic heat losses, uncontrolled salt accumulation, and inefficient vapor transfer. In RC systems specifically, stochastic pore distributions broaden scattering spectra and introduce uncontrolled absorption pathways, which can increase solar absorption, enhance non‐radiative thermalization, and reduce spectral selectivity in the MIR spectrum ultimately negatively affecting the net cooling power. AM, by contrast, enables intentional decoupling through architectural control. At present, the field has made meaningful but incomplete progress toward this vision. DIW has emerged as the most widely used AM technique across ISE, RC, and AWH due to its material versatility, accessibility, and ability to impose macroscopic architectural features, such as aligned channels and cellular frameworks. VPP including DLP, SLA, and two‐photon polymerization, offers superior resolution and surface fidelity, enabling precise optical‐scale features and controlled surface morphologies, while SLS provides access to mechanically robust, load‐bearing architectures. Across these techniques, reported porosities typically fall in the 90%–97% range, with pore sizes spanning nano‐ to macro‐scales; however, it is critical to note that most micro‐ and nanopores arise from post‐processing rather than being intrinsic to the AM step itself.

**FIGURE 16:**
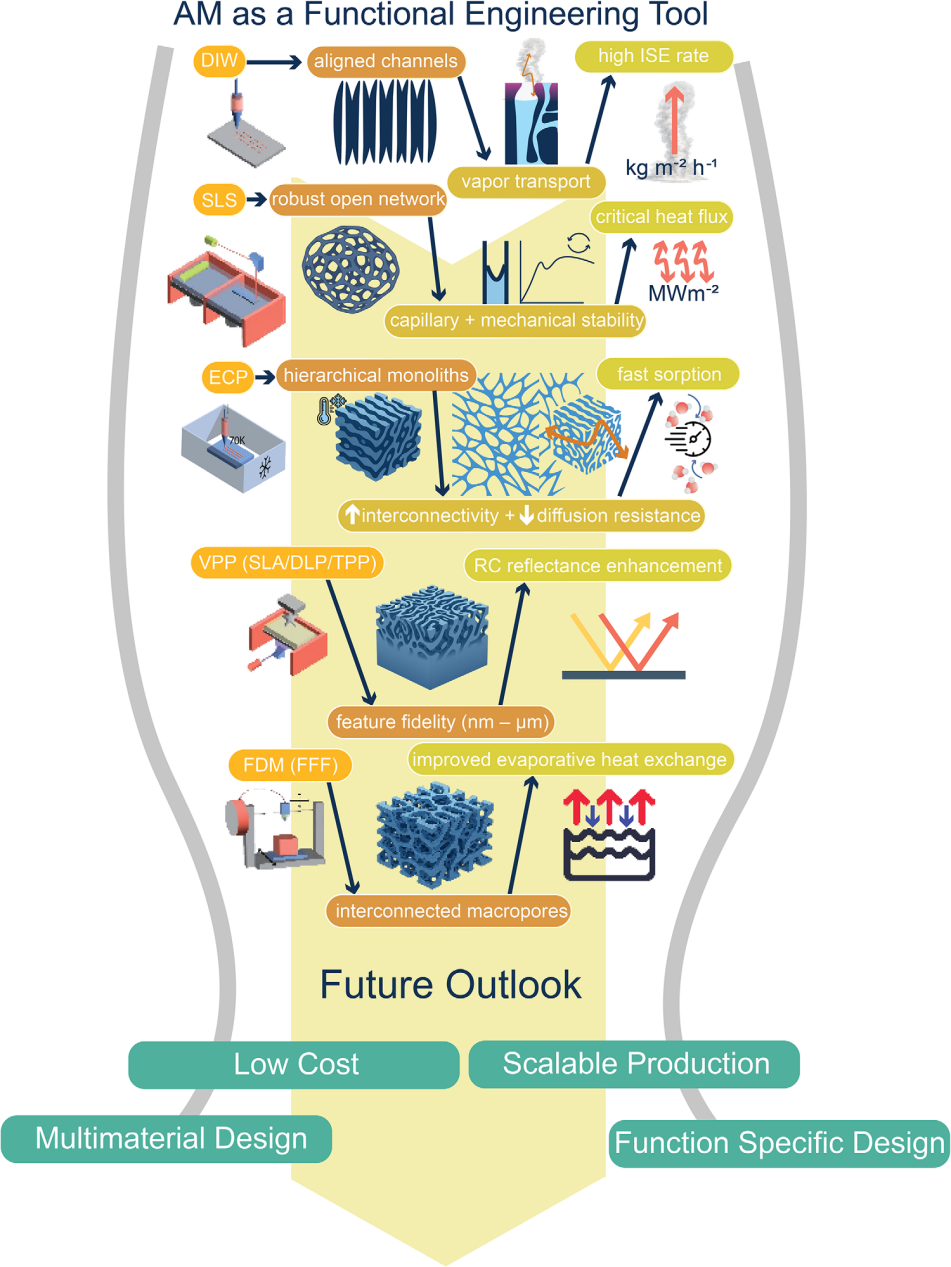
AM as a functional transfer‐engineering platform for air–water–energy systems.

Despite this limitation, several transfer mechanisms have already been successfully enhanced through AM‐enabled architectures. Chimney‐like macroscopic channels improve airflow and vapor removal in ISE systems [50], low‐tortuosity microchannel networks enhance capillary pumping and water replenishment [133], and hierarchical micro–nano features promote heat localization and broadband optical scattering (Figure 16) [44, 48]. These architectural strategies translate directly into improved performance metrics, including elevated evaporation rates, larger sub‐ambient cooling, increased freshwater yield, and enhanced durability under prolonged operation. Collectively, these results confirm that architecture‐driven transfer control, rather than material novelty alone, underpins the success of current designs.

Nevertheless, a substantial gap remains between the state of the art and the ideal scenario [216]. Key pore characteristics, including pore shape, tortuosity, connectivity, and spatially resolved gradients, are rarely controlled directly through AM and are often insufficiently reported. Limitations intrinsic to AM, such as trade‐offs between resolution and build scale, printing speed, material compatibility, and surface roughness, further constrain achievable architectures [217]. Post‐processing introduces additional challenges, including shrinkage, pore collapse, loss of orientation, and an unavoidable trade‐off between mechanical robustness and extreme porosity [218]. As a result, most studies rely on case‐by‐case optimization, report inconsistent pore metrics, and lack a shared architectural framework that would enable generalizable design rules.

Closing this gap requires a conceptual shift in how AM is employed. Rather than acting primarily as a pore generator, AM should be leveraged as a pore organizer, defining the spatial arrangement, orientation, and hierarchy of pores, while compatible post‐processing routes introduce fine‐scale porosity. By integrating porogen strategies, freeze‐drying, foaming, or sintering directly into the design phase, AM can prescribe a target transfer architecture that is subsequently realized through controlled material transformations. In this context, AM technique selection should be explicitly linked to dominant transfer requirements: DIW for vapor transfer and multiscale architectural control, VPP for optical‐scale precision and surface engineering, and SLS for mechanically durable, load‐bearing components.

To enable meaningful comparison and accelerate progress, future studies must adopt standardized, architecture‐aware metrics. Beyond bulk porosity and average pore size, reporting should include pore size distributions, orientation factors, effective diffusivity, thermal conductivity, porosity relationships, optical characteristics, and long‐term operational stability. Such metrics would allow transfer mechanisms to be quantitatively linked to measured performance, transforming isolated demonstrations into a coherent design space. Looking forward, next‐generation AM‐enabled water–energy systems will be multi‐material, functionally graded, and explicitly application‐specific, integrating SAWH, ISE, and RC functionalities within unified architectures. Progress over the next couple of years will depend on coupling architectural design with predictive modeling, developing standardized AM‐compatible fabrication routes across material classes, and advancing hybrid systems that exploit shared transfer pathways. The central takeaway of this review is that AM should be viewed as a transfer‐pathway engineering platform rather than simply a pore‐generation tool. This manufacturing‐ and architecture‐centric perspective provides a generalized roadmap for designing next‐generation water–energy systems independent of any single material chemistry. When architecture is treated as a first‐class design variable, AM becomes a powerful enabler of scalable, efficient, and resilient solutions for the air–water–energy nexus.

### 6.2 Application‐Optimized AM Selection

Building on the framework in Section 6.1, AM technique selection is governed by the dominant transfer mechanism and required pore function, rather than fabrication convenience. When pore architecture is treated as the primary design variable, consistent application‐specific trends emerge:

- AWH: DIW and ECP enable hierarchically interconnected macro‐channels with micro/mesoporous sorption domains, minimizing vapor diffusion resistance while preserving mechanical stability, critical for hygroscopic composites and MOF monoliths where uptake kinetics and regeneration depend on pore connectivity.
- RC: VPP and DIW provide precise nano‐ to microscale surface structuring and semi‐closed porosity required for wavelength‐matched Mie scattering and suppressed conductive heat transfer, supporting high solar reflectance and mid‐IR emissivity in scalable cooling surfaces.
- ISE: DIW and SLS/SLM enable open, vertically aligned macroporous networks that enhance capillary pumping, reduce viscous losses, and mitigate salt fouling, making them well suited for photothermal composites and structurally robust metal evaporators.

These trends reinforce the central conclusion of Section 6.1, where AM should be selected based on the pore functions and transfer physics, based on an application demands, not solely on material compatibility or print resolution.

### 6.3 Path to Validation and Implementation

Table 2 serves as the operational core of the proposed framework, translating the design logic developed in Section 6.1 into an application‐ready selection map. It consolidates how target pore characteristics, material requirements, and AM techniques align with dominant transfer mechanisms in AWH, RC, and ISE. In this sense, the table functions as both a validation reference and a decision guide for future system design.

**TABLE 2:** Comparative framework for selecting AM techniques in the air–water–energy nexus, summarizing optimal pore characteristics, material requirements, and recommended AM methods for different application domains, linking structural design choices with targeted performance outcomes.

Application	Target pore characteristics	Key material requirements	Recommended AM method	Quantitative performance metrics
AWH	Micro/mesopores dominant (0.2–50 µm), high interconnectivity, hierarchical (macro + micro/meso + nano)	Hygroscopic salts (LiCl, CaCl_2_), MOFs, hydrophilic polymer/aerogel/xerogel matrix, moderate thermal conductivity	DIW (granular/hydrogel inks), ECP, VPP (MOF monoliths)	• Effective diffusivity (*D*_eff_) • Adsorption/desorption time (*τ*, min) • Water productivity per unit energy (L kWh^−1^) • Equilibrium water uptake (g g^−1^)
RC	Nano to mesopores (1–50 nm) for scattering + controlled micro/macropores (1–500 µm) for insulation; often closed or semi‐closed cells	High‐bandgap scatterers (SiO_2_, ZnO, Al_2_O_3_), low‐absorption polymers (CNF, PE, PMSQ), hydrophobic coatings	DIW (reactive inks), VPP, FDM (hollow particles)	• Solar reflectance (*R*_solar_ > 0.95) • 8–13 µm emittance (*ε*_MIR_>0.90) • Nonradiative heat transfer coefficient (*h*_nonrad_ W m^−2^ K^−1^) • Net cooling power (*P*_cool_, W m^−2^)
ISE	Hierarchical: macro‐channels (100 µm–3 mm) + open mesopores (10–500 µm) + nano‐roughness (<100 nm)	Photothermal absorbers (GO, CNTs, PPy, LMTE), hydrophilic skeletons (CA, CNF, hydrogels), antifouling coatings	DIW (dominant), SLS/SLM (metals), DLP, FDM	• Net evaporation flux (after dark correction, kg m^−2^ h^−1^) • Dark evaporation rate (baseline, kg m^−2^ h^−1^) • Salinity range (wt% or g kg^−1^) • Condensation collection efficiency (%, or L m^−2^ day^−1^)

The pore size regimes summarized in Table 2 arise directly from governing transfer physics. By explicitly linking pore‐scale architectural parameters to measurable outputs such as effective diffusivity, evaporation flux, cooling power, and adsorption/desorption kinetics, the framework moves beyond qualitative material comparisons toward engineering‐oriented design criteria. In AWH, diffusion‐limited vapor uptake necessitates micro‐ and mesoporous domains for high sorption potential, while AM‐defined macrovoids reduce tortuosity and accelerate vapor access and regeneration. In RC, nanopores matched to optical wavelengths maximize Mie scattering and solar reflectance, whereas closed or semi‐closed macro‐porosity suppresses conductive heat transfer and enhances mid‐IR emission. In ISE, vertically aligned macropores minimize viscous resistance for water delivery and vapor escape, while mesopores stabilize thin water films via capillary action, collectively enabling high evaporation flux and salt tolerance. AM is therefore critical for organizing them directionally and hierarchically in ways inaccessible to stochastic fabrication routes.

To move from proof‐of‐concept demonstrations toward deployable technologies, validation must therefore extend beyond reporting porosity and average pore size and instead incorporate system‐level operating conditions and device‐scale performance metrics. Architecture‐aware metrics such as: pore interconnectivity, tortuosity, orientation, and hierarchical order, must be quantified alongside thermal conductivity, spectral emissivity, and absorptance. Equally important are manufacturing metrics, including print fidelity, reproducibility, scalability, and long‐term durability under cyclic thermal, humid, and saline conditions. Equally important are environmental boundary conditions such as airflow, humidity, solar irradiance, salinity, and condensation efficiency, which strongly influence real‐world water productivity and cooling performance. When evaluated against these criteria, Table 2 provides a clear pathway for translating AM‐enabled pore architectures into robust, application‐specific solutions for the air–water–energy nexus. Although current literature remains too heterogeneous to establish universal scaling laws, the framework presented here defines the measurable architectural and transfer parameters necessary for future predictive and data‐driven modeling.

## 7 Challenges, Research Gaps, and Future Outlook

The path from laboratory innovation to widespread global impact poised by AWH, ISE, RC, is burdened with persistent challenges. Device‐level performance continues to be governed by coupled trade‐offs between material architecture, environmental operating conditions, heat/mass transfer, and system integration. Fabrication limitations, including the brittleness of VPP parts, the post‐processing requirements of SLS, the nozzle‐resolution constraints of DIW, and the energy‐intensive sintering and freeze‐drying steps, remain significant barriers for scalability and cost‐effectiveness. Furthermore, application‐specific hurdles, such as mitigating parasitic heat gains and vapor condensation in humid climates for RC and achieving efficient mass transfer in ISE without sacrificing mechanical integrity, demand focused attention. In addition, a lack of classification of material properties and understanding of pore level interaction is evident and presents a hurdle when deciding on the material properties needed for specific applications. To overcome these obstacles and bridge the identified research gaps, a concerted, interdisciplinary effort, powerfully augmented by artificial intelligence (AI), is essential. Future progress hinges on moving beyond phenomenological studies to a fundamental, predictive understanding of processes at all scales.

To address these gaps, we present three critical research directions in which AI can accelerate progress:

- AI‐Driven Multi‐Scale Modeling and Inverse Design: The intrinsic coupling of heat transfer, vapor diffusion, capillary flow, and radiative transfer within hierarchical porous media presents a formidable challenge for conventional analytical and numerical modeling. Recent efforts have begun integrating machine learning with transfer simulations in water–energy materials, including data‐driven optimization of sorbent structures for SAWH [219], AI‐assisted pore‐structure optimization in solar‐driven interfacial evaporation [220] and computational inverse design of spectrally selective radiative cooling surfaces [221, 222]. These early demonstrations indicate that data‐driven approaches can accelerate structure–property mapping beyond brute‐force parametric sweeps. Graph neural networks provide a particularly natural representation for porous media because they map pore networks into graphs, where pores or cells act as nodes and throats or connectivity elements as edges, enriched with physically meaningful attributes such as pore volume, depth, pressure, and saturation [223]. Message‐passing operations then propagate local connectivity information into latent embeddings that can be used to predict permeability, transfer behavior, or drainage patterns in complex irregular networks. Looking forward, physics‐informed neural networks (PINNs) and deep learning models trained on high‐fidelity multi‐physics simulations combined with experimental datasets could predict coupled fluid–thermal–radiative transfer in 3D‐printed porous architectures with unprecedented speed and generalizability [224, 225, 226, 227]. Beyond simulation and prediction, generative AI, and reinforcement learning may guide inverse design of optimal architectures for specific objectives. These systems could autonomously generate novel, non‐intuitive architectures (e.g., complex TPMS or bio‐inspired lattices) that are explicitly tailored to maximize a target function, such as water production rate per energy input or specific cooling power [228]. At the nanoscale, where confinement effects dominate, water no longer behaves as a continuum fluid in pores below ∼10 nm. In particular, continuum assumptions break down at approximately 1.5 nm pore diameter due to local layering, slip, and interatomic interactions [229]. In this regime, density functional theory (DFT)‐based methods become essential because they explicitly capture the polarizability of water, solutes, and confining solids, enabling accurate prediction of electrostatic energies and binding trends [230]. Coupled DFT–SAFT formulations can further extend this atomistic fidelity into computationally efficient pore‐pressure maps, making them valuable building blocks for multiscale models and digital‐twin frameworks.
- Intelligent Material and Process Innovation: The development of next‐generation functional devices will be accelerated by AI that bridges the gap between material chemistry, process parameters, and final performance [221, 231]. ML algorithms could analyze vast compositional libraries and identify promising new hybrid materials (e.g., predicting the ideal ratio of MOFs to hydrogels for kinetics and capacity. Adopting composite materials that combine complementary functional properties will allow the development of multifunctional devices with enhanced performance and efficiency. For AM, random forest models [232] provide an interpretable pathway linking process parameters to performance outcomes. These models, trained on engineered features extracted from finite‐element or manufacturing datasets, use ensemble learning strategies and improve robustness via out‐of‐bag predictions, while feature importance can be quantified using Gini‐based metrics and predictive measures of association. Such frameworks have been demonstrated in AM and related manufacturing systems for identifying which process variables most strongly influence structural or functional outputs. In this context, AI‐powered control systems could optimize AM processes in real‐time, adjusting printing parameters like laser power (SLS), nozzle speed (DIW), or UV exposure (VPP) to correct for defects and ensure pore morphology fidelity, thereby overcoming current limitations in resolution and reproducibility [233, 234, 235].
- Data‐Driven System Integration and Validation: For AM‐enabled water–energy devices, commercialization requires not only performance optimization but architecture‐specific validation under realistic operating conditions. Because AM produces deterministic, digitally defined pore networks, AI can directly correlate long‐term performance metrics (e.g., cyclic stability, salt accumulation, degradation rates) with both environmental inputs (humidity, temperature, solar irradiance) and architectural descriptors (pore size gradients, channel spacing, tortuosity). Such data‐driven analysis enables the creation of digital twins of 3D‐printed systems [236], transforming prototype testing into predictive lifecycle modeling and guiding iterative architectural refinement. Such approaches are especially important because evaporation flux, freshwater yield, and cooling power are highly sensitive to environmental conditions and system‐level heat and mass exchange, not solely intrinsic material properties. At the system level, AI can further optimize operation strategies, such as dynamically switching sorption/desorption cycles in SAWH or balancing thermal fluxes in hybrid SAWH/RC platforms, based on both forecasted environmental conditions and the specific thermal–mass transfer characteristics imposed by the printed architecture

In conclusion, although significant challenges remain, the potential of AM to revolutionize the approach to water and energy security is undeniable. The integration of AI is not merely an incremental improvement but a paradigm shift that will accelerate the entire innovation cycle from fundamental discovery to functional material design and system‐level optimization. By leveraging AI‐driven digital design tools to generate optimized porous architectures, incorporating composite or hybrid materials to achieve multifunctionality, and systematically characterizing pore‐level processes and material responses, it becomes possible to engineer technologies that operate efficiently and sustainably under real‐world conditions. By bridging the gaps between computational science, materials science, and engineering and chemistry, AM with a synergetic implementation of AI can drive unprecedented innovation in the energy‐water‐air nexus, directly contributing to the achievement of global sustainability goals like United Nations Sustainable Development Goal 6 that aims to ensure availability and sustainable management of water and sanitation for all by 2030. Ultimately, the integration of rational material design, precise AM, and intelligent system control provides a clear pathway toward the next generation of resilient and sustainable water and energy harvesting technologies.

## Author Contributions

**Dejan J. Trajkovski**: writing – original draft, visualization, software, investigation. **Higgins M. Wilson**: validation, formal analysis. **Jiaxing Xu**: investigation, methodology. **Abdallah Y. M. Ali**: software, resources. **Ruzhu Wang**: formal analysis, funding acquisition, supervision. **Primož Poredoš**: conceptualization, formal analysis, funding acquisition, project administration, supervision, writing – review and editing.

## Conflicts of Interest

The authors declare no conflicts of interest.

## Supporting information




**Supporting File**: adma74850‐sup‐0001‐SuppMat.pdf.

## Data Availability

The data that support the findings of this study are available from the corresponding author upon reasonable request.
