# Supplementary material for: Toward Pore‐Engineered 3D‐Printed Materials for Sorption Water Harvesting, Interfacial Evaporation, and Radiative Cooling
Source: Adv Mater. 2026 Aug 31;38(61):e74850. doi: 10.1002/adma.74850 (PMC13726699; doi:10.1002/adma.74850)
Supplement: Supplementary file 1 [file ADMA-38-e74850-s001.pdf]

Supporting Information

Toward Pore-Engineered 3D Printed Materials for Sorption Water Harvesting, Interfacial Evaporation and Radiative Cooling

Dejan J. Trajkovski[1], Higgins Marangattil Wilson[1], Abdallah Y. M. Ali[1], Jiaxing Xu[2], Ruzhu Wang[2]*, Primož Poredoš[1]*

[1]Laboratory for Sustainable Technologies in Buildings, Faculty of Mechanical Engineering, University of Ljubljana, Aškerčeva cesta 6, 1000 Ljubljana, Slovenia

[2]Institute of Refrigeration and Cryogenics, Engineering Research Center of Solar Energy (MOE China), Shanghai Jiao Tong University, 800 Dongchuan Road, 200240 Shanghai, China

*Corresponding Authors: primoz.poredos@fs.uni-lj.si; rzwang@sjtu.edu.cn

Table of Contents:

Table S1

Table S2

Table S3

Table S4

Table S5

Table S6

Table S1: SAWH performance of state-of-the-art 3D-printed sorbents: pore architectures, including vertically aligned channels and lattice structures with wrinkled surfaces, are compared alongside porosity, effective diffusivity (where available), adsorption/desorption kinetics, water productivity, equilibrium water uptake, and notable functionalities such as superelasticity and shape-memory behavior.

**Reference**	**Pore architecture / size**	**Porosity (%)**	**Effective diffusivity d_eff ($m^2 s^{-1}$)**	**Adsorption time τ_{ads}**	**Desorption time τ_{des}**	**Water productivity ($l\ kWh^{-1}$)**	**Equilibrium water uptake ($g\ g^{-1}$)**	**Additional features**
[1]	3D-printed mm-scale vertical channels (0.6 × 0.6 mm^2) + µm crack-like pores	NR	NR	88.4% equilibrium in 120 min; initial rate 1.50 $g\ g^{-1}\ h^{-1}$	81.8% release in 20 min (2 $m\ s^{-1}$ airflow, 11 V heating)	~178 (array-based, estimated; not directly scalable)	1.18 (90% RH, 2 h); ~1.32 equilibrium	High-rate cyclic sorption; airflow-assisted desorption
[2]	mm lattice channels + µm wrinkled surfaces + nm granular HMP network	80	NR	2.85 $g\ g^{-1}$ (90% RH, 48 h)	97.6% release in 2 h at 80 °C	NR	2.85 (90% RH); 1.09 (70% RH); 0.62 (60% RH); 0.38 (50% RH)	4×4 lattice reduces material usage by 53%
[3]	mm-scale printed grid (~0.6 mm) + µm freeze-dried pores	NR	NR	1.59 $g\ g^{-1}$ (95% RH, 12 h); rate 0.13 $g\ g^{-1}\ h^{-1}$	Not fully quantified	Not directly reported (solar-driven system)	1.65 (95% RH); 0.39 (11% RH)	1.6× faster sorption; 2.4× faster desorption vs aerogel
[4]	Printed grid (0.9 mm lines, 20–30% infill) + freeze-dried micropores	NR	NR	2.6 $g\ g^{-1}$ (95% RH, ~25 h); initial rate 2.08 % min^{-1}	Not reported	NR	2.6 (95% RH); 0.56 (43% RH)	$CaCl_2$-dependent swelling and superelasticity
[5]	200 nm–3 µm open-cell HIPE-derived pores + macroporous scaffold	~85	NR	Not measured for water vapor (organic solvent uptake 15.5 $g\ g^{-1}$)	Not measured	NR	N/A (non-water vapor system focus)	Thermo-responsive shape memory behavior

Table S2: RC performance of additively manufactured radiative cooling materials: pore size, porosity, refractive index contrast (e.g., medium/high), and hierarchical structural organization are compared alongside solar reflectance, 8–13 µm emissivity, net cooling power, temperature reduction, and additional functionalities including thermal insulation and hydrophobic behavior.

**Reference**	**Pore architecture / size**	**Porosity (%)**	**Refractive-index contrast**	**Hierarchical organization**	**Solar reflectance (%)**	**8–13 µm emissivity (%)**	**Net cooling power ($W m^{-2}$)**	**Temperature drop (°C)**	**Additional features**
[6]	1–6 µm pores + mesopores; 3D lattice spacing 500 µm	97.3	Medium (PMSQ/CNF vs. air)	3D-printed lattice + nanoporous aerogel	94.2	95.6	72.2 (day); 130.2 (night)	5.8	Thermal conductivity 32.3 $mW m^{-1} K^{-1}$; hydrophobic (CA 142.3°)
[7]	~40 nm mesopores + 0.824 mm axial channels	NR	Medium (CNF/SiO_2 vs. air)	Wood-like channels + mesopores + SiO_2 nanoparticles	94.18	96.09	NR	6.16	Thermal conductivity 59.4 $mW m^{-1} K^{-1}$; electronic cooling
[8]	0.7–1.0 µm (PDMS), 5–7 µm (PES); Ag@ZTO nanorods (100–150 nm)	NR	High (Ag/ZTO vs. polymer/air)	Bilayer wettability gradient + nanorods	89.5	91.8	NR	19.3	Unidirectional water transport; breathable fabric
[9]	Microrods: radius 50 µm, height 400 µm, spacing 300–500 µm + ZnO nanoparticles (~30 nm)	NR	High (ZnO vs. air/polymer)	Microrods + nanospheres/nanobelts	70.3	96.95	152.4 (day); 159.4 (night)	~21	Superhydrophobic (CA 164.7°); water harvesting 2018.5 $mg cm^{-2} h^{-1}$
[10]	~1.52 µm pores + Al_2O_3 nanoparticles (~530 nm)	NR	Medium (TPU/Al_2O_3 vs. air)	Microhumps + internal pores + nanoparticles	97.6	95.5	78	6.6	Biomimetic cicada-inspired structure; hydrophobic after fluorination
[11]	200 nm–2 µm nanopores	NR	Low-to-medium (PE/SiO_2 vs. air)	Bilayer SiO_2 concentration gradient	96.2	>90	85	6.1	3D-printable PE/SiO_2 composite; low-cost fabrication

Table S3: ISE performance of interfacial solar evaporation systems: pore architectures are compared alongside evaporation flux under both static and airflow-assisted conditions, dark evaporation corrections, salinity and feed-water composition, and collection-side metrics including freshwater productivity and salt collection rates.

Reference	Pore architecture / size	Evaporation flux (kg m^{-2} h^{-1})	Net flux (kg m^{-2} h^{-1})	Dark evaporation correction	Salinity / feed	Collection-side metric
[12]	Gradient pores: 126.8 → 11.4 µm; lattice spacing 3.2 mm	Airflow (2 m s^{-1}): 17.9 (seawater); 10.4 (10% NaCl); 6.6 (25% NaCl) Calm: 2.5 (3.5% NaCl); 2.3 (25% NaCl)	Not reported	Not reported	3.5–25% NaCl; seawater	Salt collection: 55.6 g m^{-2} h^{-1} (15% NaCl); outdoor evaporation ≈52.1 kg m^{-2} day^{-1}
[13]	Cellular channels: 1.0–1.6 mm spacing; ~90% porosity	2.08 (solar-heated sponge)	Not reported	Control mentioned (value not reported)	Not specified (high salinity indicated)	Not reported
[14]	Vertically aligned channels: 0.2–3 µm	2.96 (best case); 1.60 (20 wt% NaCl)	Not reported	Control present (not quantified)	3.5–20 wt% NaCl; seawater	Not reported
[15]	Porous aerogel; cell walls 4.82–7.23 µm	2.94	Not reported	Not reported	15 wt% NaCl; seawater	Not reported
[16]	Macro concave + micro/nano hierarchical surface	2.23 (3.5% NaCl); 2.08 (25% NaCl)	Same as reported (net)	Yes (dark evaporation subtracted)	3.5% NaCl; 25% brine; seawater	Freshwater collection: 1.23 kg m^{-2} h^{-1} (1 sun)
[17]	Hierarchical: mm-scale channels (3 mm central, 1.6 mm side); microcavities (0.5–10 µm); nanochannels (~58 nm)	10.2 (0°); 18.1 (60°); 16.3 (90°); 14.8 (3 sun)	Not reported	Not reported	3.5% and 10% NaCl; seawater	Freshwater yield: 86.9–90.9 kg m^{-2} day^{-1} (indoor); 95.1 kg m^{-2} day^{-1} (outdoor) (~3.6–4.0 kg m^{-2} h^{-1}equivalent)

Table S4: SAWH structure–property correlations: additive manufacturing parameters, including nozzle diameter, infill density, freeze-drying conditions, and crosslinking strategies, are correlated with structural characteristics such as pore size, pore connectivity, and tortuosity, and subsequently linked to transport mechanisms including capillary diffusion, Kozeny–Carman permeability, and effective diffusivity. Resulting functional outcomes, including accelerated sorption kinetics, enhanced water uptake, and effective salt confinement, are summarized.

AM pathway	AM control parameter / fabrication strategy	Structural outcome	Transport / thermodynamic mechanism	Functional outcome	Representative studies
**AM → pore size control → capillary diffusion & sorption kinetics**	DIW nozzle size, ink rheology, freeze-drying rate, emulsion templating, crosslinking density	Pore sizes from nanometer (200 nm) to micrometer (1–600 μm); aligned channels; crack-like micropores	Pore radius governs capillary pressure and diffusion regime (Washburn-type imbibition; capillary condensation in mesopores)	Accelerated water uptake kinetics (up to 2.1× increase), enhanced RH response, tunable sorption/desorption rates	[1], [4], [5]
**AM → porosity control → permeability & sorption capacity**	Infill density, freeze-drying, solvent exchange, salt/hydrogel loading, jammed microgel assembly	High porosity networks (~80–85%); lightweight open-cell structures; hierarchical void space distribution	Permeability scales with $\varepsilon^3/(1-\varepsilon)^2$ (Kozeny–Carman); increased void fraction enhances vapor diffusion and water uptake	High sorption capacity (up to 260 wt% water uptake), reduced material usage, enhanced vapor permeability	[2], [3], [5]
**AM → tortuosity control → effective diffusivity (d_{eff})**	Toolpath alignment (DIW), vertical channel printing, freeze-casting directionality, lattice design	Low-tortuosity aligned channels vs. disordered freeze-dried networks	Effective diffusion follows $D_{eff} = D_{bulk}\, \varepsilon\, \tau^{-1}$; reduced τ minimizes transport resistance	Faster sorption/desorption kinetics (1.6×–2.4× improvement), enhanced dehumidification rates	[1], [2], [3]
**AM → connectivity control → fluid transport continuity & salt management**	DIW lattice design, microgel jamming, freeze-drying preservation of open-cell networks	Interconnected percolating pores; open-cell architectures; continuous capillary pathways	Connectivity enables continuous liquid/vapor pathways and prevents diffusion bottlenecks	Enhanced water transport, stable cyclic sorption, suppressed salt leakage (“salt-in” confinement effect)	[1], [3], [5]
**AM → surface area enhancement → adsorption capacity & interfacial kinetics**	Nanofibril assembly (CNF/CNC), hierarchical DIW lattices, freeze-drying-induced nanostructuring, nanoparticle incorporation	Multiscale roughness (mm–nm); high BET surface area networks; granular or fibrillar microstructures	Increased adsorption sites and interfacial water binding; enhanced physisorption and multilayer water uptake (BET-type behavior)	Higher water uptake (1.4×–5.8× increase), faster RH response, improved sorption capacity and cycling stability	[2], [3], [4]

Table S5: RC structure–property correlations: additive manufacturing parameters, including ink rheology, freezing kinetics, nanoparticle incorporation, and 4D printing strategies, are correlated with structural features such as nanoporous architectures, aligned channels, and refractive index gradients. The resulting optical and thermal mechanisms, including Mie scattering, phonon–polariton resonances, and suppressed thermal conduction, are linked to performance metrics encompassing high solar reflectance, low thermal conductivity, and adaptive radiative cooling behavior.

AM pathway	AM control parameter / fabrication strategy	Structural outcome	Optical / thermal mechanism	Functional outcome	Representative studies
**AM → pore size control → solar reflection**	DIW ink rheology, freezing rate, MTMS crosslinking, phase separation, CAD-defined microrod geometry, templated microimprinting	Pores spanning 40 nm to 6 µm; microrods (300–500 µm spacing); aligned channels; microhumps	Pore sizes comparable to solar wavelengths maximize Mie scattering and internal reflection	Solar reflectance increased to ~94–98%	[6], [7], [9], [10], [11]
**AM → high porosity → thermal insulation**	Freeze-drying, solvent exchange, phase separation, sacrificial pore formation	Highly porous architectures (up to 97.3%); tortuous conduction pathways; lightweight cellular structures	Reduced solid conduction and suppressed parasitic conductive/convective heat transfer ($P_{cond+conv}$)	Low thermal conductivity (down to 32.3 mW m^{-1} K^{-1}) and enhanced net cooling power	[6], [7], [8], [11]
**AM → refractive-index contrast → scattering and mir emissivity**	Incorporation of ZnO, SiO_2, Al_2O_3, Ag@ZTO nanoparticles; air–polymer interfaces	High-index inclusions embedded within porous polymer matrices	Stronger electromagnetic discontinuities increase solar scattering and phonon–polariton resonances in the 8–13 µm region	Simultaneously high solar reflectance and high MIR emissivity (>90%)	[7], [8], [9], [10]
**AM → hierarchical organization → multispectral and multifunctional control**	DIW + freeze-drying; hydrothermal growth; VPP microrod printing; multiscale templating	Mesopores + micropores + macrochannels; nanorods + micropores; microrods + nanobelts; microhumps + nanoparticles	Different length scales independently target solar scattering, MIR emission, thermal insulation, moisture transport, and wettability	Broadband spectral selectivity, reduced parasitic losses, self-cleaning, evaporative cooling, directional liquid transport	[6], [7], [8], [9], [10]

**AM → adaptive or multifunctional architectures**	4D printing, bilayer pore gradients, responsive polymers	Dynamically reconfigurable pore exposure and wettability	Tunable emissivity and combined radiative/evaporative cooling	Environment-responsive RC and AWH functionality	[8], [18]
**AM → direct radiative cooling performance**	Combined control of pore size, porosity, refractive-index contrast, and hierarchy	Optimized spectral selectivity and suppressed thermal losses	High solar reflection + high MIR emission + low thermal conductivity	Net cooling powers up to ~152–159 W m^{-2} and sub-ambient cooling up to ~21 °C	[6], [6], [7], [8], [9], [11], [18]

Table S6: ISE structure–property correlations: additive manufacturing-enabled design parameters, including filament spacing, foaming strategies, and graded porosity, are correlated with the formation of ordered macrochannels, low-tortuosity transport networks, and hierarchical surface textures. The resulting transport mechanisms, including enhanced capillary pressure, increased permeability, and reduced evaporation enthalpy, are linked to functional improvements such as elevated evaporation flux, efficient salt rejection, and improved antifouling performance.

AM pathway	AM control parameter / fabrication strategy	Structural outcome	Governing transport / thermodynamic mechanism	Functional outcome	Representative studies
**AM → pore size control → capillary transport and diffusivity**	DIW filament spacing, nozzle diameter, DLP ink formulation, multi-material gradients, foaming-assisted printing	Ordered pores spanning ~11–127 µm; aligned mm-scale channels; hierarchical micro/nano cavities	Pore diameter controls capillary pressure and diffusion regime: smaller pores increase Laplace pressure ($P_{Lap} \propto 1/d$), while larger pores reduce flow resistance and enhance bulk diffusion	Enhanced upward water pumping, directional transport, continuous water replenishment, and improved evaporation stability	[12], [13], [14], [15], [17]
**AM → high porosity → permeability and thermal management**	Freeze-drying, phase separation, foaming inks, graded porosity printing	Highly porous open-cell structures (~73–91% porosity); lightweight cellular networks	Higher porosity increases permeability (Kozeny–Carman behavior), liquid uptake, and storage capacity while reducing solid thermal conduction	Faster water transport, improved liquid retention, lower parasitic heat loss, and enhanced evaporation efficiency	[12], [13], [14], [15]
**AM → reduced tortuosity → effective diffusivity enhancement**	Ordered toolpaths, aligned channels, gradient pore architectures, interconnected lattices	Low-tortuosity transport pathways with directional connectivity	Effective diffusivity increases with decreasing tortuosity ($D_{eff} = D_{bulk}\, \varepsilon\, \tau^{-1}$); aligned channels shorten liquid and vapor diffusion paths	Faster capillary rise, improved salt back-diffusion, reduced concentration polarization, and stable long-term operation	[12], [13], [14], [17]
**AM → interconnected pore connectivity → salt rejection and continuous transport**	DIW open-cell lattices, hydrogel freeze-drying, post-print pore opening, hierarchical channel integration	Fully interconnected macro/micro/nano pore networks	Open connectivity enables simultaneous water supply, vapor escape, and ion back-diffusion, suppressing salt accumulation	Sustained desalination under high salinity (up to 25 wt%), self-cleaning behavior, and antifouling operation	[12], [14], [15], [17]
**AM → increased surface area → reduced evaporation enthalpy and enhanced absorption**	Hierarchical texturing, nanoparticle incorporation, phase separation, concave macrostructures	High internal surface area with micro/nano textures and multiscale roughness	Increased interfacial area promotes intermediate water formation, lowers effective evaporation enthalpy, and enhances light trapping through multiple reflections	Higher evaporation flux, improved photothermal absorption (>96%), and increased vapor-generation efficiency	[12], [14], [16], [17]

**AM → hierarchical organization → coupled heat and mass transfer optimization**	Multi-material DIW, freeze-drying, hydrothermal growth, macro–micro/nano integration	Millimeter channels combined with micron pores and nanoscale features	Different length scales independently regulate vapor escape, capillary supply, thermal localization, and interfacial evaporation	Simultaneous enhancement of water transport, heat confinement, salt rejection, and evaporation performance	[12], [14], [15], [16], [17]
**AM → architecture-dependent evaporation performance**	Combined control of pore size, porosity, tortuosity, hierarchy, and surface chemistry	Gradient porous hydrogels, chimney evaporators, cellular sponges, concave photothermal structures	Evaporation performance emerges from the balance between capillary transport, vapor diffusion, thermal localization, airflow, and salinity conditions	Reported evaporation fluxes span ~2–18 kg m^{-2} h^{-1} depending on operating boundary conditions, with stable operation demonstrated even in high-salinity brines	[12], [13], [14], [15], [16], [17]

References:

[1] Z. Chen, X. Yang, Q. Shao, and R. Wang, “Biomimetic Porous Hygroscopic Monolith with Vertically Aligned Channels by 3D Printing for Rapid Dehumidification and Regeneration,” *Adv. Funct. Mater.*, vol. n/a, no. n/a, p. 2508512, Jul. 2025, doi: 10.1002/adfm.202508512.

[2] X. Wu *et al.*, “3D-Printed Hygroscopic Matrices Based on Granular Hydrogels for Atmospheric Water Adsorption and On-Demand Defogging,” *Adv. Funct. Mater.*, vol. n/a, no. n/a, p. e14721, doi: 10.1002/adfm.202514721.

[3] P. Zhu *et al.*, “3D Printed Cellulose Nanofiber Aerogel Scaffold with Hierarchical Porous Structures for Fast Solar-Driven Atmospheric Water Harvesting,” *Adv. Mater.*, vol. 36, no. 1, p. 2306653, Jan. 2024, doi: 10.1002/adma.202306653.

[4] Y. Chen, Z. Yu, Y. Ye, Y. Zhang, G. Li, and F. Jiang, “Superelastic, Hygroscopic, and Ionic Conducting Cellulose Nanofibril Monoliths by 3D Printing,” *ACS Nano*, vol. 15, no. 1, pp. 1869–1879, Jan. 2021, doi: 10.1021/acsnano.0c10577.

[5] M. Shahbazi, H. Jäger, R. Ettelaie, J. Chen, A. Mohammadi, and P. A. Kashi, “4D printing of poly-Pickering high internal phase emulsions to assembly a thermo-responsive shape-memory hierarchical macroporous scaffold,” *Addit. Manuf.*, vol. 81, p. 103977, Feb. 2024, doi: 10.1016/j.addma.2024.103977.

[6] T. Zhu *et al.*, “Reactive 3D printed silanized cellulose nanofiber aerogels for solar-thermal regulatory cooling,” *Compos. Part Appl. Sci. Manuf.*, vol. 192, p. 108761, May 2025, doi: 10.1016/j.compositesa.2025.108761.

[7] X. Shi *et al.*, “3D printed cellulose nanofiber/silica nanoparticle scaffolds for daytime radiative cooling,” *Addit. Manuf.*, vol. 92, p. 104392, Jul. 2024, doi: 10.1016/j.addma.2024.104392.

[8] A. Hazarika *et al.*, “Hierarchically designed 3-D printed porous nylon fabric-based personal thermoregulatory for radiative and directional wick-evaporative cooling,” *Chem. Eng. J.*, vol. 471, p. 144536, Sep. 2023, doi: 10.1016/j.cej.2023.144536.

[9] C.-L. Xu *et al.*, “3D Printing of a Durable Superhydrophobic Film for Daytime Radiative Passive Cooling and Water Harvesting,” *ACS Appl. Mater. Interfaces*, vol. 17, no. 20, pp. 30181–30196, May 2025, doi: 10.1021/acsami.5c00069.

[10] X. Liu *et al.*, “Biomimetic Photonic Multiform Composite for High-Performance Radiative Cooling,” *Adv. Opt. Mater.*, vol. 9, no. 22, p. 2101151, Nov. 2021, doi: 10.1002/adom.202101151.

[11] K. Zhou *et al.*, “Hierarchically Patterned Self-Cleaning Polymer Composites for Daytime Radiative Cooling,” *Nano Lett.*, vol. 23, no. 9, pp. 3669–3677, May 2023, doi: 10.1021/acs.nanolett.2c04069.

[12] J. Li *et al.*, “Tailor-Made Solar Desalination and Salt Harvesting from Diverse Saline Water Enabled by Multi-Material Printing,” *Adv. Mater.*, vol. 38, no. 5, p. e17244, 2026, doi: 10.1002/adma.202517244.

[13] Z. Mao *et al.*, “3D printing a cellular channel sponge for high-efficiency liquid collection and solar evaporation,” *Desalination*, vol. 604, p. 118725, Jun. 2025, doi: 10.1016/j.desal.2025.118725.

[14] S. Yang, H. Zhang, X. Sun, J. Bai, and J. Zhang, “3D-Printed Liquid Metal-in-Hydrogel Solar Evaporator: Merging Spectrum-Manipulated Micro-Nano Architecture and Surface Engineering for Solar Desalination,” *ACS Nano*, p. acsnano.3c12574, Feb. 2024, doi: 10.1021/acsnano.3c12574.

[15] J. Gao *et al.*, “Foaming photothermal inks for direct-ink writing: hierarchical design and enhanced solar-powered interfacial evaporation,” *J. Mater. Chem. A*, vol. 12, no. 11, pp. 6592–6609, Mar. 2024, doi: 10.1039/D4TA00069B.

[16] Y. Pu *et al.*, “Large-scale 3D printed fouling-resistant self-floating evaporator,” *Nat. Commun.*, vol. 16, Apr. 2025, doi: 10.1038/s41467-025-58952-7.

[17] Y. Tang *et al.*, “A 3D-printed hierarchical chimney for high-yield solar evaporation,” *Energy Environ. Sci.*, vol. 18, no. 17, pp. 8220–8231, Aug. 2025, doi: 10.1039/D5EE01591J.

[18] Y. Liu, R. Liu, J. Qiu, and S. Wang, “4D printing of thermal responsive structure for environmentally adaptive radiative cooling and heating,” *J. Adv. Manuf. Process.*, vol. 4, no. 2, p. e10107, 2022, doi: 10.1002/amp2.10107.
